# Burden of breast cancer and attributable risk factors in the North Africa and Middle East region, 1990–2019: a systematic analysis for the Global Burden of Disease Study 2019

**DOI:** 10.3389/fonc.2023.1132816

**Published:** 2023-08-01

**Authors:** Sina Azadnajafabad, Sahar Saeedi Moghaddam, Esmaeil Mohammadi, Negar Rezaei, Mohammad-Mahdi Rashidi, Nazila Rezaei, Ali H. Mokdad, Mohsen Naghavi, Christopher J. L. Murray, Bagher Larijani, Farshad Farzadfar, GBD 2019 NAME Breast Cancer Collaborators, Sina Azadnajafabad

**Affiliations:** ^1^ Non-Communicable Diseases Research Center, Endocrinology and Metabolism Population Sciences Institute, Tehran University of Medical Sciences, Tehran, Iran; ^2^ Kiel Institute for the World Economy, Kiel, Germany; ^3^ School of Medicine, Tehran University of Medical Sciences, Tehran, Iran; ^4^ Endocrinology and Metabolism Research Center, Endocrinology and Metabolism Clinical Sciences Institute, Tehran University of Medical Sciences, Tehran, Iran; ^5^ Institute for Health Metrics and Evaluation, University of Washington, Seattle, WA, United States; ^6^ Department of Health Metrics Sciences, School of Medicine, University of Washington, Seattle, WA, United States

**Keywords:** breast cancer, epidemiology, risk factor, Global Burden of Disease, Middle East, North Africa

## Abstract

**Background:**

Breast cancer (BC) is the most common cancer in women globally. The North Africa and Middle East (NAME) region is coping hard with the burden of BC. We aimed to present the latest epidemiology of BC and its risk factors in this region.

**Methods:**

We retrieved the data on BC burden and risk factors from the Global Burden of Disease Study 2019 to describe BC status in the 21 countries of the NAME region from 1990 to 2019. We explored BC incidence, prevalence, deaths, disability-adjusted life years (DALYs), and attributable burden to seven risk factors of female BC, namely, alcohol use, diet high in red meat, low physical activity, smoking, secondhand smoke, high body mass index, and high fasting plasma glucose. Decomposition analysis on BC incidence trend was done to find out the contributing factors to this cancer’s growth.

**Results:**

In 2019, there were 835,576 (95% uncertainty interval: 741,968 to 944,851) female and 10,938 (9,030 to 13,256) male prevalent cases of BC in the NAME region. This number leads to 35,405 (30,676 to 40,571) deaths among female patients and 809 (654 to 1,002) deaths in male patients this year. BC was responsible for 1,222,835 (1,053,073 to 1,411,009) DALYs among female patients in 2019, with a greater proportion (94.9%) of burden in years of life lost (YLLs). The major contributor to female BC incidence increase in the past three decades was found to be increase in age-specific incidence rates of BC (227.5%), compared to population growth (73.8%) and aging (81.8%). The behavioral risk factors were responsible for majority of attributable female BC burden (DALYs: 106,026 [66,614 to 144,247]). High fasting plasma glucose was found to be the risk factor with the largest effect (DALYs: 84,912 [17,377 to 192,838]) on female BC burden.

**Conclusion:**

The increasing incidence and burden of BC in the NAME region is remarkable, especially when considering limited resources in the developing countries of this region. Proper policies like expanding screening programs and careful resource management are needed to effectively manage BC burden.

## Introduction

Breast cancer (BC) is the most prevalent neoplasm in female patients globally, with an increasing trend of incidence, almost in all regions (1, 2). BC is the leading cause of deaths due to cancer in female patients, and the mortality rate of this burdensome cancer has increased in most of the regions specifically in developing countries (3–5). In the most recent global cancer statistics updates (GLOBOCAN 2020), BC has overtaken lung cancer in first place to become the top cancer with the highest number of new cases (4, 6). BC contributes to about one-fourth of deaths due to malignancies in post-menopausal women, and female BC is the fifth leading cause of death due to cancer, following lung, colorectal, liver, and gastric cancers globally (4, 7). Although BC incidence is higher in developed countries, the relative mortality rate of this cancer is higher in developing countries (8). The increasing incidence of BC is suggested to happen due to improved BC screening tools like widespread use of mammography besides a substantial increase in exposure to various risk factors (4, 9, 10). So far, screening programs for BC in populations are designed and implemented to reduce BC death by early detection of malignancy and timely treatment interventions (11, 12).

The North Africa and Middle East (NAME) region is projected to have the greatest increase in all types of cancers in the next decades due to population aging and growth, compared to other World Health Organization (WHO) regions (13–15). In the period of 2005–2015, BC was the most common cancer in incident cases and the second leading cause of cancer deaths in this region (16). Among all cancers, BC has a significant importance when inspecting its epidemiology in the NAME region, due to various reasons. Cultural and socio-economic differences, the developing status of most of the countries located in this region, and multiple barriers to an efficient approach to handle the growing burden of BC are the leading issues that need to be considered in these countries (17–19). It is notable that most of the BC cases in this region are amenable to prevention and treatment; however, delayed diagnosis and treatment may explain the increase in burden of this cancer (20). On the other hand, management of BC in the NAME region faces several challenges, including younger age at presentation, aggressive behavior, lack of national breast screening programs, and lack of reliable data registries as well as socioeconomic factors; thus, these factors make it very challenging to apply the international guidelines for BC management in this region (10, 21, 22).

Besides the fragile status of female BC, male BC has a mysterious and poorly known epidemiology in the NAME region (23, 24). This poor knowledge is a global concern and is mainly due to scarcity of BC in men (25). BC in male patients is believed to be a distinct cancer from BC in female patients, due to different epidemiology, risk factors, prognosis, and profoundly varied molecular, biologic, and clinicopathologic features of this malignancy in men (25–28). Previous studies showed that male BC has a higher relative proportion of BC cases in the NAME region compared to the Western regions of world (24). Therefore, improvements in data shortages regarding male BC is necessary to pave the way for effective management and treatment of BC in men (29).

Epidemiologic data on the BC in the NAME region have many geographical variations with gaps in several areas (30, 31). In this paper, we present the major epidemiologic measures of BC and its risk factors in the NAME region and their trends during the 30-year period from 1990 to 2019 using the Global Burden of Disease (GBD) Study 2019. Additionally, epidemiology of risk factors of female BC in this region is presented in this study. A clear visualization of the BC status in this neglected region could help international, regional, and national health authorities to properly focus on this principal cancer and its huge burden in the NAME region. Therefore, we aimed to present the latest updated epidemiologic measures of BC and its responsible risk factors for the NAME region to provide the material for further actions and policies.

## Methods

### Data sources

This study is a part of the GBD Study 2019 provided by the Institute for Health Metrics and Evaluation (IHME), conducted for the NAME region, whose main data are accessible through its portal (32). GBD 2019 provides data of global, regional, and national burden of 369 diseases and injuries and 87 risk factors in 204 countries and territories. Also, subnational data of 21 countries are available in the GBD 2019. Detailed methods of GBD studies are provided in previous publications (33, 34). NAME is one of the seven super-regions in this study that encompasses 21 countries, namely, Afghanistan, Algeria, Bahrain, Egypt, Iran (Islamic Republic of), Iraq, Jordan, Kuwait, Lebanon, Libya, Morocco, Oman, Palestine, Qatar, Saudi Arabia, Sudan, Syrian Arab Republic, Tunisia, Turkey, United Arab Emirates, and Yemen. IHME uses a hierarchical leveling of causes into three main categories of communicable, maternal, neonatal, and nutritional diseases (CMNNDs); non-communicable diseases (NCDs); and injuries, in which neoplasms belong to the NCDs category. BC as the investigated cause in this study is defined as cause B.1.14 in the GBD cause list. More details on the mapped International Classification of Diseases and Injuries (ICD) codes of BC incidence and mortality to this cause in GBD are available in the **Supplementary Methods**. Also, diseases’ risk factors are categorized into three main categories of environmental and occupational risks, behavioral risks, and metabolic risks and follows a hierarchy of risks in GBD risk assessments (34). The other data source used to evaluate the cancer screening, registry, and action plans among the countries of the region of focus in this investigation was the WHO Global Health Observatory (GHO) database for 2019 (35). Moreover, the universal health coverage (UHC) based on an index of effective coverage of health services provided by GBD 2019 study was the other dataset used to rank and discuss the overall healthcare services and BC management state among the countries of the NAME region in this study (36). This study is conducted according to the Guidelines for Accurate and Transparent Health Estimates Reporting (GATHER) statement (37).

### Data estimation framework

The cornerstone of data estimations for GBD studies is mortality estimation made on the cause of death (CoD) database containing information from different sources, which are mainly cancer registries in the cancer data estimation, like BC in this study (**Supplementary Figures 1, 2**). The full list of data sources used to estimate BC data for the NAME region and countries in this study is provided in the **Supplementary Table 1**. In brief, cancer registries, including both incidence and mortality data of cancers, are used to obtain mortality to incidence (MIR) ratios in the estimation process. The three indices of mortality, incidence, and MIR are then aggregated and estimated by several calculations to provide the most precise information on cancers (33, 38). Risk factor estimations are made by the comparative risk assessment (CRA) conceptual framework that provides a hierarchy of risks and causes contributing to health outcomes. The effect size estimation in GBD is done by the relative risk (RR) modeling for level of exposure to risk or cause for mortality or morbidity (34) (**Supplementary Table 2**). The full description of the data estimation process for BC and attributable burden to its risk factors are provided in the **Supplementary Methods** (section A) of this paper.

### Study variables

To investigate epidemiology and burden of BC in the NAME region, a series of variables including incidence, prevalence, deaths, MIR, years of life lost (YLLs), years lived with disability (YLDs), and disability-adjusted life years (DALYs) were explored. Also, the measures were compared in different socio-demographic index (SDI) quintiles of included countries. Various risk factors for BC approved by IHME were included in two main categories of behavioral risk factors comprising alcohol use, diet high in red meat, low physical activity, smoking, and secondhand smoke, and metabolic risk factors comprising high body mass index (BMI) and high fasting plasma glucose (FPG) for female BC, and alcohol use, secondhand smoke, and diet high in red meat for BC in male patients (34). Deaths, YLLs, YLDs, and DALYs were explored for the investigated burden attributable to BC risk factors in this study. MIR and YLL/YLD ratio were estimated using age-standardized rates and utilized to compare the BC statistics between countries and sexes. Higher MIR values indicate higher fatality of condition and higher YLL/YLD values indicate higher mortality rather than morbidity for BC. Furthermore, we used the SDI and Healthcare Access and Quality (HAQ) Index to present results of this study. SDI is a composite index of income per capita, educational attainment, and fertility rates of geographic areas provided by IHME to compare diseases and risks based on socioeconomic factors (33). The estimated SDI values for the regions and countries included in this study could be found in **Supplementary Table 3**. The HAQ Index is a proxy of personal healthcare access and quality developed by IHME and is calculated by using MIRs for cancers and risk-standardized mortality rates for non-cancer causes (39). We used the recent estimated all-ages HAQ Index values for both sexes for the countries of the NAME region using the results of GBD study 2019 to compare BC measures, whose index values are found in **Supplementary Table 4**.

### Statistical analysis

Fourteen age groups (15–19, 20–24, …, 75–79, 80+) were used to investigate BC indices in this study. We chose this age range and groups based on the richness of estimated data of the GBD Study 2019 on the BC cause and the responsible risk factors. The number values were reported in all-ages scale and the rates were reported in age-standardized scale per 100,000 population. Age-standardized rates were estimated using the GBD age standard world population, updated to the 2019 standard population age structure (33). Percent changes of measures were calculated for the period 1990–2019. All estimations were made with a 95% uncertainty interval (UI) generated using the 25th and 975th ordered values in 1,000 draws of the posterior distribution. To evaluate the contribution of population growth, population aging (or age structure changes), and age-specific incidence rate changes on the BC incidence trend 1990–2019, we did a decomposition analysis on incidence in two scenarios. In the first scenario, we applied the age and sex structure and the age-specific rates of 1990 to the 2019 population. In the second scenario, we applied the age-specific rates of 1990 to the age and sex structure and population size of 2019. The difference between the total number of incident cases in 1990 and the first scenario was attributable to population growth. The difference between the second and first scenarios was attributable to population aging. The difference between the total number of incident cases in 2019 and the second scenario was attributable to age-specific rates changes (40). The full description of the steps of the decomposition analysis is found in **Supplementary Methods** (section B) of the **Supplementary Material**. Percentages of each of the three factors in a hypothetical assumption of two other factors being constant, and the overall change of BC incidence are provided for a better understanding of contributors to BC incidence growth through the past three decades. Data handling and visualizations in this study were done using STATA version 11 (STATA Corp., College Station, Texas, USA) and R 4.1.2 for Windows.

## Results

**Figure 1:**
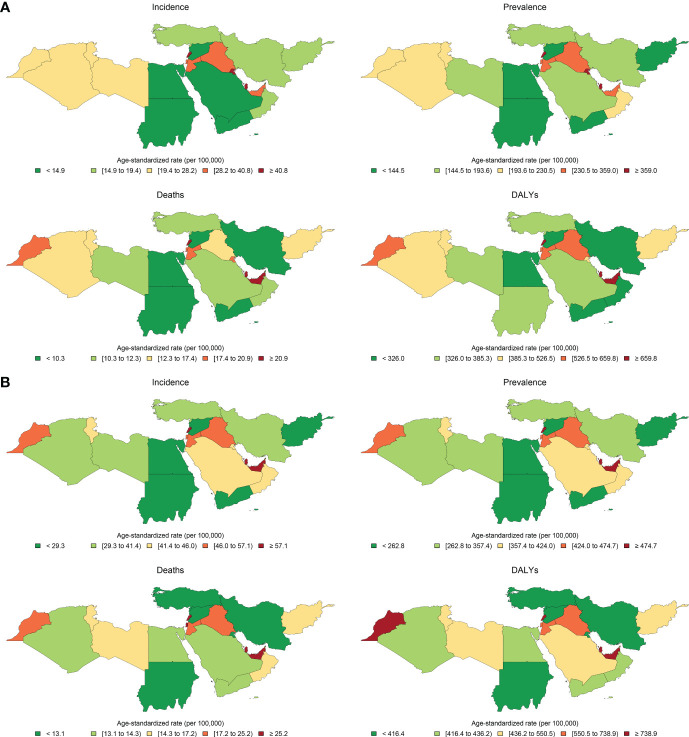
Age-standardized rates of incidence, prevalence, deaths, and disability-adjusted life years (DALYs) of breast cancer in female population in **(A)** 1990 and **(B)** 2019 in countries of the North Africa and Middle East region.

Various epidemiologic features of BC in the NAME region have different patterns in the last three decades (**Figure 1**, **Table 1**). Also, each country had distinct characteristics based on BC statistics (**Supplementary Table 5**). A brief notation of results is provided here and more are available in the appendices of this paper. The results provided in this manuscript focus on female and male patients by sex separately.

**Table 1:** Trend of epidemiologic indices of breast cancer in the North Africa and Middle East region, number for all ages and age-standardized rates, by sex, in 1990 and 2019 and percent of changes in the 1990–2019 period.

Measure	Metric	1990		2019		1990–2019 percent change (%)	
		Female	Male	Female	Male	Female	Male
**Incidence**	Number	19,610 (17,751 to 22,419)	517 (405 to 648)	94,746 (82,334 to 108,875)	1,444 (1,153 to 1,791)	383.2 (293.7 to 462.9)	179.1 (95.5 to 273.8)
	Rate	19.6 (17.8 to 22.6)	0.6 (0.5 to 0.8)	37.5 (32.7 to 42.9)	0.7 (0.5 to 0.8)	90.9 (54.6 to 122.1)	4.6 (−26.8 to 41.6)
**Prevalence**	Number	172,645 (156,716 to 196,577)	3,323 (2,687 to 4,029)	835,576 (741,968 to 944,851)	10,938 (9,030 to 13,256)	384 (310.8 to 458.7)	229.2 (142.2 to 321.6)
	Rate	177.6 (160.1 to 203.6)	3.8 (3.1 to 4.6)	332.7 (297.2 to 374.9)	4.8 (4 to 5.8)	87.3 (59.4 to 114.8)	27.1 (−6.5 to 63.4)
**Deaths**	Number	11,524 (10,398 to 13,312)	400 (310 to 508)	35,405 (30,676 to 40,571)	809 (654 to 1,002)	207.2 (147.6 to 261)	102.3 (43.6 to 170)
	Rate	12.3 (11 to 14.2)	0.5 (0.4 to 0.7)	15.2 (13.3 to 17.3)	0.4 (0.3 to 0.5)	24 (−0.8 to 45.6)	−23.8 (−46.2 to 3.1)
**YLLs (years of life lost)**	Number	395,401 (358,345 to 454,026)	10,223 (7,926 to 12,858)	1,160,511 (998,083 to 1,346,371)	20,358 (16,379 to 25,142)	193.5 (139 to 243.4)	99.1 (41.7 to 165.2)
	Rate	382.7 (345.9 to 440.6)	11.3 (8.8 to 14.2)	448.2 (386.3 to 516.6)	8.6 (7 to 10.6)	17.1 (−5.3 to 37.4)	−23.6 (−45.9 to 1.3)
**YLDs (years lived with disability)**	Number	12,685 (8,831 to 17,508)	325 (210 to 457)	62,324 (43,715 to 85,058)	1,016 (676 to 1,424)	391.3 (301.6 to 472.4)	212.6 (122.6 to 317.4)
	Rate	12.7 (8.8 to 17.5)	0.4 (0.2 to 0.5)	24.5 (17.2 to 33.5)	0.5 (0.3 to 0.6)	93 (58.6 to 125)	19.3 (−14.9 to 60.1)
**DALYs (disability-adjusted life years)**	Number	408,086 (370,190 to 472,046)	10,548 (8,203 to 13,267)	1,222,835 (1,053,073 to 1,411,009)	21,374 (17,235 to 26,386)	199.7 (143.9 to 251.3)	102.6 (43.9 to 170.4)
	Rate	395.4 (357.6 to 458.5)	11.6 (9.1 to 14.7)	472.7 (409 to 544.8)	9.1 (7.3 to 11.2)	19.5 (−3.2 to 40.5)	−22.2 (−44.8 to 3)

Data in parentheses are 95% uncertainty intervals.

### Incidence

The all-ages number of BC among female patients of NAME region was estimated to be 19,610 (95% UI: 17,751 to 22,419) in 1990 and 94,746 (82,334 to 108,875) in 2019, reflecting a 383.2% (293.7 to 462.9) increase. The overall age-standardized rates of BC incidence per 100,000 among female patients in this region was estimated to be 37.5 (32.7 to 42.9) in 2019 compared to 19.6 (17.8 to 22.6) in 1990, indicating an almost two times (90.9% [54.6 to 122.1]) increase in the comparison of rates. Investigating the incidence rates among countries located in this region, we found Lebanon (122.5 [92.1 to 160.7]) to have the highest female BC rate and Afghanistan (22.3 [16.8 to 29.1]) to have the lowest rate. Age-standardized incidence rate was most prominently increased in Saudi Arabia (189.8% [79.0 to 358.9]), Lebanon (152.9% [76.0 to 255.6]), and Oman (131.5% [45.4 to 263.8]), in contrast to Kuwait (3.4% [−18.3 to 36.7]), Afghanistan (30.7% [−8.5 to 79.5]), and United Arab Emirates (41.0% [−6.9 to 112.1]) with the lowest rates of increase in terms of percent change. Considering age groups, BC is first detected in female patients in their 20s and accelerates almost consistently by advancing age (**Supplementary Figure 3**).

Investigating BC among male patients, the all-ages number of incident cases in 2019 were approximately 1,444 (1,153 to 1,791) with a 179.1% (95.5 to 273.8) increase compared to this measure in 1990 (517 [405 to 648]). However, it is important to mention that the age-standardized incidence rate of BC among male patients living in the NAME region changed slightly (4.6% [−26.8 to 41.6]) during the 30 years of this study (0.6 [0.5 to 0.8] per 100,000 in 1990 and 0.7 [0.5 to 0.8] in 2019). Comparing the recent incidence rates of male BC among countries of this region in 2019, we found Egypt (0.1 [0.06 to 0.15]) to have the lowest rate and Algeria (2.7 [1.7 to 3.9]) to have the highest rate in this regard. The trend of male BC incidence rates in terms of percent change in this period showed Bahrain (134.8% [27.0 to 306.4]), Iraq (92.2% [15.4 to 218.8]), and Syrian Arab Republic (60.6% [0.2 to 159.4]) having the highest increase, and on the other side, Algeria (−33.8% [−60.0 to 5.6]), Afghanistan (−27.0% [−53.9 to 26.1]), and Yemen (−20.1% [−51.1 to 46.3]) had the highest reductions in this measure.

Based on the decomposition analysis of incidence rate change of BC in female patients in the NAME region, it is evident that in 2019, a total of 75,136 more cases of BC has been found compared to 1990, representing a 383.2% overall increase, of which 73.8% was estimated to be attributable to population growth, 81.8% was attributable to population aging, and 227.5% was attributable to change of incidence rates of BC. The highest detected overall increase of incidence was in the United Arab Emirates, Qatar, and Saudi Arabia, respectively, and in all these three countries, the true increase of BC incidence was the prominent contributor in the decomposition analysis. Also, of the 179.1% increase of BC in male patients, 78.9% was attributable to population growth, 81.9% was attributable to population aging, and 18.3% was attributable to change of BC incidence rates (**Table 2**).

**Table 2:** Decomposition analysis of breast cancer incidence trend 1990–2019, by sex, in the North Africa and Middle East region and countries.

Location	Sex	Number of incidences		Expected number of incidences in 2019		Causes of incidences change trend 1990-2019			Overall change 1990-2019
		1990	2019	Population growth	Population growth and aging	Population growth	Population aging	Incidence rate change	
**North Africa and Middle East**	Female	19,610	94,746	34,085	50,135	73.8%	81.8%	227.5%	383.2%
	Male	517	1,444	926	1,349	78.9%	81.9%	18.3%	179.1%
**Afghanistan**	Female	644	1,951	2,079	1,541	223.0%	−83.5%	63.7%	203.2%
	Male	28	37	97	49	247.7%	−171.1%	−44.8%	31.8%
**Algeria**	Female	1,526	6,621	2,521	4,318	65.2%	117.8%	150.9%	334.0%
	Male	195	430	324	597	65.8%	140.2%	−85.6%	120.4%
**Bahrain**	Female	47	346	120	247	155.2%	271.9%	210.6%	637.7%
	Male	0	3	1	1	204.8%	303.1%	623.1%	1,131.0%
**Egypt**	Female	2,509	10,600	4,403	5,251	75.5%	33.8%	213.1%	322.4%
	Male	10	37	18	23	80.2%	49.9%	142.1%	272.2%
**Iran (Islamic Republic of)**	Female	2,835	14,743	4,110	8,050	44.9%	139.0%	236.0%	420.0%
	Male	36	120	51	93	43.1%	117.3%	73.8%	234.2%
**Iraq**	Female	1,337	7,819	3,189	4,363	138.4%	87.8%	258.4%	484.6%
	Male	13	83	32	40	140.3%	60.7%	317.8%	518.8%
**Jordan**	Female	301	2,053	909	1,444	201.6%	177.5%	202.3%	581.4%
	Male	4	22	14	22	214.6%	204.5%	−12.0%	407.2%
**Kuwait**	Female	145	716	397	752	173.6%	244.7%	−24.9%	393.4%
	Male	2	8	4	7	135.2%	166.3%	19.5%	321.0%
**Lebanon**	Female	613	3,519	995	1,395	62.2%	65.3%	346.6%	474.2%
	Male	10	34	16	23	54.0%	72.6%	108.8%	235.4%
**Libya**	Female	209	1,347	337	681	61.1%	164.2%	317.8%	543.2%
	Male	7	19	10	17	57.0%	107.6%	20.7%	185.3%
**Morocco**	Female	2,348	9,755	3,307	5,239	40.9%	82.3%	192.3%	315.5%
	Male	44	137	63	101	43.4%	85.8%	82.0%	211.2%
**Oman**	Female	68	427	136	195	101.3%	86.1%	343.3%	530.6%
	Male	7	21	19	22	160.6%	41.7%	−12.9%	189.4%
**Palestine**	Female	176	840	417	509	136.7%	52.1%	187.5%	376.3%
	Male	1	5	3	4	142.1%	49.8%	157.0%	348.8%
**Qatar**	Female	30	382	147	226	392.2%	265.6%	525.6%	1,183.4%
	Male	1	6	5	7	618.7%	310.0%	−108.5%	820.2%
**Saudi Arabia**	Female	464	5,330	976	1,772	110.2%	171.5%	766.4%	1,048.2%
	Male	11	41	26	35	132.5%	87.0%	47.4%	267.0%
**Sudan**	Female	730	2,846	1,469	1,616	101.3%	20.3%	168.5%	290.1%
	Male	27	56	54	57	102.8%	9.1%	−1.6%	110.2%
**Syrian Arab Republic**	Female	422	1,873	498	980	18.0%	114.1%	211.5%	343.6%
	Male	3	9	3	5	7.0%	102.8%	128.1%	237.9%
**Tunisia**	Female	680	3,129	950	1,694	39.6%	109.4%	210.9%	359.9%
	Male	15	43	20	35	34.7%	103.7%	59.6%	198.0%
**Turkey**	Female	4,047	17,130	5,529	9,266	36.6%	92.3%	194.3%	323.3%
	Male	86	250	117	209	35.6%	106.9%	48.3%	190.8%
**United Arab Emirates**	Female	86	1,220	331	773	282.9%	510.9%	517.1%	1,310.9%
	Male	4	51	23	45	454.1%	516.3%	153.8%	1,124.1%
**Yemen**	Female	378	2,001	875	1,094	131.2%	57.9%	239.8%	429.0%
	Male	13	31	30	37	127.7%	53.1%	−47.3%	133.6%

### Prevalence

The all-ages prevalence of BC among female patients in the NAME region was 172,645 (156,716 to 196,577) in 1990 and 835,576 (741,968 to 944,851) in 2019, showing a 384% (310.8 to 458.7) increase. The age-standardized prevalence of female BC has increased steadily from 177.6 (160.1 to 203.6) per 100,000 female individuals in 1990 to 332.7 (297.2 to 374.9) in 2019 with an 87.3% (59.4 to 114.8) increase. Among countries of this region in 2019, Lebanon (1,083.5 [844.6 to 1,383.5]) ranked first in comparison of prevalence rates with a noticeable difference with the second-ranked country, Qatar (880.3 [701.3 to 1,098.3]), while Afghanistan (153.1 [121.8 to 192.8]) ranked the last in this measure. The rising prevalence of BC among female patients has been detected in all the nations of this region with no exception. Lebanon (161.6% [90.9 to 252.8]), Saudi Arabia (155.7% [77.4 to 267.6]), and Oman (113.3% [54.3 to 191.9]) had the highest increase in the BC prevalence rates among female patients, while Kuwait (12.5% [−6.9 to 39.7]), Afghanistan (22.5% [−6.9 to 58.9]), and United Arab Emirates (44.6% [5.0 to 96.5]) had the lowest changes in this regard.

The BC prevalence in male patients of this region estimated approximately 10,938 (9,030 to 13,256) cases in 2019 and 3,323 (2,687 to 4,029) in 1990, exhibiting a 229.2% (142.2 to 321.6) increase during the study period. However, the age-standardized rates of BC in male patients increased more slightly (27.1% [−6.5 to 63.4]) in NAME and reached 4.8 (4 to 5.8) cases per 100,000 in 2019. Algeria (18.1 [12.2 to 25.6]), Oman (18.1 [12.9 to 25.1]), and Lebanon (10.5 [7.4 to 14.9]) had the highest prevalence rates of BC in male patients in 2019 contrary to Egypt (0.9 [0.6 to 1.2]), Syrian Arab Republic (1.1 [0.9 to 1.5]), and Iran (2.8 [2.3 to 3.3]). The estimated change in BC prevalence rates among male patients during the study period was highest in Iraq (116.8% [39.2 to 236.7]), Bahrain (105.5% [32.4 to 214.5]), and Lebanon (75.0% [7.0 to 184.9]), while Afghanistan (−14.7% [−43.2 to 32.7]), Algeria (−9.1% [−42.6 to 41.0]), and Yemen (−5.5% [−38.3 to 54.1]) had a negative change in this regard.

### Deaths

**Figure 2:**
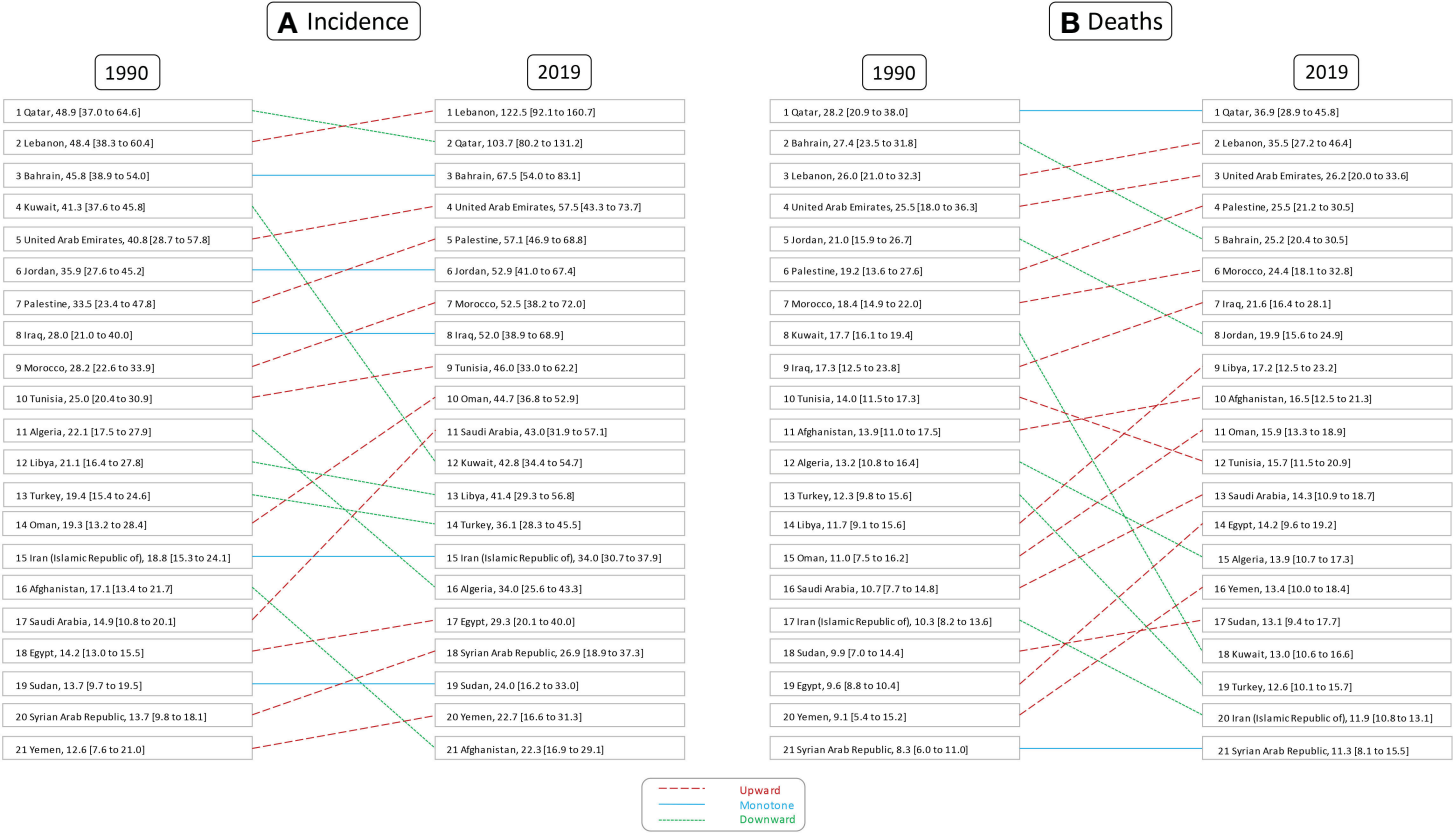
Rank of age-standardized rates of female breast cancer **(A)** incidence and **(B)** deaths in countries of the North Africa and Middle East region, in 1990 and 2019.

Altogether, there were 35,405 (30,676 to 40,571) deaths among female patients in 2019 in the NAME region, accounting for 15.2 (13.3 to 17.4) age-standardized deaths in 100,000 individuals and 2.6% (2.4 to 2.8) of all-cause female patients’ deaths. In comparison to 1990, 11,524 (10,398 to 13,312) deaths are estimated for NAME, accounting for 12.3 (11.0 to 14.2) age-standardized deaths and 1.0% (0.1 to 1.1) of their total deaths. Both the percent change in all-age deaths number (207.2% [147.6 to 261]) and the death rates (24.0% [−0.8 to 45.6]) were increased in female patients with BC during the study period. Investigating countries showed Qatar (36.9 [28.9 to 45.8]), Lebanon (35.5 [27.2 to 46.4]), and United Arab Emirates (26.2 [20.0 to 33.6]) having the highest death rates in 2019, in contrast to Syrian Arab Republic (11.3 [8.1 to 15.5]), Iran (11.9 [10.8 to 13.1]), and Turkey (12.7 [10.1 to 15.7]). Exploring changes in age-standardized rates found Egypt (49.0% [−2.0 to 106.6]), Yemen (46.9% [−6.7 to 159.1]), and Libya (46.7% [−8.3 to 126.5]) with the highest growth, while Kuwait (−26.7% [−41.3 to −4.2]), Bahrain (−8.1% [−28.5 to 16.6]), and Jordan (−5.1% [−31.0 to 33.8]) had the best improvements in death rates (**Figure 2**).

In 2019 in the NAME region, approximately 809 (654 to 1,002) deaths were estimated to happen due to BC in male patients, which is double that of 1990 (400 [310 to 508]). Although the percent change in all-age deaths number was positive (102.3% [43.6 to 170]) in the study period, the death rates had a negative (−23.8% [−46.2 to 3.1]) change in male patients with BC. Investigating death rates among countries of this region, we found Algeria (1.7 [1.1 to 2.5]), Oman (1.4 [1.0 to 2.1]), and Lebanon (0.7 [0.5 to 1.1]) to have the highest deaths, as compared to Egypt (0.06 [0.04 to 0.09]), Syrian Arab Republic (0.07 [0.05 to 0.1]), and Iran (0.16 [0.13 to 0.17]) with the lowest death rates in male patients with BC. Percent changes in death rates due to BC in male patients showed Bahrain (63.1% [−12.3 to 176.1]), Iraq (41.1% [−15.0 to 127.2]), and Palestine (31.0% [−18.4 to 125.6]) having the greatest increases, in contrast to Algeria (−52.2% [−70.9 to −19.2]), Jordan (−36.8% [−64.9 to 5.2]), and Afghanistan (−32.4% [−57.5 to 15.7]) with most improvements in this regard.

### DALYs, YLLs, and YLDs

**Figure 3:**
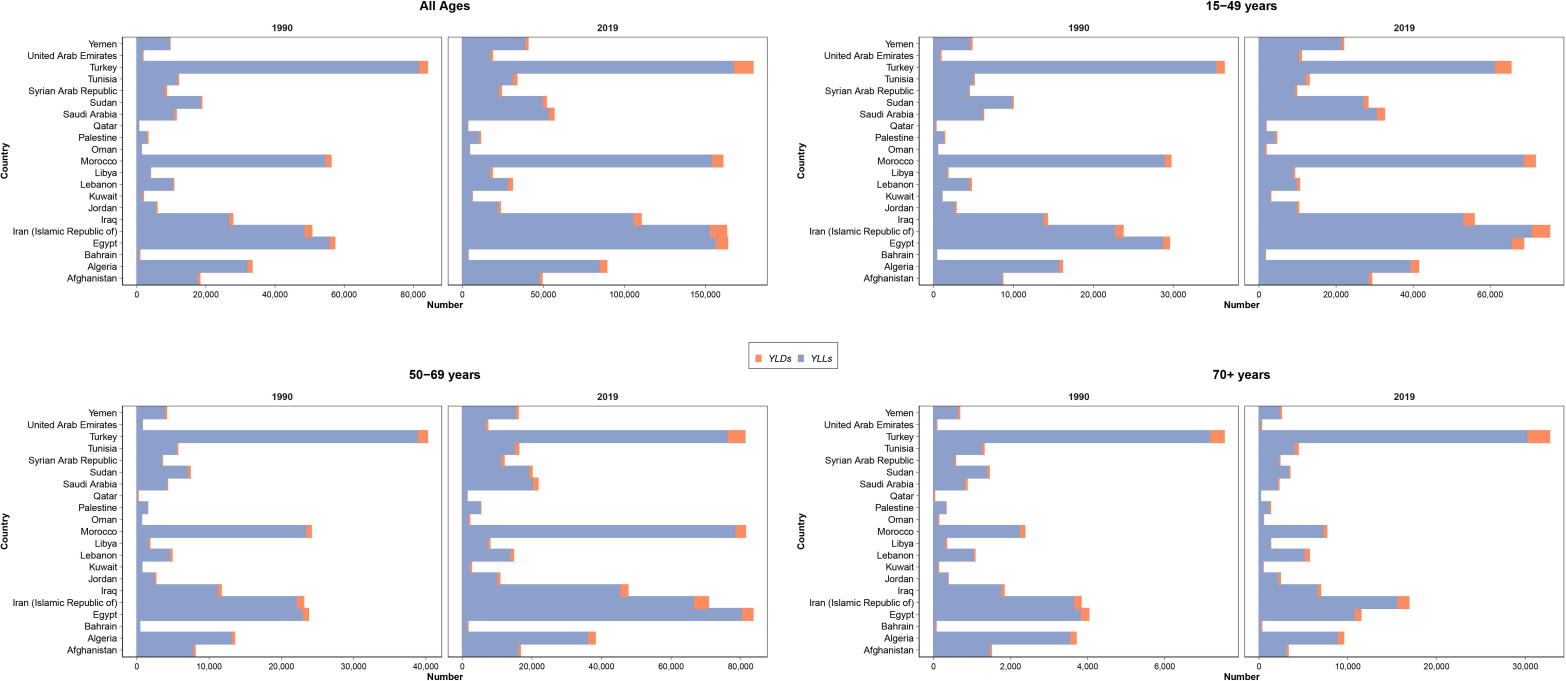
Distribution of years lived with disability (YLDs) and years of life lost (YLLs) due to female breast cancer in all ages, 15–49, 50–69, and 70+ age categories, in countries of the North Africa and Middle East region.

The 2019 GBD estimates revealed that BC was responsible for 1,222,835 (1,053,073 to 1,411,009) DALYs among female patients, which was composed of 1,160,511 (998,083 to 1,346,371) YLLs and 62,324 (43,715 to 85,058) YLDs. The all-ages number of DALYs, YLLs, and YLDs due to BC in female patients had a 199.7% (143.9 to 251.3), 193.5% (139 to 243.4), and 391.3% (301.6 to 472.4) increase during the study period. Among countries located in this region, Lebanon (1,067 [809 to 1,407]), Qatar (856 [663 to 1,075]), and Morocco (843 [612 to 1,158]) had the highest BC burden in terms of age-standardized DALY rates in 2019, while Syrian Arab Republic (334 [237 to 472]), Kuwait (359 [290 to 462]), and Iran (369 [337 to 404]) had the lowest rates (**Figure 3**).

The BC in male patients of this region caused 21,374 (17,235 to 26,386) DALYs in 2019, which was an aggregation of 20,358 (16,379 to 25,142) YLLs and 1,016 (676 to 1,424) YLDs. The all-ages DALYs, YLLs, and YLDs of BC in male patients had a 102.6% (43.9 to 170.4), 99.1% (41.7 to 165.2), and 212.6% (122.6 to 317.4) increase, respectively. Although the age-standardized rates of DALYs (−22.2% [−44.8 to 3]) and YLLs (−23.6% [−45.9 to 1.3]) had a decreasing pattern during the study period, the YLD rates (19.3% [−14.9 to 60.1]) were increasing. DALY rates due to BC in male patients in 2019 were highest in Algeria (34.6 [22.5 to 48.9]), Oman (26.3 [17.7 to 37.0]), and United Arab Emirates (17.1 [8.1 to 30.7]), in contrast to Egypt (1.7 [1.0 to 2.4]), Syrian Arab Republic (1.9 [1.3 to 2.6]), and Iran (4.0 [3.5 to 4.5]).

### MIR and YLL/YLD

MIR in female patients with BC was estimated to be 0.41 in 2019 and 0.63 in 1990 in the NAME region, reflecting a 35.05% decrease in this index. The countries with the highest MIR in female patients with BC in 2019 were Afghanistan (0.74), Yemen (0.59), and Sudan (0.55) compared to Lebanon (0.29), Kuwait (0.30), and Saudi Arabia (0.33) with the lowest estimated MIRs. This index was estimated to be approximately 0.84 in 1990 and 0.61 in 2019 among male patients with BC in this region, which showed a 27.2% decrease. Male patients with BC in Afghanistan (0.89), Yemen (0.79), and Sudan (0.76) had the worst state regarding MIR in 2019, while individuals in Kuwait (0.48), Lebanon (0.49), and Iran (0.50) had the lowest MIR. Dividing the MIR values of male to female patients in the NAME region showed that in both 1990 (1.3) and 2019 (1.5), the prognosis of BC is much poorer in male patients compared to female patients and this cancer takes more lives in the affected male patients.

**Figure 4:**
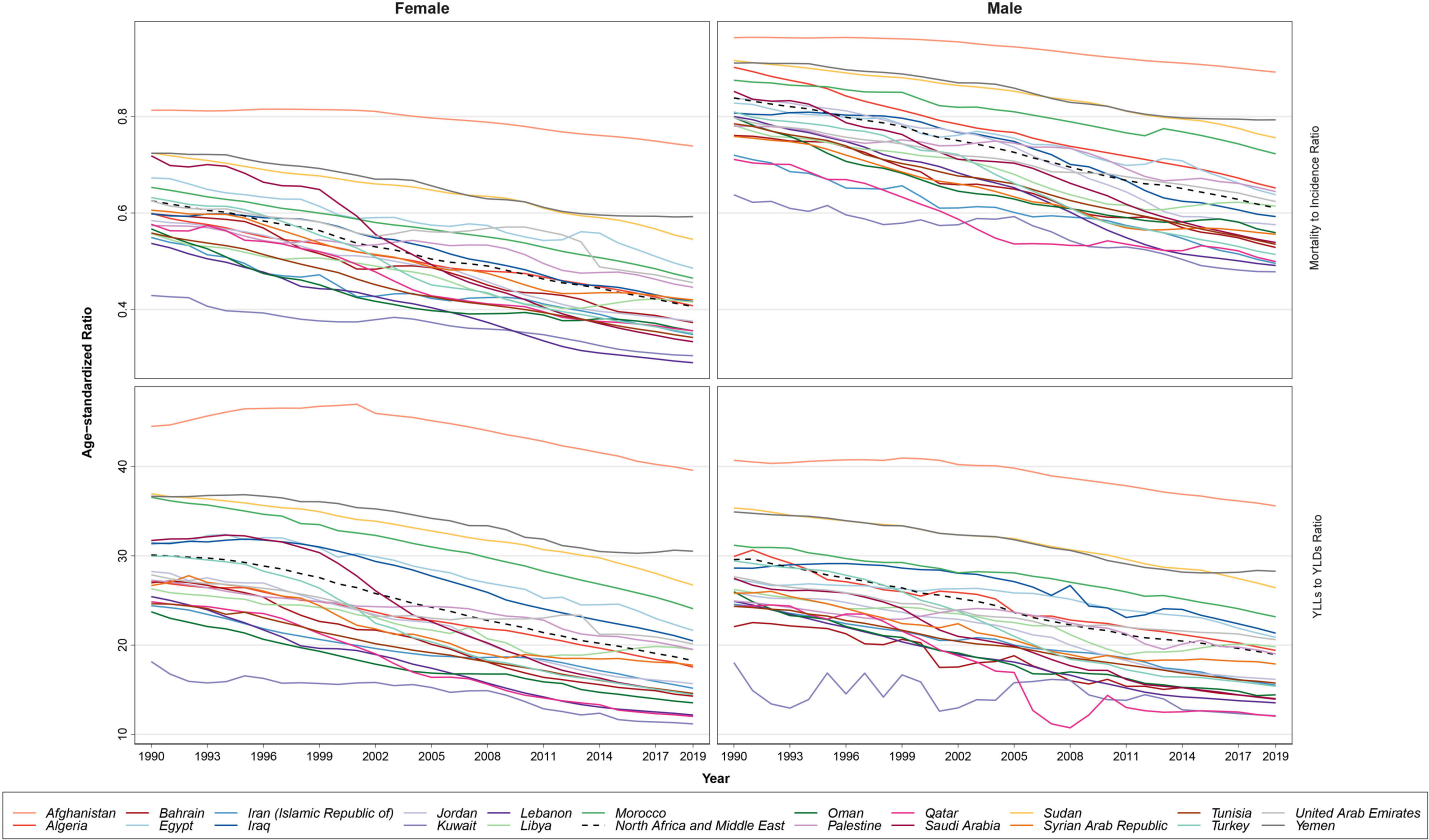
The patterns of the mortality to incidence ratio (MIR) and years of life lost (YLLs)/years lived with disability (YLDs) ratio trends based on age-standardized rates by sex, during the study period.

The YLL/YLD ratio for BC in female patients was estimated to be 30.1 in 1990 and 18.3 in 2019, with a 39.3% decrease in this index. The countries with the highest age-standardized YLL/YLD ratio for BC in female patients in 2019 were Afghanistan (39.6), Yemen (30.5), and Sudan (26.7), and the countries with the lowest values for this index were Kuwait (11.2), Qatar (12.0), and Lebanon (12.2). This index was calculated for BC in male patients to be approximately 29.6 in 1990 and 18.9 in 2019, which had a 36% decrease. Among the countries of this region, Afghanistan (35.6), Yemen (28.3), and Sudan (26.4) earned the highest YLL/YLD ratios in 2019 against Qatar (12.0), Kuwait (12.1), and Lebanon (13.5). Comparison of this ratio between sexes showed almost a similar pattern in both 1990 (male/female ratio: 0.98) and 2109 (male/female ratio: 1.04). Both of the examined ratios had a similar decreasing pattern in the region and included countries (**Figure 4**).

### SDI and HAQ Index

**Figure 5:**
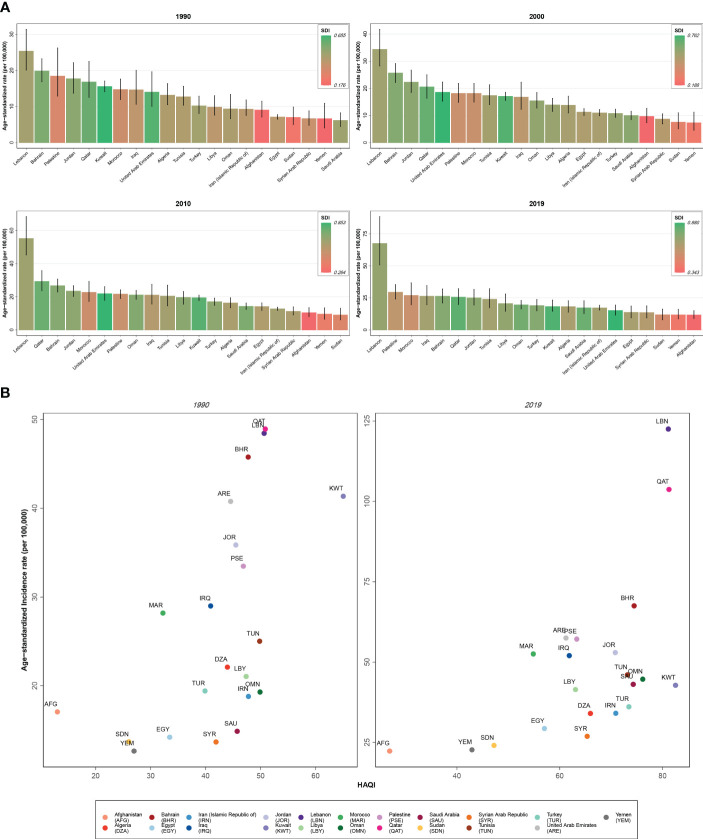
**(A)** Age-standardized rate of incidence of female breast cancer among countries of the North Africa and Middle East region compared by the socio-demographic index (SDI). **(B)** Age-standardized rate of incidence of female breast cancer among countries of the North Africa and Middle East region compared by the Healthcare Access and Quality (HAQ) Index.

Investigating BC incidence and prevalence rates among female patients in 2019, almost all nations with a higher SDI and HAQ Index showed higher incidence and prevalence rates (Lebanon, Qatar, Bahrain) and the countries with the lowest rates belonged to lower SDI categories and were those with lower HAQ Index values (Afghanistan, Yemen, Sudan). Also, the top three countries in death rates of BC in female patients had middle to higher SDI (Qatar, Lebanon, and United Arab Emirates) and Qatar and Lebanon also had the highest HAQ Index in the region following Kuwait. However, the pattern of BC DALYs in this comparison had no clear pattern. Regarding MIR and YLL/YLD of BC in female patients in 2019, countries with higher ratios all had the lowest SDI and HAQ Index (Afghanistan, Yemen, and Sudan) and countries with a lower MIR (Lebanon, Kuwait, Saudi Arabia) and YLL/YLD (Kuwait, Qatar, Lebanon) nearly all had high SDI and HAQ Index. Exploring the abovementioned measures for BC in male patients of this region in 2019, we found the pattern only for MIR and YLL/YLD (higher in low SDI and HAQ Index countries—Afghanistan, Yemen, and Sudan—and lower in countries with better SDI and HAQ Index) (**Figure 5**).

### Risk factor measures

**Figure 6:**
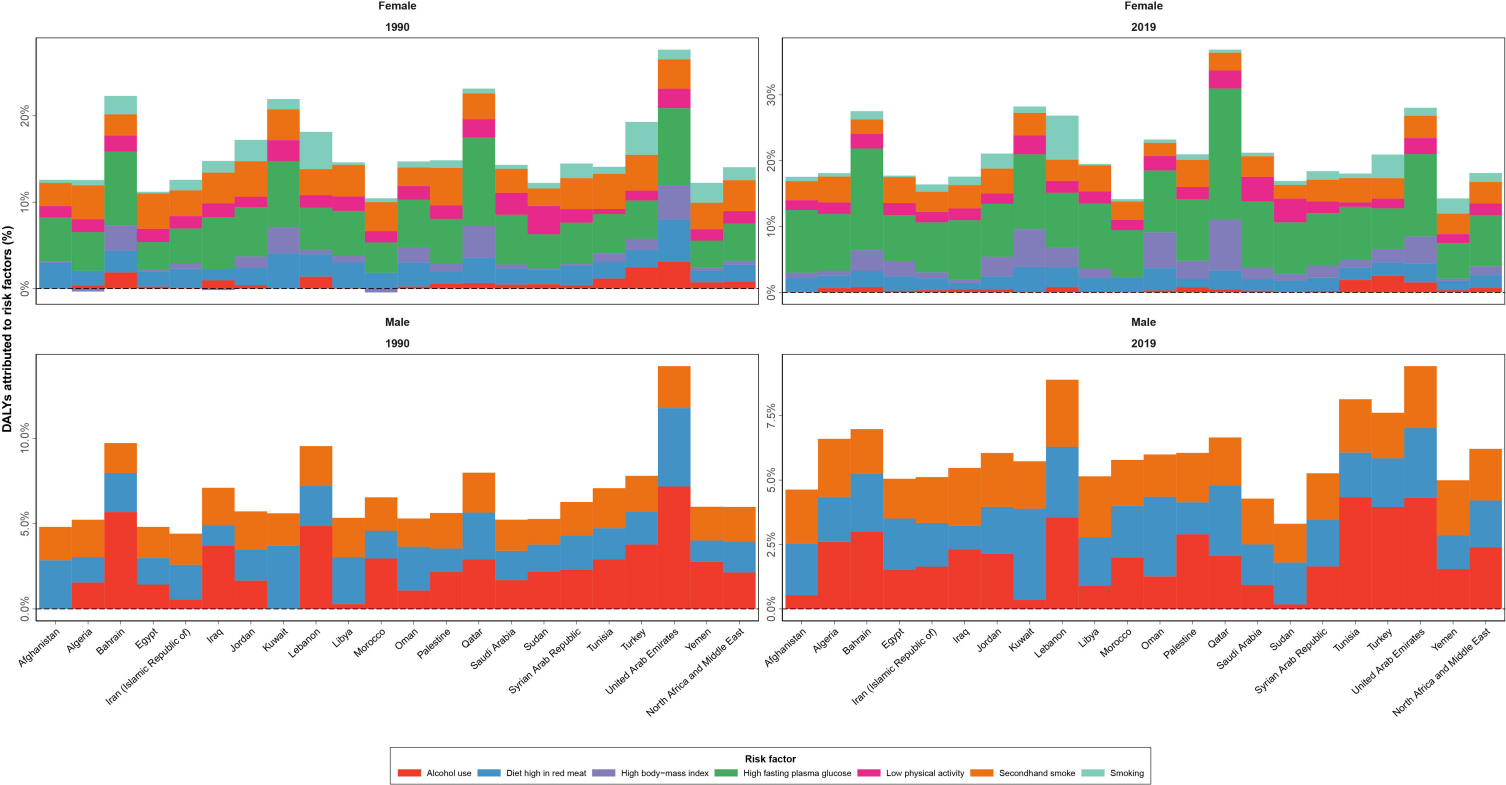
Disability-adjusted life years (DALYs) of breast cancer attributed to various risk factors contributing to this cancer by sex, in 1990 and 2019, in countries of the North Africa and Middle East region.

The all-ages deaths attributable to the risk factors of female BC were estimated to be 6,507 (3,576 to 10,469) in 2019 in the NAME region with a 290.3% (198.8 to 387.9) increase compared to 1990 (1,667 [959 to 249]). In total, 171,616 (80,804 to 297,481) DALYs were attributable to all seven BC risk factors in 2019 among female patients of the NAME region, with behavioral risk factors (firsthand and secondhand smoking, alcohol use, low physical activity, and diet high in red meat) being responsible for 106,026 (66,614 to 144,247) DALYs, followed by metabolic risk factors (high FPG and high BMI) being responsible for 72,729 (−22,214 to 192,180) DALYs. In 1990, 46,992 (24,371 to 72,765) DALYs were attributable to all these risk factors, indicating a 265.2% (169.9 to 361.8) change. High FPG was the risk factor with the largest effect (84,912 [17,377 to 192,838] DALYs), followed by secondhand smoking (41,012 [9,770 to 72,205]) and diet high in red meat (24,129 [5,414 to 34,461]). The protective effects of high BMI in relation to BC burden in DALYs (−7,656 [−66,973 to 36,339]) was found. The negative estimated DALYs were mainly due to a high value of YLLs in the premenopausal women (**Table 3**). The age-standardized DALY rates attributed to all BC risk factors in female patients were found to be the highest in Qatar (270.8 [137.2 to 443.3]), Lebanon (250.3 [150.3 to 390.9]), and United Arab Emirates (193.2 [100.3 to 320.5]), while being smallest in Iran (55.5 [27.5 to 91.0]), Syrian Arab Republic (55.8 [27.7 to 97.7]), and Yemen (57.7 [29.5 to 99.4]), in 2019. The countries with the highest growth of attributable DALY rates to BC risk factors in female patients during the study period were Egypt (116.0% [31.2 to 232.8]), Oman (102.0% [24.1 to 231.6]), and Libya (89.4% [5.9 to 206.0]), while Kuwait had a decreasing pattern (−14.5% [−34.2 to 15.5]), and Bahrain (2.4% [−28.1 to 38.4]) and Turkey (3.0% [−30.8 to 44.6]) were countries with the lowest growth in this regard (**Figure 6**, **Supplementary Tables 6, 7**).

**Table 3:** The all-ages numbers of deaths and disability-adjusted life years attributed to various breast cancer risk factors for female population, in the North Africa and Middle East region in 1990 and 2019 and the percentages of change in this period.

Risk factor	Measure	Year		% Change (1990 to 2019)
		1990	2019	
**All risk factors**	Deaths	1,667 (959 to 2494)	6,507 (3,576 to 10,469)	290.3 (198.8 to 387.9)
	DALYs	46,992 (24,371 to 72,765)	171,616 (80,804 to 297,481)	265.2 (169.9 to 361.8)
** Behavioral risks**	Deaths	1,037 (687 to 1,369)	3,115 (1,998 to 4,205)	200.5 (139.0 to 257.0)
	DALYs	36,169 (23,651 to 47,772)	106,026 (66,614 to 144,247)	193.1 (135.4 to 247.3)
Alcohol use	Deaths	89 (68 to 117)	236 (173 to 308)	164.3 (98 to 243.7)
	DALYs	3,374 (2,554 to 4,483)	8,601 (6,291 to 11,282)	154.9 (90.4 to 232.5)
Diet high in red meat	Deaths	222 (49 to 313)	677 (152 to 964)	205 (146.7 to 260.2)
	DALYs	8,076 (1,796 to 11,242)	24,129 (5,414 to 34,461)	198.8 (144.3 to 251.9)
Low physical activity	Deaths	183 (75 to 326)	680 (272 to 1,201)	272.6 (184.2 to 354.5)
	DALYs	5,526 (2,348 to 10,191)	20,324 (7,940 to 36,490)	267.8 (181.3 to 350.7)
Smoking	Deaths	179 (118 to 253)	499 (337 to 682)	179.2 (113.5 to 247.2)
	DALYs	5,827 (3,678 to 8,384)	15,634 (10,009 to 21,774)	168.3 (107.6 to 233.3)
Secondhand smoke	Deaths	402 (95 to 704)	1,133 (270 to 1,992)	181.5 (126.6 to 233.6)
	DALYs	14,676 (3,457 to 25,719)	41,012 (9,770 to 72,205)	179.4 (127.4 to 229.5)
** Metabolic risks**	Deaths	700 (70 to 1,544)	3,741 (758 to 7,700)	434.7 (282.1 to 794.2)
	DALYs	12,157 (−8,114 to 35,969)	72,729 (−22,214 to 192,180)	498.2 (−983.6 to 1,694.6)
High body mass index	Deaths	183 (−196 to 595)	987 (−603 to 2,667)	438.8 (−614.7 to 1,724.7)
	DALYs	−3,030 (−16,773 to 7,892)	−7,656 (−66,973 to 36,339)	152.7 (−1,423.7 to 1,875.4)
High fasting plasma glucose	Deaths	546 (98 to 1,274)	2,976 (604 to 6,690)	445.3 (333 to 569.6)
	DALYs	15,797 (2,804 to 37,471)	84,912 (17,377 to 192,838)	437.5 (330.3 to 554)

Data in parentheses are 95% uncertainty intervals. DALYs, disability-adjusted life years.

The responsible risk factors for BC in male patients in GBD 2019 only included alcohol use, secondhand smoke, and diet high in red meat. The all-ages DALYs and deaths attributable to these risk factors were estimated to be approximately 1,359 (885 to 1871) and 48 (30 to 68) in 2019 in the NAME region. The attributable DALYs to alcohol use was the highest (573 [418 to 769]), followed by secondhand smoke (415 [95 to 742]), and diet high in red meat (401 [88 to 610]). The countries with the highest attributable DALY rates for BC in male patients’ risk factors in 2019 were Algeria (2.2 [1.3 to 3.5]), United Arab Emirates (1.6 [0.7 to 3.1]), and Oman (1.6 [0.8 to 2.4]), while Egypt (0.08 [0.04 to 0.14]), Syrian Arab Republic (0.1 [0.05 to 0.15]), and Saudi Arabia (0.2 [0.1 to 0.3]) had the lowest rates. However, regarding the change of attributable DALY rates change during the study period, Palestine (33.9% [−20.5 to 133.4]), Egypt (30.0% [−25.1 to 112.8]), and Iraq (19.8% [−30.6 to 110.8]) had the most immense increase, while Sudan (−49.3% [−75.3 to −1.1]), United Arab Emirates (−47.2% [−75.3 to 1.6]), and Qatar (−46.2% [−72.5 to 5.0]) had the highest decreases in this measure.

## Discussion

The major findings of this study on BC status in the female patients of the NAME region during 1990 to 2019 were the almost doubling of incidence and prevalence rates and the three times increased death and YLLs rates caused by BC. Increase in the age-specific incidence rates was the main contributor to the increasing incidence of BC rather than population growth and aging. Behavioral risk factors were recognized as the greatest burden of female BC among all risk factors. However, high FPG as a metabolic risk factor was found to have the largest impact in burden of BC among women inspecting each risk factor. We found BC epidemiology to be alarming in Lebanon, Qatar, and Bahrain in terms of incidence and in Afghanistan, Yemen, and Sudan in terms of higher lethality and worst care given to patients with BC.

This study showed increasing trends of incidence and deaths due to BC in the NAME region, in both female and male patients. Changes in incidence and deaths of BC in the past decades are attributed to various causes. The increasing trend of BC incidence is thought to be due to expanded life expectancies resulting from improvement in healthcare systems, increased age of first pregnancy, earlier menarche, decreasing prevalence of breast feeding as a protective factor for BC, and increased exposure to BC risk factors like increased smoking, alcohol use, physical inactivity, obesity, and unhealthy dietary habits (10, 41–44). Moreover, improvement in BC screening and diagnosis by implementation of cancer guidelines and better and widespread use of breast imaging techniques, added to the increased knowledge about BC and early screening programs, leads to greater diagnosis of this cancer (7).

The current study found higher incidence and prevalence of BC in countries with higher SDI and HAQ index and vice versa. This major finding was consistent with previous studies that showed that socio-demographic and development status of countries have a substantial impact on BC incidence and survival rates, as both reported to be higher in developed and high-income countries and regions (8, 23, 45, 46). Better primary care, higher care quality, better access to and higher prevalence of screening programs, and other independent factors lead to higher detection, incidence, and higher prevalence of BC due to better protection against mortality due to cancer in wealthier countries with better sociodemographic indicators (2, 47–49). Therefore, development and implementation of cost-effective screening and treatment interventions are necessary to effectively manage BC burden in lower SDI countries (50).

Results of this study revealed higher MIR and YLL/YLD ratio values for both female and male patients with BC in deprived countries of the region and those extremely affected by war and conflict in the past years including Afghanistan, Yemen, Sudan, Syrian Arab Republic, Iraq, and Libya. Interpreting raw epidemiologic measures is prone to underestimation due to lack of data from some countries with limited registries like those with poorly developed data gathering and reporting systems; thus, we used the two aforementioned indices to explore the BC status among the countries included in this study. The higher values of these two indices indicate poorer prognosis of BC in the countries mostly affected by war. Based on these findings, we hypothesize that in such countries, lack of proper screening programs and utilities, diagnosis of cancer in later and advanced stages with reduced curability, and altered cancer care lead to worse disease outcomes, and this is mainly highlighted in the NAME region (51–53). Regarding the overall decreasing trend of the two mentioned indices in the region and countries, it could be inferred that quality of care of BC had an improved status in the past decades in both female and male patients, which could be attributed to improvements in disease management and development of novel therapeutics, better access to treatment, and earlier detection of BC with improved screening and diagnostics (54–56).

Among the investigated BC risk factors in this study, high FPG and secondhand smoking were found to have the greatest burden. An established association between BC incidence and diabetes mellitus has been discovered before, as diabetes shares biologic pathways that can trigger BC, especially in older patients in postmenopausal stages and obese individuals (57–59). In this regard, more robust screening and preventive measures, and even pharmacological ones like metformin prescription interventions in a diabetic population, have shown promising results and are necessary to effectively prevent BC (60, 61). Also, the proven association between active smoking and BC, specifically smoking before first birth, is shown in literature (62–64). Interestingly, the current study found secondhand smoke to be a major risk factor of BC in both female and male patients. Similarly, recent studies also found the relation between passive smoking and risk of BC, and this highlights the importance of protecting women at a higher risk of BC due to exposure to secondhand smoke (42, 65).

A thorough investigation in the countries of the NAME region reveals practical points regarding the epidemiology of BC and the attributable risks of this condition. Many countries located in this region are developing, low- and middle-income countries (LMICs) coping with major obstacles to public health improvement, such as war and conflict and shortages in resources and infrastructures, leading to sub-optimal BC detection and care, including management and treatment (17, 66, 67). The poor prognosis of BC in these countries is mainly due to the detection of disease in advanced stages ending in poor outcomes and lower survival rates (68, 69). The 10-year survival rates of BC in this region vary between countries of the region and in comparison to developed countries (70). The frequent presence of the BRCA1 gene mutations in some countries of this region, which is a major risk factor of BC incidence, should be noted and various screening programs should be implemented to handle the burden of BC in NAME (71, 72). Multiple barriers to early access to screening and care for BC in different health system, health provider, and patient levels in the NAME region need to be addressed to improve BC status in this region (18). Psychological impact and socio-cultural issues are also major problems that influence the care of patients with BC in this region (73).

The importance of BC globally and regionally yielded many initiatives and preventive and surveillance programs to improve BC status. One of the most known ones is the National Comprehensive Cancer Network (NCCN), a union of leading cancer centers in the United States that provides cancer standard of care guidelines—named the NCCN Clinical Practice Guidelines in Oncology—for many cancers, including BC (74, 75). This guideline generally provides the most recent evidence-based information on the basics of BC, testing for BC, and treatment options of the condition (74). In order to implement the mentioned guidelines in the NAME region, the NCCN-MENA initiative first adopted the 2009 version of the guideline and modified it based on regional characteristics, especially risk factor-related items, since the profile varies among regions (10, 76). Development and update of such practical guidelines could successfully lead to a better care of BC in this region. Another example is the Breast Health Global Initiative (BHGI) that tries to control incidence and burden of BC in LMICs through behavior modification, reducing BC mortality by earlier detection of the condition, promotion of BC awareness, education, and self-examination, and modifying BC screening and treatment programs based on countries’ resources (17). A comprehensive framework developed for national BC control strategies mainly in countries located in Asia, Latin America, and the NAME region, proposed that four major actions are needed to successfully implement such framework, including building necessary infrastructures, expanding evidence, eliminating obstacles, and promoting patient support (21). Another promising progress in BC control in NAME was the implementation of a regional framework on cancer prevention and control in 2017, proposing public health approaches to regional policymakers to effectively control the most prevalent cancers of the region including BC, cervical cancer, and colorectal cancer (77). Also, the WHO’s regional guide on early detection of the most common cancers provides clear guidance on BC early detection approaches in line with local resources (78).

A more in-depth investigation of ongoing BC screening programs in the countries of this region using the WHO GHO database showed active programs in all countries in 2019 except for Afghanistan, Libya, Sudan, and Yemen. Also, this database revealed that except for Jordan, Libya, Syrian Arab Republic, and Yemen, all countries of this region had an operational policy/strategy/action plan for cancer in 2019. Additionally, a population-based cancer registry was available in all countries of the region except for Libya, Syrian Arab Republic, and Yemen in 2019 (35). Additional data on these indicators in different years are provided in **Supplementary Tables 8–10**. Measuring the estimated overall UHC index and the specific index on BC treatment showed the highest values for Kuwait, Qatar, and Lebanon and the lowest in Afghanistan, Yemen, and Sudan in 2019 (**Supplementary Table 11**) (36). The supplied material could help health authorities and policymakers in providing further actions to control the BC epidemic in the region.

The major limitations of this study include the nature of the main endeavor by IHME to estimate epidemiology and burden of BC and its risk factors. Availability and quality of the primary data and data processing and modeling complications are the main challenges in data estimation in GBD studies (33, 34). Despite these limitations, IHME tries to reassess and reevaluate its data processing method for each round of study, and the refinements lead to more stability of provided data. For the GBD 2019 study, for example, the clarification of the reference and alternative methods for measuring outcomes and the bias mapping from alternative to reference ones and enhancements in modeling by implementation of standard locations for estimating effects in models were conducted to produce the most precise estimations (33, 34). Regarding the BC epidemiologic measures, lack of data on various histopathologic subtypes of this cancer in the GBD study is the major shortcoming of this study. In GBD 2019 risk studies, reassessments of dose–response relationships and further investigations on the joint effects of risk factors were taken to make the risk estimations more robust (34). Regarding the included risk factors in this study, the major concern is the numerous risks of BC missed in the GBD study including genetic risk factors, BRCA, family history, radiation, hormone therapy, breastfeeding, early menstruation or late menopausal age, and number of children. Furthermore, another limitation of this study is the absence of estimation on some of the risk factors due to data scarcity, which complicates BC burden estimation in male population. Considering all these limitations, IHME tries to provide the most accurate epidemiologic data on diseases and risk factors through its meticulous methodologies. This study is a part of this project and explored BC epidemiology in the NAME region and provided data in the regional and national levels. Results of this study could successfully guide health authorities in multiple levels to adopt proper policies and needed actions to control the growing burden of BC in this region.

## Conclusions

BC receives a special consideration when investigating the status of cancer in the NAME region. The increasing incidence and burden rates of BC in this region is remarkable, especially when considering limited resources and policies in the developing countries of this region, which make up a great proportion. Also, considering the presence of war and conflict in a number of countries in this region, the BC epidemic is prone to underestimation and mismanagement. Further investigations and careful resource allocations besides proper population-wide policies are needed to effectively mitigate the growing burden of this cancer in countries located in this region.

## Data availability statement

Publicly available datasets were analyzed in this study. This data can be found here: the Global Burden of Disease study data repository [https://vizhub.healthdata.org/gbd-results/].

## Ethics statement

The studies involving human participants were reviewed and approved by the institutional review board of the Endocrinology and Metabolism Research Institute at Tehran University of Medical Sciences (IR.TUMS.EMRI.REC.1400.031). Written informed consent for participation was not required for this study in accordance with the national legislation and the institutional requirements.

## Author contributions

All authors listed have made a substantial, direct, and intellectual contribution to the work and approved it for publication. Please see the **Supplementary Material** (pages 44-45) for more detailed information about individual author contributions to the research, divided into the following categories: providing data or critical feedback on data sources; developing methods or computational machinery; providing critical feedback on methods or results; drafting the work or revising is critically for important intellectual content; and managing the estimation or publications process.

## Group members of GBD 2019 NAME Breast Cancer Collaborators

Sina Azadnajafabad, MD,^1^ Sahar Saeedi Moghaddam, MSc,^1^ Esmaeil Mohammadi, MD,^1,2^ Negar Rezaei, PhD,^1,3^ Mohammad-Mahdi Rashidi, MD^1^, Nazila Rezaei, MD,^1^ Mohsen Abbasi-Kangevari, MD,^1^ Zeinab Abbasi-Kangevari, BSc,^4,1^ Hedayat Abbastabar, PhD,^5^ Hassan Abidi, PhD,^6^ Hassan Abolhassani, PhD,^7,8^ Prof Hiwa Abubaker Ali, PhD,^9^ Eman Abu-Gharbieh, PhD,^10^ Prof Juan Manuel Acuna, MD,^11,12^ Sima Afrashteh, PhD,^13,14^ Muhammad Sohail Afzal, PhD,^15^ Sajjad Ahmad, PhD,^16^ Ali Ahmadi, PhD,^17,18^ Sepideh Ahmadi, PhD,^19^ Haroon Ahmed, PhD,^20^ Luai A. Ahmed, PhD,^21^ Tarik Ahmed Rashid, PhD,^22^ Marjan Ajami, PhD,^23,24^ Mostafa Akbarzadeh-Khiavi, PhD,^25^ Hanadi Al Hamad, MD,^26,27^ Mohammed Khaled Al-Hanawi, PhD,^28,29^ Beriwan Abdulqadir Ali, PhD,^30,31^ Yousef Alimohamadi, PhD,^32^ Sadaf Alipour, MD,^33^ Prof Syed Mohamed Aljunid, PhD,^34,35^ Sami Almustanyir, MD,^36,37^ Rajaa M Al-Raddadi, PhD,^38^ Saba Alvand, MD,^39^ Jalal Arabloo, PhD,^40^ Morteza Arab-Zozani, PhD,^41^ Armin Aryannejad, MD,^1,42^ Tahira Ashraf, MS,^43^ Seyyed Shamsadin Athari, PhD,^44^ Amirhossein Azari Jafari, MD,^45^ Nader Bagheri, PhD,^46^ Sara Bagherieh, BSc,^47^ Saeed Bahadory, PhD,^48,49^ Mohammad Behnammoghadam, MSc,^50,51^ Akshaya Srikanth Bhagavathula, PhD,^52^ Somayeh Bohlouli, PhD,^53^ Prof Wolfram Manuel Wolfram Brück, PhD,^54^ Aso Mohammad Darwesh, PhD,^55^ Mostafa Dianatinasab, MSc,^56,14^ Fariba Dorostkar, PhD,^57^ Ebrahim Eini, DDS,^58^ Iffat Elbarazi, DrPH,^21^ Muhammed Elhadi, MD,^59^ Iman El Sayed, PhD,^60^ Prof Maysaa El Sayed Zaki, PhD,^61^ Sharareh Eskandarieh, PhD,^62^ Ibtihal Fadhil, PhD,^63^ Fatemeh Farahmand, BSc,^64^ Pawan Sirwan Faris, PhD,^65,66^ Nasrin Galehdar, PhD,^67^ Mansour Ghafourifard, PhD,^68^ Seyyed-Hadi Ghamari, MD,^1,4^ Azin Ghamari, MD,^1^ Ahmad Ghashghaee, BSc,^69^ Maryam Gholamalizadeh, PhD,^70^ Kimiya Gohari, MS,^71,1^ Pouya Goleij, MSc,^72^ Mohamad Golitaleb, PhD,^73^ Mohammed Ibrahim Mohialdeen Gubari, PhD,^74^ Nima Hafezi-Nejad, MD,^75,3^ Prof Rabih Halwani, PhD,^10,76^ Prof Randah R Hamadeh, PhD,^77^ Prof Samer Hamidi, DrPH,^78^ Asif Hanif, PhD,^79^ Shafiul Haque, PhD,^80^ Soheil Hassanipour, PhD,^81,82^ Khezar Hayat, MS,^83,84^ Mohammad Heidari, PhD,^85^ Zahra Heidarymeybodi, MD,^86^ Mohammad-Salar Hosseini, MD,^87^ Mehdi Hosseinzadeh, PhD,^88,89^ Prof Mohamed Hsairi, MPH,^90^ Elham Jamshidi, PharmD,^91,92^ Amirreza Javadi Mamaghani, PhD,^93,94^ Tahereh Javaheri, PhD,^95^ Ali Kabir, MD,^96^ Leila R Kalankesh, PhD,^97^ Leila Keikavoosi-Arani, PhD,^98^ Prof Yousef Saleh Khader, PhD,^99^ Ejaz Ahmad Khan, MPH,^100^ Prof Gulfaraz Khan, PhD,^101^ Maryam Khayamzadeh, MD,^102,103^ Behzad Kiani, PhD,^104^ Hamid Reza Koohestani, PhD,^105^ Prof Sang-woong Lee, PhD,^106^ Mozhgan Letafat-nezhad, PhD,^107^ Somayeh Livani, MD,^108^ Farzan Madadizadeh, PhD,^109^ Ahmad R Mafi, MD,^110^ Prof Soleiman Mahjoub, PhD,^111,112^ Prof Ata Mahmoodpoor, MD,^113^ Marzieh Mahmoudimanesh, PhD,^114^ Mohammad-Reza Malekpour, MD,^1^ Ahmad Azam Malik, PhD,^115,79^ Prof Fariborz Mansour-Ghanaei, MD,^81,82^ Mohammad Ali Mansournia, PhD,^116^ Seyedeh Zahra Masoumi, PhD,^117^ Entezar Mehrabi Nasab, MD,^118^ Seyyedmohammadsadeq Mirmoeeni, MD,^45^ Yousef Mohammad, MD,^119^ Karzan Abdulmuhsin Mohammad, PhD,^65^ Mokhtar Mohammadi, PhD,^120^ Arif Mohammed, PhD,^121^ Sara Momtazmanesh, MD,^1,3^ Prof Abdolvahab Moradi, PhD,^122^ Paula Moraga, PhD,^123^ Abbas Mosapour, PhD,^112,124^ Atta Abbas Naqvi, PhD,^125^ Zuhair S Natto, DrPH,^126,127^ Seyed Aria Nejadghaderi, MD,^1,128^ Prof Marzieh Nojomi, MD,^129,130^ Maryam Noori, MD,^131^ Hassan Okati-Aliabad, PhD,^132^ Reza Pakzad, PhD,^133^ Hamidreza Pazoki Toroudi, PhD,^134,135^ Zahra Zahid Piracha, PhD,^136^ Naeimeh Pourtaheri, PhD,^137^ Navid Rabiee, PhD,^138,139^ Prof Alireza Rafiei, PhD,^140,141^ Fakher Rahim, PhD,^142^ Shahram Rahimi-Dehgolan, MD,^143^ Amir Masoud Rahmani, PhD,^144^ Vahid Rahmanian, PhD,^145^ Mahsa Rashidi, DVM,^146^ Amirfarzan Rashidi, MD,^147^ Reza Rawassizadeh, PhD,^148^ Mohammad Sadegh Razeghinia, MSc,^149,150^ Prof Nima Rezaei, PhD,^7,151^ Siamak Sabour, PhD,^18^ Basema Saddik, PhD,^152^ Prof Umar Saeed, PhD,^136,153^ Maryam Sahebazzamani, MSc,^154,155^ Amirhossein Sahebkar, PhD,^156,157^ Abdallah M Samy, PhD,^158^ Muhammad Arif Nadeem Saqib, PhD,^159,160^ Brijesh Sathian, PhD,^26,161^ Sayed Mohammad Shafiee, PhD,^162^ Saeed Shahabi, PhD,^163^ Fariba Shahraki-Sanavi, PhD,^132^ Masood Ali Shaikh, MD,^164^ Ali Sheidaei, MSc,^1,116^ Rahim Ali Sheikhi, BHlthSci,^165^ Reza Shirkoohi, PhD,^166,167^ Parnian Shobeiri, MD,^1,3^ Mohammad Sadegh Soltani-Zangbar, MSc,^168^ Elnaz Tabibian, MD,^169^ Majid Taheri, PhD,^170,171^ Moslem Taheri Soodejani, PhD,^172^ Mohamad-Hani Temsah, MD,^173^ Amir Tiyuri, MSc,^174,175^ Irfan Ullah, PhD,^15^ Sana Ullah, PhD,^176,177^ Sahel Valadan Tahbaz, PhD,^178,179^ Rohollah Valizadeh, PhD,^180^ Bay Vo, PhD,^181^ Prof Yasir Waheed, PhD,^182^ Seyed Hossein Yahyazadeh Jabbari, MD,^178^ Fereshteh Yazdanpanah, MD,^183,184^ Deniz Yuce, MD,^185^ Prof Nazar Zaki, PhD,^186^ Iman Zare, BSc,^187^ Mohammad Zoladl, PhD,^188^ Ali H Mokdad, PhD,^189,190^ Prof Mohsen Naghavi, PhD,^189,190^ Prof Christopher J L Murray, DPhil,^189,190^ Prof Bagher Larijani, MD,^3^ Prof Farshad Farzadfar, DSc^1,3^

## Affiliations

^1^Non-Communicable Diseases Research Center, Endocrinology and Metabolism Population Sciences Institute, Tehran University of Medical Sciences, Tehran, Iran

^2^School of Medicine, Tehran University of Medical Sciences, Tehran, Iran

^3^Endocrinology and Metabolism Research Center, Endocrinology and Metabolism Clinical Sciences Institute, Tehran University of Medical Sciences, Tehran, Iran

^4^Social Determinants of Health Research Center, Shahid Beheshti University of Medical Sciences, Tehran, Iran

^5^Advanced Diagnostic and Interventional Radiology Research Center, Tehran University of Medical Sciences, Tehran, Iran

^6^Laboratory Technology Sciences Department, Yasuj University of Medical Sciences, Yasuj, Iran

^7^Research Center for Immunodeficiencies, Tehran University of Medical Sciences, Tehran, Iran

^8^Department of Biosciences and Nutrition, Karolinska University Hospital, Huddinge, Sweden

^9^Department of Banking and Finance, University of Human Development, Sulaymaniyah, Iraq

^10^Clinical Sciences Department, University of Sharjah, Sharjah, United Arab Emirates

^11^Department of Epidemiology and Population Health, Khalifa University, Abu Dhabi, United Arab Emirates

^12^FIU Robert Stempel College of Public Health & Social Work, Florida International University, Miami, FL, USA

^13^Department of Public Health, Bushehr University of Medical Sciences, Bushehr, Iran

^14^Department of Epidemiology, Shiraz University of Medical Sciences, Shiraz, Iran

^15^Department of Life Sciences, University of Management and Technology, Lahore, Pakistan

^16^Department of Health and Biological Sciences, Abasyn University, Peshawar, Pakistan

^17^Department of Epidemiology and Biostatistics, Shahrekord University of Medical Sciences, Shahrekord, Iran

^18^Department of Epidemiology, Shahid Beheshti University of Medical Sciences, Tehran, Iran

^19^School of Advanced Technologies in Medicine, Shahid Beheshti University of Medical Sciences, Tehran, Iran

^20^Department of Biosciences, COMSATS Institute of Information Technology, Islamabad, Pakistan

^21^Institute of Public Health, United Arab Emirates University, Al Ain, United Arab Emirates

^22^Department of Computer Science and Engineering, University of Kurdistan Hewler, Erbil, Iraq

^23^Department of Food and Nutrition Policy and Planning Research, National Institute of Nutrition, Tehran, Iran

^24^National Nutrition and Food Technology Research Institute, Shahid Beheshti University of Medical Sciences, Iran

^25^Liver and Gastrointestinal Diseases Research Center, Tabriz University of Medical Sciences, Tabriz, Iran

^26^Geriatric and Long Term Care Department, Hamad Medical Corporation, Doha, Qatar

^27^Rumailah Hospital, Hamad Medical Corporation, Doha, Qatar

^28^Department of Health Services and Hospital Administration, King Abdulaziz University, Jeddah, Saudi Arabia

^29^Health Economics Research Group, King Abdulaziz University, Jeddah, Saudi Arabia

^30^Erbil Technical Health College, Erbil Polytechnic University, Erbil, Iraq

^31^School of Pharmacy, Tishk International University, Erbil, Iraq

^32^Health Research Center, Baqiyatallah University of Medical Sciences, Tehran, Iran

^33^Breast Disease Research Center, Cancer Institute, Tehran University of Medical Sciences, Tehran, Iran

^34^Department of Health Policy and Management, Kuwait University, Kuwait, Kuwait

^35^International Centre for Casemix and Clinical Coding, National University of Malaysia, Bandar Tun Razak, Malaysia

^36^College of Medicine, Alfaisal University, Riyadh, Saudi Arabia

^37^Ministry of Health, Riyadh, Saudi Arabia

^38^Department of Community Medicine, King Abdulaziz University, Jeddah, Saudi Arabia

^39^Liver and Pancreatobiliary Diseases Research Center, Tehran University of Medical Sciences, Tehran, Iran

^40^Health Management and Economics Research Center, Iran University of Medical Sciences, Tehran, Iran

^41^Social Determinants of Health Research Center, Birjand University of Medical Sciences, Birjand, Iran

^42^Experimental Medicine Research Center, Tehran University of Medical Sciences, Tehran, Iran

^43^University Institute of Radiological Sciences and Medical Imaging Technology, The University of Lahore, Lahore, Pakistan

^44^Department of Immunology, Zanjan University of Medical Sciences, Zanjan, Iran

^45^School of Medicine, Shahroud University of Medical Sciences, Shahroud, Iran

^46^Basic Health Sciences Institute, Shahrekord University of Medical Sciences, Shahrekord, Iran

^47^School of Medicine, Isfahan University of Medical Sciences, Isfahan, Iran

^48^Department of Parasitology, Tarbiat Modares University, Tehran, Iran

^49^Department of Parasitology, Alborz University of Medical Sciences, Karaj, Iran

^50^Department of Critical Care Nursing, Yasuj University, Yasuj, Iran

^51^Department of Critical Care Nursing, Isfahan University of Medical Sciences, Isfahan, Iran

^52^Department of Health, Human Performance and Recreation, University of Arkansas, Fayetteville, AR, USA

^53^Department of Veterinary Medicine, Islamic Azad University, Kermanshah, Iran

^54^Institute of Life Technologies, HES-SO University of Applied Sciences and Arts Western Switzerland, Sion, Switzerland

^55^Department of Information Technology, University of Human Development, Sulaymaniyah, Iraq

^56^Department of Epidemiology, Maastricht University, Maastricht, Netherlands

^57^Department of Medical Laboratory Sciences, Iran University of Medical Sciences, Tehran, Iran

^58^Department of Orthodontics, Ahvaz Jundishapur University of Medical Sciences, Ahvaz, Iran

^59^Faculty of Medicine, University of Tripoli, Tripoli, Libya

^60^Biomedical Informatics and Medical Statistics Department, Alexandria University, Alexandria, Egypt

^61^Clinical Pathology Department, Mansoura University, Mansoura, Egypt

^62^Multiple Sclerosis Research Center, Tehran University of Medical Sciences, Tehran, Iran

^63^Division of Non-Communicable Diseases, Ministry of Public Health and Population, Dubai, United Arab Emirates

^64^Department of Literature and Humanities, Islamic Azad University, Kermanshah, Iran

^65^Department of Biology, Salahaddin University-Erbil, Erbil, Iraq

^66^Department of Biology, Cihan University-Erbil, Erbil, Iraq

^67^Department of Surgical Technology, Lorestan University of Medical Sciences, Khorramabad, Iran

^68^Department of Medical Surgical Nursing, Tabriz University of Medical Sciences, Tabriz, Iran

^69^School of Public Health, Qazvin University of Medical Sciences, Qazvin, Iran

^70^Cancer Research Center, Shahid Beheshti University of Medical Sciences, Tehran, Iran

^71^Department of Biostatistics, Tarbiat Modares University, Tehran, Iran

^72^Department of Genetics, Sana Institute of Higher Education, Sari, Iran

^73^Department of Nursing, Arak University of Medical Sciences, Arak, Iran

^74^Department of Family and Community Medicine, University of Sulaimani, Sulaimani, Iraq

^75^Department of Radiology and Radiological Science, Johns Hopkins University, Baltimore, MD, USA

^76^College of Medicine, University of Sharjah, Sharjah, United Arab Emirates

^77^Department of Family and Community Medicine, Arabian Gulf University, Manama, Bahrain

^78^School of Health and Environmental Studies, Hamdan Bin Mohammed Smart University, Dubai, United Arab Emirates

^79^University Institute of Public Health, The University of Lahore, Lahore, Pakistan

^80^Research & Scientific Studies Unit, Jazan University, Jazan, Saudi Arabia

^81^Gastrointestinal and Liver Diseases Research Center, Guilan University of Medical Sciences, Rasht, Iran

^82^Caspian Digestive Disease Research Center, Guilan University of Medical Sciences, Rasht, Iran

^83^Institute of Pharmaceutical Sciences, University of Veterinary and Animal Sciences, Lahore, Pakistan

^84^Department of Pharmacy Administration and Clinical Pharmacy, Xian Jiaotong University, Xian, China

^85^Community-Oriented Nursing Midwifery Research Center, Shahrekord University of Medical Sciences, Shahrekord, Iran

^86^Research Center, Shahid Sadoughi University of Medical Sciences, Yazd, Iran

^87^Student Research Committee, Tabriz University of Medical Sciences, Tabriz, Iran

^88^Institute of Research and Development, Duy Tan University, Da Nang, Viet Nam

^89^Department of Computer Science, University of Human Development, Sulaymaniyah, Iraq

^90^Faculty of Medicine of Tunis, University Tunis El Manar, Tunis, Tunisia

^91^Functional Neurosurgery Research Center, Shahid Beheshti University of Medical Sciences, Tehran, Iran

^92^Division of Pulmonary Medicine, Lausanne University Hospital (CHUV), Lausanne, Switzerland

^93^Department of Parasitology, Shahid Beheshti University of Medical Sciences, Tehran, Iran

^94^Department of Parasitology, Tabriz University of Medical Sciences, Tabriz, Iran

^95^Health Informatic Lab, Boston University, Boston, MA, USA

^96^Minimally Invasive Surgery Research Center, Iran University of Medical Sciences, Tehran, Iran

^97^School of Management and Medical Informatics, Tabriz University of Medical Sciences, Tabriz, Iran

^98^Department of Healthcare Services Management, Alborz University of Medical Sciences, Karaj, Iran

^99^Department of Public Health, Jordan University of Science and Technology, Irbid, Jordan

^100^Department of Epidemiology and Biostatistics, Health Services Academy, Islamabad, Pakistan

^101^Department of Medical Microbiology & Immunology, United Arab Emirates University, Al Ain, United Arab Emirates

^102^Shahid Beheshti University of Medical Sciences, Tehran, Iran

^103^The Iranian Academy of Medical Sciences, Tehran, Iran

^104^Department of Medical Informatics, Mashhad University of Medical Sciences, Mashhad, Iran

^105^Social Determinants of Health Research Center, Saveh University of Medical Sciences, Saveh, Iran

^106^Pattern Recognition and Machine Learning Lab, Gachon University, Seongnam, South Korea

^107^South Tehran Health network, Tehran University of Medical Sciences, Tehran, Iran

^108^Radiology Department, Golestan University of Medical Sciences, Gorgan, Iran

^109^Department of Biostatistics and Epidemiology, Yazd University of Medical Sciences, Yazd, Iran

^110^Department of Clinical Oncology, Shahid Beheshti University of Medical Sciences, Tehran, Iran

^111^Cellular and Molecular Biology Research Center, Babol University of Medical Sciences, Babol, Iran

^112^Department of Clinical Biochemistry, Babol University of Medical Sciences, Babol, Iran

^113^Anesthesiology and Critical Care, Tabriz University of Medical Sciences, Tabriz, Iran

^114^Department of Biostatistics and Epidemiology, Kerman University of Medical Sciences, Kerman, Iran

^115^Rabigh Faculty of Medicine, King Abdulaziz University, Jeddah, Saudi Arabia

^116^Department of Epidemiology and Biostatistics, School of Public Health, Tehran University of Medical Sciences, Tehran, Iran

^117^Department of Midwifery, Hamadan University of Medical Sciences, Hamadan, Iran

^118^Tehran Heart Center, Tehran University of Medical Sciences, Tehran, Iran

^119^Internal Medicine Department, King Saud University, Riyadh, Saudi Arabia

^120^Department of Information Technology, Lebanese French University, Erbil, Iraq

^121^Department of Biology, University of Jeddah, Jeddah, Saudi Arabia

^122^School of Medicine, Golestan University of Medical Sciences, Gorgan, Iran

^123^Computer, Electrical, and Mathematical Sciences and Engineering Division, King Abdullah University of Science and Technology, Thuwal, Saudi Arabia

^124^Department of Clinical Biochemistry, Tarbiat Modares University, Tehran, Iran

^125^School of Pharmacy, University of Reading, Reading, UK

^126^Department of Dental Public Health, King Abdulaziz University, Jeddah, Saudi Arabia

^127^Department of Health Policy and Oral Epidemiology, Harvard University, Boston, MA, USA

^128^School of Medicine, Shahid Beheshti University of Medical Sciences, Tehran, Iran

^129^Preventive Medicine and Public Health Research Center, Iran University of Medical Sciences, Tehran, Iran

^130^Department of Community and Family Medicine, Iran University of Medical Sciences, Tehran, Iran

^131^Student Research Committee, Iran University of Medical Sciences, Tehran, Iran

^132^Health Promotion Research Center, Zahedan University of Medical Sciences, Zahedan, Iran

^133^Department of Epidemiology, Ilam University of Medical Sciences, Ilam, Iran

^134^Department of Physiology, Iran University of Medical Sciences, Tehran, Iran

^135^Physiology Research Center, Iran University of Medical Sciences, Tehran, Iran

^136^International Center of Medical Sciences Research, Islamabad, Pakistan

^137^Non-communicable Diseases Research Center, Bam University of Medical Sciences, Bam, Iran

^138^School of Engineering, Macquarie University, Sydney, NSW, Australia

^139^Pohang University of Science and Technology, Pohang, South Korea

^140^Department of Immunology, Mazandaran University of Medical Sciences, Sari, Iran

^141^Molecular and Cell Biology Research Center, Mazandaran University of Medical Sciences, Sari, Iran

^142^Department of Anesthesia, Cihan University of Sulaimaniya, Sulaimaniya, Iraq

^143^Physical Medicine and Rehabilitation, Tehran University of Medical Sciences, Tehran, Iran

^144^Future Technology Research Center, National Yunlin University of Science and Technology, Yunlin, Taiwan

^145^Department of Community Medicine, Jahrom University of Medical Sciences, Jahrom, Iran

^146^Department of Clinical Science, Islamic Azad University, Garmsar, Iran

^147^Rafsanjan University of Medical Sciences, Rafsanjan, Iran

^148^Department of Computer Science, Boston University, Boston, MA, USA

^149^Department of Immunology and Laboratory Sciences, Sirjan School of Medical Sciences, Sirjan, Iran

^150^Department of Immunology, Kerman University of Medical Sciences, Kerman, Iran

^151^Network of Immunity in Infection, Malignancy and Autoimmunity (NIIMA), Universal Scientific Education and Research Network (USERN), Tehran, Iran

^152^Sharjah Institute for Medical Research, University of Sharjah, Sharjah, United Arab Emirates

^153^Department of Pathology and Microbiology, Jinnah Medical College, Peshawar, Pakistan

^154^Department of Medical Biochemistry, Rafsanjan University of Medical Sciences, Rafsanjan, Iran

^155^Medical Laboratory Sciences, Sirjan School of Medical Sciences, Sirjan, Iran

^156^Applied Biomedical Research Center, Mashhad University of Medical Sciences, Mashhad, Iran

^157^Biotechnology Research Center, Mashhad University of Medical Sciences, Mashhad, Iran

^158^Department of Entomology, Ain Shams University, Cairo, Egypt

^159^Research Development Coordination Section, Pakistan Health Research Council, Islamabad, Pakistan

^160^School of Sciences, University of Management and Technology, Lahore, Pakistan

^161^Faculty of Health & Social Sciences, Bournemouth University, Bournemouth, UK

^162^Department of Clinical Biochemistry and Autophagy Research Center, Shiraz University of Medical Sciences, Shiraz, Iran

^163^Health Policy Research Center, Shiraz University of Medical Sciences, Shiraz, Iran

^164^Independent Consultant, Karachi, Pakistan

^165^Department of Health in Disasters and Emergencies, Shahrekord University of Medical Sciences, Shahrekord, Iran

^166^Cancer Research Center, Tehran University of Medical Sciences, Tehran, Iran

^167^Cancer Biology Research Center, Tehran University of Medical Sciences, Tehran, Iran

^168^Department of Immunology, Tabriz University of Medical Sciences, Tabriz, Iran

^169^Radiology Department, Tehran University of Medical Sciences, Tehran, Iran

^170^Trauma and Injury Research Center, Iran University of Medical Sciences, Tehran, Iran

^171^Medical Ethics and Law Research Center, Shahid Beheshti University of Medical Sciences, Tehran, Iran

^172^Department of Biostatistics and Epidemiology, Shahid Sadoughi University of Medical Sciences, Yazd, Iran

^173^Pediatric Intensive Care Unit, King Saud University, Riyadh, Saudi Arabia

^174^Department of Epidemiology and Biostatistics, Birjand University of Medical Sciences, Birjand, Iran

^175^Department of Epidemiology and Biostatistics, Iran University of Medical Sciences, Tehran, Iran

^176^Department of Zoology, University of Education, Lahore, Lahore, Pakistan

^177^Division of Science and Technology, University of Education, Lahore, Lahore, Pakistan

^178^Clinical Cancer Research Center, Milad General Hospital, Tehran, Iran

^179^Department of Microbiology, Islamic Azad University, Tehran, Iran

^180^Urmia University of Medical Science, Urmia, Iran

^181^Faculty of Information Technology, HUTECH University, Ho Chi Minh City, Viet Nam

^182^Shaheed Zulfiqar Ali Bhutto Medical University (SZABMU), Islamabad, Pakistan

^183^Department of Pediatric Allergy and Immunology, Tabriz University of Medical Sciences, Tabriz, Iran

^184^Department of Pediatric Allergy and Immunology, Tehran University of Medical Sciences, Tehran, Iran

^185^Cancer Institute, Hacettepe University, Ankara, Türkiye

^186^Computer Science and Software Engineering/Big Data Analytics Center, United Arab Emirates University, Al Ain, United Arab Emirates

^187^Research and Development Department, Sina Medical Biochemistry Technologies, Shiraz, Iran

^188^Department of Nursing, Yasuj University of Medical Sciences, Yasuj, Iran

^189^Institute for Health Metrics and Evaluation, University of Washington, Seattle, WA, USA

^190^Department of Health Metrics Sciences, School of Medicine, University of Washington, Seattle, WA, USA
