# Supplementary material for: Burden of breast cancer and attributable risk factors in the North Africa and Middle East region, 1990–2019: a systematic analysis for the Global Burden of Disease Study 2019
Source: Front Oncol. 2023 Aug 1;13:1132816. doi: 10.3389/fonc.2023.1132816 (PMC10431599; doi:10.3389/fonc.2023.1132816)
Supplement: Supplementary file 1 [file DataSheet_1.pdf]

Supplementary appendix for "Burden of breast cancer and attributable risk factors in the North Africa and Middle East region, 1990-2019: a systematic analysis for the Global Burden of Disease Study 2019"

This appendix provides supplemental methods and results.

List of material

Content **Page**

Supplementary methods **5**

A. IHME estimation framework for breast cancer **5**
B. Decomposition analysis of breast cancer incidence **9**

Supplementary figure 1. The Global Burden of Disease (GBD) 2019 cancer incidence, prevalence, and years lived with disability (YLDs) estimation flow chart. **7**

Supplementary figure 2. The Global Burden of Disease (GBD) 2019 cause of death, mortality, and years of life lost (YLLs) estimation flow chart. **8**

Supplementary table 1. Data sources used to generate breast cancer estimations in the North Africa and Middle East countries, for the period 1990-2019. **11**

Supplementary table 2. Relative risks used by age and sex for attributable burden estimation of each risk factor's contribution to breast cancer burden. **19**

Supplementary table 3. The estimated socio-demographic index (SDI) values for the NAME region and included countries in this study for the investigated time points in the analysis of results. **24**

Supplementary table 4. The estimated Healthcare Access and Quality (HAQ) Index for the North Africa and Middle East countries, based on the Global Burden of Disease Study 2019. **25**

Supplementary table 5. Incidence, prevalence, deaths, YLLs (Years of Life Lost), YLDs (Years Lived with Disability), DALYs (Disability-Adjusted Life Years) of breast cancer in 21 countries of the North Africa and Middle East region, in 1990 and 2019. **26**

Supplementary table 6. All-ages numbers of deaths, DALYs, YLLs, and YLDs attributed to risk factors of breast cancer in 1990 and 2019 and the percent change in this period. **30**

Supplementary table 7. Age-standardized rates per 100000 deaths, DALYs, YLLs, and YLDs, attributed to risk factors of breast cancer in 1990 and 2019 and the percent change in this period. **31**

Supplementary table 8. Existence of national screening program for breast cancer, data provided by the Global Health Observatory (GHO), powered by the World Health Organization. **32**

Supplementary table 9. Existence of operational policy/strategy/action plan for cancer, data provided by the Global Health Observatory (GHO), powered by the World Health Organization. **33**

Supplementary table 10. Existence of population-based cancer registry, data provided by the Global Health Observatory (GHO), powered by the World Health Organization. **34**

Supplementary table 11. The Universal Health Coverage (UHC) effective coverage index and the breast cancer treatment component estimations for the countries of the North Africa and Middle East region, provided by Global Burden of Disease Study 2019. **35**

Supplementary figure 3. Age-standardized rates of incidence, prevalence, deaths, and DALYs of breast cancer stratified by sex, in different age groups in the North Africa and Middle East region, in 1990 and 2019. **36**

Supplementary figure 4. The all-ages number of incidences, prevalence, deaths, and DALYs of breast cancer by age groups during the 1990-2019 period in females of the North Africa and Middle East region. **37**

Supplementary figure 5. The all-ages number of incidences, prevalence, deaths, and DALYs of breast cancer by countries during the 1990-2019 period in females of the North Africa and Middle East region. **38**

Supplementary figure 6. Time trend of age-standardized rates of incidence, prevalence, deaths, and DALYs of breast cancer for both sexes by countries, 1990-2019. **39**

Supplementary figure 7. Scatter of age-standardized rates of incidence, prevalence, deaths, and DALYs of breast cancer by countries in the North Africa and Middle East region during the 1990-2010 against 2010-2019 period. **40**

Supplementary figure 8. Ranking of age-standardized rates of female breast cancer prevalence and DALYs by countries in the North Africa and Middle East region, in 1990 and 2019. **41**

Supplementary figure 9. Age-standardized rates of deaths, DALYs, YLLs, and YLDs attributed to breast cancer risk factors in females in the North Africa and Middle East region in 1990. **42**

Supplementary figure 10. Age-standardized rates of deaths, DALYs, YLLs, and YLDs attributed to breast cancer risk factors in females in the North Africa and Middle East region in 2019. **43**

Authors' Contributions **44**

Supplementary methods

A. IHME estimation framework for breast cancer: full description

Breast cancer (BC) with B.1.14 code in GBD cause list and the associate International Classification of Diseases and Injuries-10 (ICD-10) codes C50-C50.9, D05-D05.9, D24-D24.9, D48.6, D49.3, and ICD-9 codes 174-175.9, 217-217.8, 233.0, 238.3, 239.3, 610-610.9 mapped to this specific GBD cause as BC mortality data and ICD-10 codes C50-C50.629, C50.8-C50.929, Z12.3-Z12.39, Z80.3, Z85.3, Z86.000 and ICD-9 codes 174-175.9, V10.3, V16.3 mapped to BC incidence data, was the focused cause in this study. (1, 2)

IHME uses various databases and modellings to estimate the most accurate data on the epidemiology of diseases and risk factors. The basis of estimations is mortality estimation made on the cause of death (CoD) database containing information from sources of vital registration, verbal autopsy, cancer registry, surveillance, police records, sibling history, survey/census, and minimally invasive tissue samples diagnoses, which the main data on cancers mortality is derived from the first three sources. Cancer incidence is retrieved from individual or aggregated cancer registry databases like Cancer Incidence in Five Continents (CI5), European Union Registration (EUREG), or NORDCAN, a cancer database for the Nordic countries. The full list of data sources used to estimate BC data for the NAME region in this study is provided in the Supplementary table 1.

Cancer registries including both incidence and mortality data of cancers are used to obtain mortality to incidence ratios (MIR). The three indices of mortality, incidence, and MIR are then aggregated and estimated by several calculations to provide the most precise information on cancers. All included causes in these databases are mapped into GBD causes classifications before entering any modelling, which hierarchy of causes explained in the previous section. (1, 2) In brief, modeling strategy takes the modelled mortality and MIR (with covariates of sex, categorical age, and the healthcare-access and quality index (HAQI)) to model the incidence values. IHME uses the CODEm (Cause of Death Ensemble modeling) modeling strategy for individual cause of death models in GBD 2019. The covariates used in CODEm models for BC were liters of alcohol consumed per capita (direction: +), mean BMI (+), log-transformed summary exposure value (SEV) scalar (+), age-specific fertility rate (-), age- and sex-specific SEV for low fruit and vegetables (+), cumulative cigarettes (10 and 20 years) (+), smoking prevalence (+), diabetes fasting plasma glucose (mmol/L) age-standardized 25+ (+), Healthcare Access and Quality Index (HAQI) (-), lag-distributed income (LDI) (I$ per capita) (-), and Socio-demographic Index (+). (1)

In the next step, prevalence is estimated for 10-year survival after incidence -derived from the Surveillance, Epidemiology, and End Results (SEER) program- and in cases containing procedures like BC having mastectomy in some patients, prevalence is extended for more than 10-years. In order to estimate years lived with disability (YLDs), total prevalence is distributed into four sequelae of diagnosis and primary therapy, controlled

phase, metastatic phase, and terminal phase. Also, the disability associated with mastectomy is added to these four categories and YLDs are calculated based on these five sections (Supplementary figure 1). Years of life lost (YLLs) are estimated from the produced mortality values from the CODEm model following adjusting by location, year, age, and sex for specific cancer type in the CodCorrect model (Supplementary figure 2). Summation of YLDs and YLLs results in disability-adjusted life years (DALYs) as the total burden estimation of BC. SDI is the geometric mean of total fertility rate for individuals younger than 25 years old, mean education for individuals 15 years old and older, and lag-dependent income per capita. SDI estimations were scaled 0 to 100 after calculation in GBD 2019. Countries are grouped in 5 SDI quintiles including low, low-middle, middle, high-middle, and high for an easier comparison of indices in different SDI states. (1)

Risk factor estimations are made by the comparative risk assessment (CRA) conceptual framework that provides a hierarchy of risks and causes contributing to health outcomes. Three main categories of risks mentioned before, are organized in four hierarchical levels, and at each level it is investigated the impact of risk combinations. CRA framework generally provides two types of risk assessment, attributable burden defined as the reduction in the present burden of disease in case of past population exposure to a risk, and avoidable burden defined as the potential reduction in the future burden of a disease in case of change in current risk exposure. The effect size estimation in GBD is done by the relative risk (RR) modeling for level of exposure to risk or cause for mortality or morbidity. (3) Relative risks used by age and sex for attributable burden estimation of each risk factor's contribution to BC burden in this study are provided in the Supplementary table 2.

References

1. GBD 2019 Diseases and Injuries Collaborators. Global burden of 369 diseases and injuries in 204 countries and territories, 1990-2019: a systematic analysis for the Global Burden of Disease Study 2019. Lancet (London, England). 2020;396(10258):1204-22.
2. Global Burden of Disease Cancer Collaboration, Fitzmaurice C, Abate D, Abbasi N, Abbastabar H, Abd-Allah F, et al. Global, Regional, and National Cancer Incidence, Mortality, Years of Life Lost, Years Lived With Disability, and Disability-Adjusted Life-Years for 29 Cancer Groups, 1990 to 2017: A Systematic Analysis for the Global Burden of Disease Study. JAMA oncology. 2019;5(12):1749-68.
3. GBD 2019 Risk Factors Collaborators. Global burden of 87 risk factors in 204 countries and territories, 1990-2019: a systematic analysis for the Global Burden of Disease Study 2019. Lancet (London, England). 2020;396(10258):1223-49.

Supplementary figure 1. The Global Burden of Disease (GBD) 2019 cancer incidence, prevalence, and years lived with disability (YLDs) estimation flow chart.

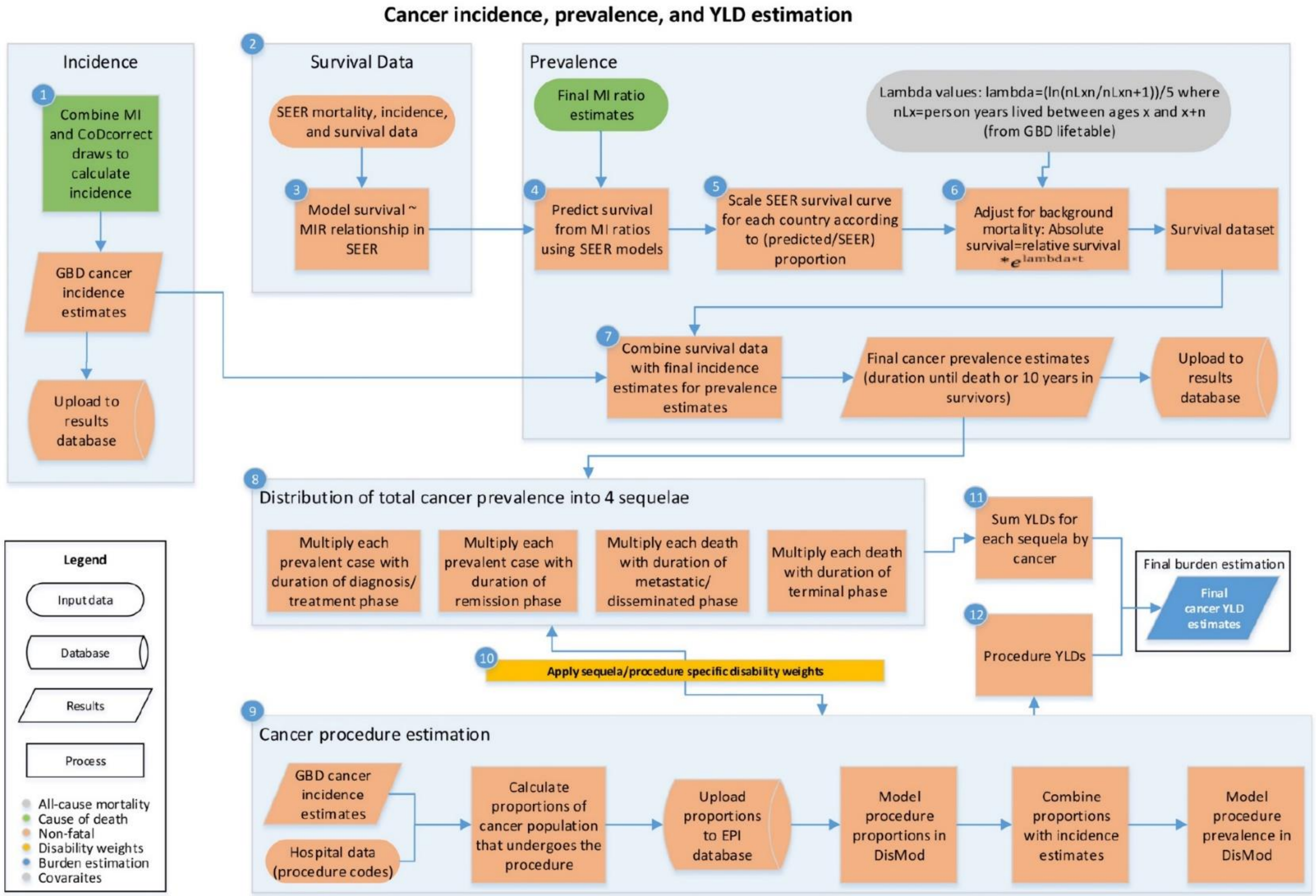

Supplementary figure 2. The Global Burden of Disease (GBD) 2019 cause of death, mortality, and years of life lost (YLLs) estimation flow chart.

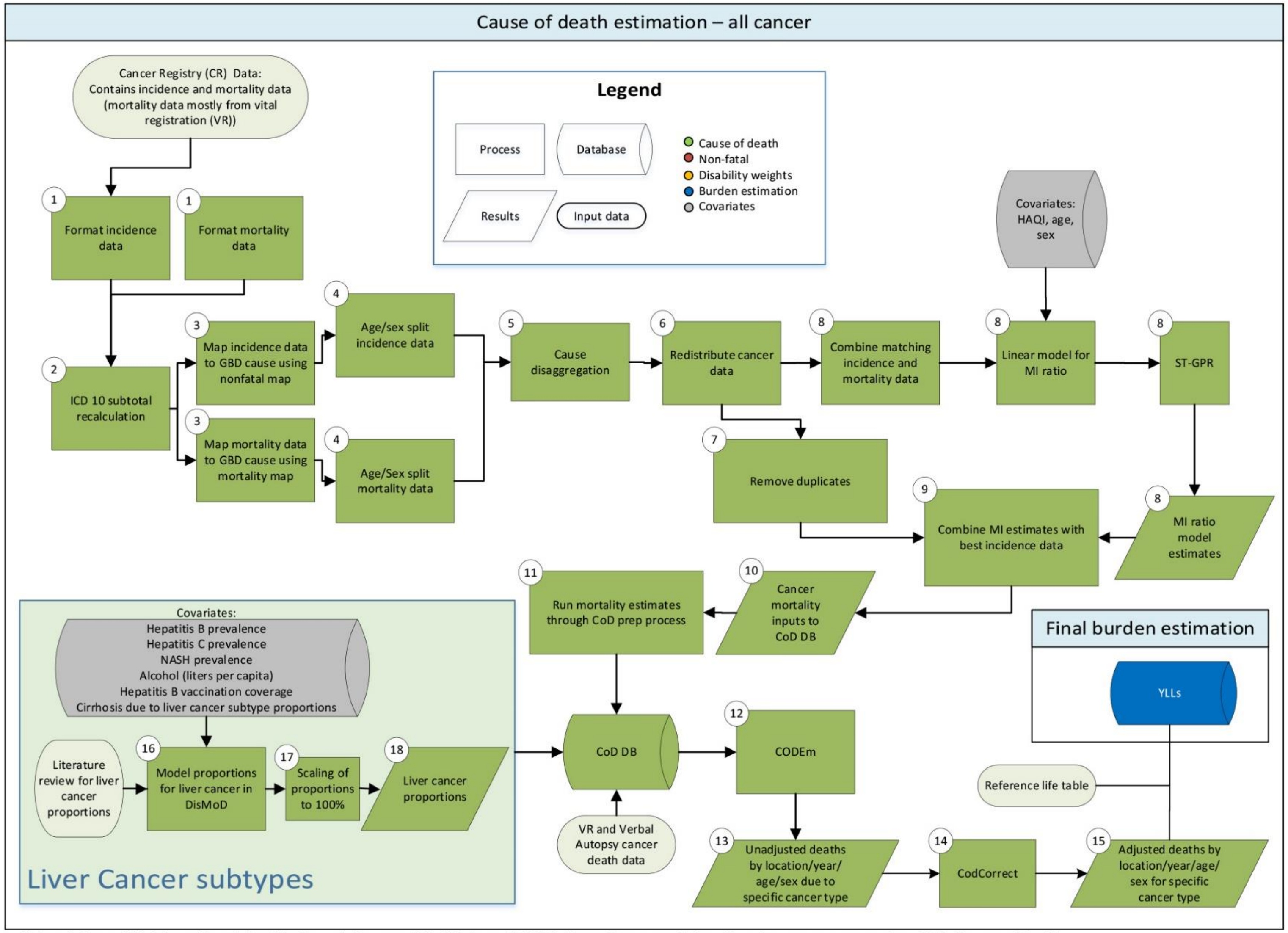

Abbreviations: ICD: International classification of diseases; DB: database, ST-GPR: Space-time smoothing, Gaussian process regression, COD: Causes of death

B. Decomposition analysis of breast cancer incidence

This section describes the steps and formulas of the decomposition analysis of breast cancer incidence.

	Year							
	1990				2019			
Age	New cases	Incidence rate	Sub population	Total population	New cases	Incidence rate	Sub population	Total population
1	a_1	b_1	c_1	D	e_1	f_1	g_1	H
2	a_2	b_2	c_2	D	e_2	f_2	g_2	H
…	…	…	…	D	…	…	…	H
k	a_k	b_k	c_k	D	e_k	f_k	g_k	H
Sum	A_{1990}		D		E_{2019}		H	

$$Overall\ change = \frac{E_{2019} - A_{1990}}{A_{1990}}$$

$$a_i = b_i \times c_i \rightarrow A_{1990} = \sum a_i = \sum (b_i \times c_i)$$

$$e_i = f_i \times g_i \rightarrow E_{1990} = \sum e_i = \sum (f_i \times g_i)$$

First scenario: applying the age and sex structure and the age-specific rates of 1990 to the 2019 population:

Step 1: calculating the population size of each age group in 1990 by considering the 2019 population size and with the 1990 age structure:

$$\frac{c_i}{D} = \frac{X}{H} \rightarrow X = \hat{c}_i = \frac{c_i}{D} \times H$$

Step 2: calculating the hypothetical number of new cases in 1990 by applying the calculation in Step 1:

$$\hat{A}_{1990} = \sum (b_i \times \hat{c}_i)$$

Second scenario: Calculating the hypothetical number of new cases in 1990 by considering the 2019 population size and with the 2019 age structure:

$$\hat{A}_{age1990} = \sum (b_i \times g_i)$$

$$Overall\ change = \frac{E_{2019} - A_{1990}}{A_{1990}} = \frac{1}{A_{1990}} \times (\underbrace{E_{2019} - \hat{A}_{age1990}}_{\textit{Incidence rate change}} + \underbrace{\hat{A}_{age1990} - \hat{A}_{1990}}_{\textit{Age structure change}} + \underbrace{\hat{A}_{1990} - A_{1990}}_{\textit{Population growth}})$$

Supplementary table 1. Data sources used to generate breast cancer estimations in the North Africa and Middle East countries, for the period 1990-2019. (Extracted from: http://ghdx.healthdata.org/)

Country	Title	Time period covered	Coverage type	Data type	Provider
**Afghanistan**	Afghanistan Demographic and Health Survey 2015-2016	06/2015 to 02/2016	Country	Survey	Demographic and Health Surveys Program (DHS)
**Algeria**	Algeria - Algiers Cancer Registry Report 2004	01/2004 to 12/2004	Subnational	Disease registry	National Institute of Public Health (Algeria)
	Algeria - Algiers Cancer Registry Report 2006	01/2006 to 12/2006	Subnational	Disease registry	National Institute of Public Health (Algeria)
	Algeria - Algiers Cancer Registry Report 2007	01/2007 to 12/2007	Subnational	Disease registry	National Institute of Public Health (Algeria)
	Algeria - Batna Cancer Registy Report 2000-2006	01/2000 to 12/2006	Subnational	Disease registry	University Hospital of Batna
	Algeria - Oran Cancer Registry Report 2005	01/2005 to 12/2005	Subnational	Disease registry	Oran Cancer Registry (Algeria)
	Algeria - Oran Cancer Registry Report 2006	01/2006 to 12/2006	Subnational	Disease registry	Oran Cancer Registry (Algeria)
	Algeria - Setif Cancer Registry 1990-1993 - CI5	01/1990 to 12/1993		Disease registry	
	Algeria - Setif Cancer Registry 1998-2002 - CI5	01/1998 to 12/2002		Disease registry	
	Algeria - Setif Cancer Registry 2003-2007 - CI5	01/2003 to 12/2007		Disease registry	
	Algeria Tumour Registry of Algiers 1993-1997 - CI5	01/1993 to 12/1997		Disease registry	
**Bahrain**	Bahrain Cancer Registry 1998-2002 - CI5	01/1998 to 12/2002		Disease registry	
	Bahrain Cancer Registry 2003-2007 - CI5	01/2003 to 12/2007		Disease registry	
	Bahrain Health Statistics 2000	01/1996 to 12/2000	Country	Report	Ministry of Health (Bahrain)
**Egypt**	Egypt - Aswan National Cancer Registry Report 2008	01/2008 to 12/2008	Subnational	Disease registry	National Cancer Registry Program of Egypt
	Egypt - Aswan National Cancer Registry Statistics 2008	01/2008 to 12/2008	Subnational	Disease registry	National Cancer Registry Program of Egypt
	Egypt - Aswan National Cancer Registry Statistics 2009	01/2009 to 12/2009	Subnational	Disease registry	National Cancer Registry Program of Egypt
	Egypt - Aswan National Cancer Registry Statistics 2010	01/2010 to 12/2010	Subnational	Disease registry	National Cancer Registry Program of Egypt
	Egypt - Damietta National Cancer Registry Report 2009	01/2009 to 12/2009	Subnational	Disease registry	National Cancer Registry Program of Egypt
	Egypt - Damietta National Cancer Registry Statistics 2009	01/2009 to 12/2009	Subnational	Disease registry	National Cancer Registry Program of Egypt
	Egypt - Damietta National Cancer Registry Statistics 2010	01/2010 to 12/2010	Subnational	Disease registry	National Cancer Registry Program of Egypt
	Egypt - Damietta National Cancer Registry Statistics 2011	01/2011 to 12/2011	Subnational	Disease registry	National Cancer Registry Program of Egypt
	Egypt - Damietta National Cancer Registry Statistics 2012	01/2012 to 12/2012	Subnational	Disease registry	National Cancer Registry Program of Egypt
	Egypt - El-Minia National Cancer Registry Report 2009	01/2009 to 12/2009	Subnational	Disease registry	National Cancer Registry Program of Egypt

Country	Title	Time period covered	Coverage type	Data type	Provider
	Egypt - El-Minia National Cancer Registry Statistics 2009	01/2009 to 12/2009	Subnational	Disease registry	National Cancer Registry Program of Egypt
	Egypt - Gharbiah Cancer Registry 1999-2002 - CI5	01/1999 to 12/2002		Disease registry	
	Egypt - Gharbiah Cancer Registry 2003-2007 - CI5	01/2003 to 12/2007		Disease registry	
**Iran (Islamic Republic of)**	Cancer Occurrence in Ardabil: Results of a Population-Based Cancer Registry from Iran	01/1996 to 12/1999	Subnational	Scientific literature	
	Declining incidence of esophageal cancer in the Turkmen Plain, eastern part of the Caspian Littoral of Iran: A retrospective cancer surveillance	01/1996 to 12/2000	Subnational	Scientific literature	
	Iran - Golestan Cancer Registry 2005-2007 - CI5	01/2005 to 12/2007	Subnational	Disease registry	
	Iran - Golestan Cancer Registry Incidence Data 2006-2008	01/2006 to 12/2008	Subnational	Disease registry	
	Iran National Cancer Registry 2000	01/2000 to 12/2000	Country	Disease registry	
	Iran National Cancer Registry 2001	01/2001 to 12/2001	Country	Disease registry	
	Iran National Cancer Registry 2002	01/2002 to 12/2002	Country	Disease registry	
	Iran National Cancer Registry 2003	01/2003 to 12/2003	Country	Disease registry	
	Iran National Cancer Registry 2004	01/2004 to 12/2004	Country	Disease registry	
	Iran National Cancer Registry 2005	01/2005 to 12/2005	Country	Disease registry	
	Iran National Cancer Registry 2006	01/2006 to 12/2006	Country	Disease registry	
	Iran National Cancer Registry 2007	01/2007 to 12/2007	Country	Disease registry	
	Iran National Cancer Registry 2008	01/2008 to 12/2008	Country	Disease registry	
	Iran National Cancer Registry 2009	01/2009 to 12/2009	Country	Disease registry	
	Iran National Cancer Registry 2010	01/2010 to 12/2010	Country	Disease registry	
	Iran National Cancer Registry Report 2003-2004	03/2003 to 02/2004	Country	Disease registry	
	Iran National Cancer Registry Report 2004-2005	03/2004 to 02/2005	Country	Disease registry	
	Iran National Cancer Registry Report 2005-2006	03/2005 to 02/2006	Country	Disease registry	
	Iran National Cancer Registry Report 2006-2007	03/2006 to 02/2007	Country	Disease registry	
	Iran National Cancer Registry Report 2008-2009	03/2008 to 02/2009	Country	Disease registry	
	Iran National Cancer Registry Report 2009-2010	03/2009 to 03/2010	Country	Disease registry	
**Iraq**	Iraq Cancer Registry 2011	01/2011 to 12/2011	Country	Disease registry	
**Jordan**	Cancer incidence in Jordan, 1996-2005	01/1996 to 12/2005		Scientific literature	

Country	Title	Time period covered	Coverage type	Data type	Provider
	Jordan Cancer Incidence Report 2001	01/2001 to 12/2001	Country	Disease registry	Ministry of Health (Jordan)
	Jordan Cancer Incidence Report 2002	01/2002 to 12/2002	Country	Disease registry	Ministry of Health (Jordan)
	Jordan Cancer Incidence Report 2003	01/2003 to 12/2003	Country	Disease registry	Ministry of Health (Jordan)
	Jordan Cancer Incidence Report 2004	01/2004 to 12/2004	Country	Disease registry	Ministry of Health (Jordan)
	Jordan Cancer Incidence Report 2005	01/2005 to 12/2005	Country	Disease registry	Ministry of Health (Jordan)
	Jordan Cancer Incidence Report 2006	01/2005 to 12/2006	Country	Disease registry	Ministry of Health (Jordan)
	Jordan Cancer Incidence Report 2007	01/2006 to 12/2007	Country	Disease registry	Ministry of Health (Jordan)
	Jordan Cancer Incidence Report 2008	01/2007 to 12/2008	Country	Disease registry	Ministry of Health (Jordan)
	Jordan Cancer Incidence Report 2009	01/2008 to 12/2009	Country	Disease registry	Ministry of Health (Jordan)
	Jordan Cancer Incidence Report 2011	01/2011 to 12/2011	Country	Disease registry	Ministry of Health (Jordan)
	Jordan Cancer Incidence Report 2012	01/2012 to 12/2012	Country	Disease registry	Ministry of Health (Jordan)
	The Epidemiology and Trend of Cancer in Jordan, 2000-2013	01/2000 to 12/2013		Scientific literature	
**Kuwait**	Kuwait Cancer Registry 2003-2007 - CI5	01/2003 to 12/2007		Disease registry	
**Lebanon**	Cancer Incidence in Postwar Lebanon: Findings from the First National Population-based Registry, 1998	01/1998 to 12/1998		Scientific literature	
	Lebanon National Cancer Registry Tables 2005	01/2005 to 12/2005	Country	Disease registry	Ministry of Public Health (Lebanon)
	Lebanon National Cancer Registry Tables 2006	01/2006 to 12/2006	Country	Disease registry	Ministry of Public Health (Lebanon)
	Lebanon National Cancer Registry Tables 2007	01/2007 to 12/2007	Country	Disease registry	Ministry of Public Health (Lebanon)
	Lebanon Statistical Bulletin 2012	01/2008 to 12/2012	Country	Report	Ministry of Public Health (Lebanon)
	Lebanon Statistical Bulletin 2013	01/2007 to 12/2013	Country	Report	Ministry of Public Health (Lebanon)
	Lebanon Statistical Bulletin 2014	01/2008 to 12/2014	Country	Report	Ministry of Public Health (Lebanon)
	Lebanon Statistical Bulletin 2015	01/2010 to 12/2015	Country	Report	Ministry of Public Health (Lebanon)
	Lebanon Statistical Bulletin 2016	01/2011 to 12/2016	Country	Report	Ministry of Public Health (Lebanon)
	Lebanon Statistical Bulletin 2017	01/2011 to 12/2017	Country	Report	Ministry of Public Health (Lebanon)
**Libya**	Libya - Benghazi Cancer Registry 2003-2005 - CI5	01/2003 to 12/2005		Disease registry	
	Libya Family Health Survey 2007	05/2007 to 10/2007	Country	Survey	Pan Arab Project for Family Health (PAPFAM)
**Morocco**	Morocco - Cancer Registry of Greater Casablanca 2004	01/2004 to 12/2004	Subnational	Disease registry	Lalla Salma Association to Fight Against Cancer (Morocco)

Country	Title	Time period covered	Coverage type	Data type	Provider
	Morocco - Cancer Registry of Greater Casablanca Region 2005 2006 2007	01/2005 to 12/2007	Subnational	Disease registry	Lalla Salma Association to Fight Against Cancer (Morocco)
	Morocco Health in Figures 2014	01/1998 to 12/2014	Country	Report	Ministry of Health (Morocco)
**Oman**	Cancer Incidence in Oman 2013	01/2013 to 12/2013	Country	Disease registry	Ministry of Health (Oman)
	Oman - Cancer Incidence in Oman 2001	01/2001 to 12/2001	Country	Disease registry	Ministry of Health (Oman)
	Oman - Cancer Incidence in Oman 2002	01/2002 to 12/2002	Country	Disease registry	Ministry of Health (Oman)
	Oman - Cancer Incidence in Oman 2003	01/2003 to 12/2003	Country	Disease registry	Ministry of Health (Oman)
	Oman - Cancer Incidence in Oman 2004	01/2004 to 12/2004	Country	Disease registry	Ministry of Health (Oman)
	Oman - Cancer Incidence in Oman 2005	01/1996 to 12/2005	Country	Disease registry	Ministry of Health (Oman)
	Oman - Cancer Incidence in Oman 2006	01/1996 to 12/2006	Country	Disease registry	Ministry of Health (Oman)
	Oman - Cancer Incidence in Oman 2007	01/1997 to 12/2007	Country	Disease registry	Ministry of Health (Oman)
	Oman - Cancer Incidence in Oman 2008	01/2008 to 12/2008	Country	Disease registry	Ministry of Health (Oman)
	Oman - Cancer Incidence in Oman 2011	01/2011 to 12/2011	Country	Disease registry	Ministry of Health (Oman)
	Oman - Cancer Incidence in Oman 2012	01/2012 to 12/2012	Country	Disease registry	Ministry of Health (Oman)
	Oman - Cancer Incidence in Oman Report of 2009	01/1997 to 12/2009	Country	Disease registry	Ministry of Health (Oman)
	Oman - Cancer Incidence in Oman Report of 2010	01/1998 to 12/2010	Country	Disease registry	Ministry of Health (Oman)
	Oman Cancer Registry 1993-1997 - CI5	01/1993 to 12/1997		Disease registry	
	Oman Cancer Registry 1998-2001 - CI5	01/1998 to 12/2001		Disease registry	
**Palestine**	Palestine Health Annual Report 2018	01/2000 to 12/2018	Country	Report	Ministry of Health (Palestine)
	Palestine Health Annual Report 2019	01/2010 to 12/2019	Country	Report	Ministry of Health (Palestine)
	Palestine Health Annual Report 2020	01/2010 to 12/2020	Country	Report	Ministry of Health (Palestine)
	Palestine Health Status Annual Report 1999	01/1996 to 12/1999	Country	Report	Ministry of Health (Palestine)
	Palestine Health Status Annual Report 2000	01/2000 to 12/2000	Country	Report	Ministry of Health (Palestine)
	Palestine Health Status Annual Report 2001	01/1997 to 12/2001	Country	Report	Ministry of Health (Palestine)
	Palestine Health Status Annual Report 2010	01/2010 to 12/2010	Country	Report	Ministry of Health (Palestine)
	Palestine Health Status Annual Report 2011	01/2002 to 12/2011	Country	Report	Ministry of Health (Palestine)
	Palestine Health Status Annual Report 2012	01/2002 to 12/2012	Country	Report	Ministry of Health (Palestine)
	Palestine Health Status Annual Report 2013	01/2007 to 12/2013	Country	Report	Ministry of Health (Palestine)

Country	Title	Time period covered	Coverage type	Data type	Provider
	Palestine Health Status Annual Report 2014	01/2008 to 12/2014	Country	Report	Ministry of Health (Palestine)
	Palestine Health Status Annual Report 2015	01/2009 to 12/2015	Country	Report	Ministry of Health (Palestine)
	Palestine Health Status Annual Report 2016	01/2009 to 12/2016	Country	Report	Ministry of Health (Palestine)
	Palestine Multiple Indicator Cluster Survey 2010	05/2010 to 09/2010	Country	Survey	United Nations Children's Fund (UNICEF)
**Qatar**	Qatar Annual Health Report 2005	01/1996 to 12/2005	Country	Report	Hamad Medical Corporation (Qatar)
	Qatar Annual Health Report 2006	01/1997 to 12/2006	Country	Report	Hamad Medical Corporation (Qatar)
	Qatar Annual Health Report 2007	01/1998 to 12/2007	Country	Report	Hamad Medical Corporation (Qatar)
	Qatar Annual Health Report 2008	01/1998 to 12/2008	Country	Report	Hamad Medical Corporation (Qatar)
	Qatar Annual Health Report 2009	01/1999 to 12/2009	Country	Report	Hamad Medical Corporation (Qatar)
	Qatar Annual Health Report 2010	01/2000 to 12/2010	Country	Report	Hamad Medical Corporation (Qatar)
	Qatar Annual Health Report 2011	01/2003 to 12/2011	Country	Report	Hamad Medical Corporation (Qatar)
	Qatar Cancer Registry 2003-2007 - CI5	01/2003 to 12/2007		Disease registry	
	Qatar Vital Statistics Annual Bulletin 2014	01/2005 to 12/2014	Country	Vital registration	Ministry of Development Planning and Statistics (Qatar)
	Qatar Vital Statistics Annual Bulletin 2015	01/2006 to 12/2015	Country	Vital registration	Ministry of Development Planning and Statistics (Qatar)
	Qatar Vital Statistics Annual Bulletin 2016	01/2007 to 12/2016	Country	Vital registration	Ministry of Development Planning and Statistics (Qatar)
	Qatar Vital Statistics Annual Bulletin 2017	01/2007 to 12/2017	Country	Vital registration	Ministry of Development Planning and Statistics (Qatar)
	Qatar Vital Statistics Annual Bulletin 2018	01/2009 to 12/2018	Country	Vital registration	Ministry of Development Planning and Statistics (Qatar)
	Qatar World Health Survey 2006	04/2006 to 05/2006	Country	Survey	
**Saudi Arabia**	Saudi Arabia Cancer Incidence and Survival Report 2007	01/2007 to 12/2007	Country	Disease registry	Saudi Health Council
	Saudi Arabia Cancer Incidence Report 1994-1996	01/1994 to 12/1996	Country	Disease registry	Saudi Health Council
	Saudi Arabia Cancer Incidence Report 1997-1998	01/1997 to 12/1998	Country	Disease registry	Saudi Health Council
	Saudi Arabia Cancer Incidence Report 1999-2000	01/1999 to 12/2000	Country	Disease registry	Saudi Health Council
	Saudi Arabia Cancer Incidence Report 2001	01/2001 to 12/2001	Country	Disease registry	Saudi Health Council
	Saudi Arabia Cancer Incidence Report 2002	01/2002 to 12/2002	Country	Disease registry	Saudi Health Council
	Saudi Arabia Cancer Incidence Report 2003	01/2003 to 12/2003	Country	Disease registry	Saudi Health Council

Country	Title	Time period covered	Coverage type	Data type	Provider
	Saudi Arabia Cancer Incidence Report 2004	01/2004 to 12/2004	Country	Disease registry	Saudi Health Council
	Saudi Arabia Cancer Incidence Report 2005	01/2005 to 12/2005	Country	Disease registry	Saudi Health Council
	Saudi Arabia Cancer Incidence Report 2006	01/2006 to 12/2006	Country	Disease registry	Saudi Health Council
	Saudi Arabia Cancer Incidence Report 2008	01/2008 to 12/2008	Country	Disease registry	Saudi Health Council
	Saudi Arabia Cancer Incidence Report 2009	01/2009 to 12/2009	Country	Disease registry	Saudi Health Council
	Saudi Arabia Cancer Incidence Report 2010	01/2010 to 12/2010	Country	Disease registry	Saudi Health Council
	Saudi Arabia Cancer Incidence Report 2011	01/2011 to 12/2011	Country	Disease registry	Saudi Health Council
	Saudi Arabia Cancer Incidence Report 2012	01/2012 to 12/2012	Country	Disease registry	Saudi Health Council
	Saudi Arabia Health Interview Survey 2013	01/2013 to 12/2013	Country	Survey	
	Saudi Arabia Health Statistical Yearbook 1999	01/1994 to 12/1999	Country	Report	Ministry of Health (Saudi Arabia)
	Saudi Arabia Health Statistical Yearbook 2000	01/1991 to 12/2000	Country	Report	Ministry of Health (Saudi Arabia)
	Saudi Arabia Health Statistical Yearbook 2001	01/1996 to 12/2001	Country	Report	Ministry of Health (Saudi Arabia)
	Saudi Arabia Health Statistical Yearbook 2010	01/2006 to 12/2010	Country	Report	Ministry of Health (Saudi Arabia)
	Saudi Arabia Health Statistical Yearbook 2011	01/2007 to 12/2011	Country	Report	Ministry of Health (Saudi Arabia)
	Saudi Arabia Health Statistical Yearbook 2012	01/2008 to 12/2012	Country	Report	Ministry of Health (Saudi Arabia)
	Saudi Arabia Health Statistical Yearbook 2013	01/2007 to 12/2013	Country	Report	Ministry of Health (Saudi Arabia)
	Saudi Arabia Health Statistical Yearbook 2014	01/2005 to 12/2014	Country	Report	Ministry of Health (Saudi Arabia)
	Saudi Arabia Health Statistical Yearbook 2015	01/2006 to 12/2015	Country	Report	Ministry of Health (Saudi Arabia)
	Saudi Arabia Health Statistical Yearbook 2016	01/2007 to 12/2016	Country	Report	Ministry of Health (Saudi Arabia)
	Saudi Arabia Health Statistical Yearbook 2017	01/2013 to 12/2017	Country	Report	Ministry of Health (Saudi Arabia)
	Saudi Arabia Health Statistical Yearbook 2018	01/2014 to 12/2018	Country	Report	Ministry of Health (Saudi Arabia)
**Sudan**	Sudan Annual Health Statistics Report 2011	01/2007 to 12/2011	Country	Report	Federal Ministry of Health (Sudan)
**Syrian Arab Republic**					
**Tunisia**	Tunisia - Centre Sousse Cancer Registry 1998-2002 - CI5	01/1998 to 12/2002		Disease registry	
	Tunisia - North Cancer Registry 2003-2005 - CI5	01/2003 to 12/2005		Disease registry	
**Turkey**	Breast cancer risk factors in Turkish women -- a University Hospital based nested case control study	01/2000 to 12/2006	Subnational	Scientific literature	

Country	Title	Time period covered	Coverage type	Data type	Provider
	Risk factors for breast cancer in Turkish women: a hospital-based case-control study	01/2002 to 12/2003		Scientific literature	
	Turkey - Antalya Cancer Incidence 2004	01/2004 to 12/2004		Disease registry	
	Turkey - Antalya Cancer Incidence 2008	01/2008 to 12/2008	Subnational	Disease registry	
	Turkey - Antalya Cancer Registry 1998-2002 - CI5	01/1998 to 12/2002		Disease registry	
	Turkey - Izmir Cancer Incidence 2008	01/2008 to 12/2008	Subnational	Disease registry	
	Turkey - Izmir Cancer Registry 1998-2002 - CI5	01/1998 to 12/2002		Disease registry	
	Turkey - Izmir Cancer Registry Incidence 2006	01/2006 to 12/2006	Subnational	Disease registry	
	Turkey - Trabzon Cancer Registry 2005-2007 - CI5	01/2005 to 12/2007		Disease registry	
	Turkey Active Cancer Registration System 8 Provinces Incidence 2007	01/2007 to 12/2007	Subnational	Disease registry	
	Turkey Active Cancer Registration System 9 Provinces Incidence 2008	01/2008 to 12/2008	Subnational	Disease registry	
	Turkey Cancer Statistics 2002-2003	01/2002 to 12/2003	Subnational	Disease registry	Cancer Control Department, Ministry of Health (Turkey)
	Turkey Cancer Statistics 2004	01/2002 to 12/2004	Subnational	Disease registry	Cancer Control Department, Ministry of Health (Turkey)
	Turkey Cancer Statistics 2005	01/2005 to 12/2005	Subnational	Disease registry	Cancer Control Department, Ministry of Health (Turkey)
	Turkey Chronic Diseases and Risk Factors Study 2011	07/2011 to 09/2011	Country	Survey	Ministry of Health (Turkey)
	Turkey Health Statistics Yearbook 2008	Jan-08	Country	Report	Ministry of Health (Turkey)
	Turkey Health Statistics Yearbook 2009	01/2009 to 12/2009	Country	Report	Ministry of Health (Turkey)
	Turkey Health Statistics Yearbook 2010	01/2010 to 12/2010	Country	Report	Ministry of Health (Turkey)
	Turkey Health Statistics Yearbook 2014	01/2002 to 12/2014	Country	Report	Ministry of Health (Turkey)
	Turkey Health Statistics Yearbook 2015	01/2002 to 12/2015	Country	Report	Ministry of Health (Turkey)
	Turkey Health Statistics Yearbook 2016	01/2002 to 12/2016	Country	Report	Ministry of Health (Turkey)
	Turkey Health Statistics Yearbook 2017	01/2002 to 12/2017	Country	Report	Ministry of Health (Turkey)
	Turkey Statistical Yearbook 2011	01/1998 to 12/2011	Country	Report	Turkish Statistical Institute
**United Arab Emirates**	United Arab Emirates - Dubai Health Statistical Yearbook 2003	01/1993 to 12/2003	Subnational	Report	Dubai Department of Health and Medical Services

Country	Title	Time period covered	Coverage type	Data type	Provider
	United Arab Emirates - Dubai Health Statistical Yearbook 2004	01/1994 to 12/2004	Country	Report	Dubai Department of Health and Medical Services
	United Arab Emirates - Dubai Health Statistical Yearbook 2005	01/1995 to 12/2005	Subnational	Report	Dubai Department of Health and Medical Services
	United Arab Emirates - Dubai Health Statistical Yearbook 2006	01/1996 to 12/2006	Subnational	Report	Dubai Department of Health and Medical Services
	United Arab Emirates - Dubai Health Statistical Yearbook 2007	01/1997 to 12/2007	Subnational	Report	Dubai Department of Health and Medical Services
**Yemen**	Yemen Annual Statistical Health Report 2012	01/2012 to 12/2012	Country	Report	Ministry of Public Health and Population (Yemen)
	Yemen Annual Statistical Health Report 2013	01/2013 to 12/2013	Country	Report	Ministry of Public Health and Population (Yemen)
	Yemen Annual Statistical Health Report 2014	01/2014 to 12/2014	Country	Report	Ministry of Public Health and Population (Yemen)
	Yemen Cancer Incidence Report 2007-2011	01/2007 to 12/2011	Country	Disease registry	

Supplementary table 2. Relative risks used by age and sex for attributable burden estimation of each risk factors contributing to breast cancer. (Extracted from: Global burden of 87 risk factors in 204 countries and territories, 1990-2019: a systematic analysis for the Global Burden of Disease Study 2019. Lancet (London, England). 2020;396(10258):1223-49.)

Risk factor	Exposure	Mortality/Morbidity	Sex	All ages	0-6 days	7-27 days	28-384 days	5-9 years	10-14 years	15-19	20-24	25-29	30-34	35-39	40-44	45-49	50-54	55-59	60-64	65-69	70-74	75-79	80-84	85-89	90-94	95 +
**Secondhand smoke**	Exposed	Both	Female									1.072 (1.017 to 1.126)	1.072 (1.017 to 1.126)	1.072 (1.017 to 1.126)	1.072 (1.017 to 1.126)	1.072 (1.017 to 1.126)	1.072 (1.017 to 1.126)	1.072 (1.017 to 1.126)	1.072 (1.017 to 1.126)	1.072 (1.017 to 1.126)	1.072 (1.017 to 1.126)	1.072 (1.017 to 1.126)	1.072 (1.017 to 1.126)	1.072 (1.017 to 1.126)	1.072 (1.017 to 1.126)	1.072 (1.017 to 1.126)
	Not exposed	Both	Female									1 (1.0 to 1.0)	1 (1.0 to 1.0)	1 (1.0 to 1.0)	1 (1.0 to 1.0)	1 (1.0 to 1.0)	1 (1.0 to 1.0)	1 (1.0 to 1.0)	1 (1.0 to 1.0)	1 (1.0 to 1.0)	1 (1.0 to 1.0)	1 (1.0 to 1.0)	1 (1.0 to 1.0)	1 (1.0 to 1.0)	1 (1.0 to 1.0)	1 (1.0 to 1.0)
**Diet high in red meat**	0 g/day	Both	Both									1 (1.0 to 1.0)	1 (1.0 to 1.0)	1 (1.0 to 1.0)	1 (1.0 to 1.0)	1 (1.0 to 1.0)	1 (1.0 to 1.0)	1 (1.0 to 1.0)	1 (1.0 to 1.0)	1 (1.0 to 1.0)	1 (1.0 to 1.0)	1 (1.0 to 1.0)	1 (1.0 to 1.0)	1 (1.0 to 1.0)	1 (1.0 to 1.0)	1 (1.0 to 1.0)
	50 g/day	Both	Both									1.05 (1.03 to 1.07)	1.05 (1.03 to 1.07)	1.05 (1.03 to 1.07)	1.05 (1.03 to 1.07)	1.05 (1.03 to 1.07)	1.05 (1.03 to 1.07)	1.05 (1.03 to 1.07)	1.05 (1.03 to 1.07)	1.05 (1.03 to 1.07)	1.05 (1.03 to 1.07)	1.05 (1.03 to 1.07)	1.05 (1.03 to 1.07)	1.05 (1.03 to 1.07)	1.05 (1.03 to 1.07)	1.05 (1.03 to 1.07)
	100 g/day	Both	Both									1.07 (1.03 to 1.12)	1.07 (1.03 to 1.12)	1.07 (1.03 to 1.12)	1.07 (1.03 to 1.12)	1.07 (1.03 to 1.12)	1.07 (1.03 to 1.12)	1.07 (1.03 to 1.12)	1.07 (1.03 to 1.12)	1.07 (1.03 to 1.12)	1.07 (1.03 to 1.12)	1.07 (1.03 to 1.12)	1.07 (1.03 to 1.12)	1.07 (1.03 to 1.12)	1.07 (1.03 to 1.12)	1.07 (1.03 to 1.12)
	150 g/day	Both	Both									1.08 (1.03 to 1.18)	1.08 (1.03 to 1.18)	1.08 (1.03 to 1.18)	1.08 (1.03 to 1.18)	1.08 (1.03 to 1.18)	1.08 (1.03 to 1.18)	1.08 (1.03 to 1.18)	1.08 (1.03 to 1.18)	1.08 (1.03 to 1.18)	1.08 (1.03 to 1.18)	1.08 (1.03 to 1.18)	1.08 (1.03 to 1.18)	1.08 (1.03 to 1.18)	1.08 (1.03 to 1.18)	1.08 (1.03 to 1.18)
	200 g/day	Both	Both									1.1 (1.03 to 1.24)	1.1 (1.03 to 1.24)	1.1 (1.03 to 1.24)	1.1 (1.03 to 1.24)	1.1 (1.03 to 1.24)	1.1 (1.03 to 1.24)	1.1 (1.03 to 1.24)	1.1 (1.03 to 1.24)	1.1 (1.03 to 1.24)	1.1 (1.03 to 1.24)	1.1 (1.03 to 1.24)	1.1 (1.03 to 1.24)	1.1 (1.03 to 1.24)	1.1 (1.03 to 1.24)	1.1 (1.03 to 1.24)
**Low physical activity**	0 METs	Both	Both	1.0 (1.0 to 1.0)																						
	1200 METs	Both	Both	0.974 (0.942 to 1.005)																						
	1800 METs	Both	Both	0.96 (0.914 to 1.008)																						

Risk factor	Exposure	Mortality/Morbidity	Sex	All ages	0-6 days	7-27 days	28-384 days	5-9 years	10-14 years	15-19	20-24	25-29	30-34	35-39	40-44	45-49	50-54	55-59	60-64	65-69	70-74	75-79	80-84	85-89	90-94	95 +
	2400 METs	Both	Both	0.957 (0.914 to 1.001)																						
	3000 METs	Both	Both	0.953 (0.913 to 0.994)																						
	3600 METs	Both	Both	0.949 (0.912 to 0.99)																						
	4200 METs	Both	Both	0.946 (0.909 to 0.985)																						
	600 METs	Both	Both	0.987 (0.971 to 1.003)																						
**High fasting plasma glucose (categorical)**	Diabetic	Both	Both									1.513 (1.087 to 2.206)	1.513 (1.087 to 2.206)	1.513 (1.087 to 2.206)	1.513 (1.087 to 2.206)	1.513 (1.087 to 2.206)	1.513 (1.087 to 2.206)	1.513 (1.087 to 2.206)	1.513 (1.087 to 2.206)	1.513 (1.087 to 2.206)	1.513 (1.087 to 2.206)	1.513 (1.087 to 2.206)	1.513 (1.087 to 2.206)	1.513 (1.087 to 2.206)	1.513 (1.087 to 2.206)	1.513 (1.087 to 2.206)
	Not diabetic	Both	Both									1.0 (1.0 to 1.0)	1.0 (1.0 to 1.0)	1.0 (1.0 to 1.0)	1.0 (1.0 to 1.0)	1.0 (1.0 to 1.0)	1.0 (1.0 to 1.0)	1.0 (1.0 to 1.0)	1.0 (1.0 to 1.0)	1.0 (1.0 to 1.0)	1.0 (1.0 to 1.0)	1.0 (1.0 to 1.0)	1.0 (1.0 to 1.0)	1.0 (1.0 to 1.0)	1.0 (1.0 to 1.0)	1.0 (1.0 to 1.0)
**High body-mass index in adults**	5 kg/m2	Both	Female								0.89 (0.869 to 0.914)	0.89 (0.869 to 0.914)	0.89 (0.869 to 0.914)	0.89 (0.869 to 0.914)	0.89 (0.869 to 0.914)	0.89 (0.869 to 0.914)	1.089 (1.037 to 1.14)	1.089 (1.037 to 1.14)	1.089 (1.037 to 1.14)	1.089 (1.037 to 1.14)	1.089 (1.037 to 1.14)	1.089 (1.037 to 1.14)	1.089 (1.037 to 1.14)	1.089 (1.037 to 1.14)	1.089 (1.037 to 1.14)	1.089 (1.037 to 1.14)
**Alcohol use**	72 g/day	Both	Both	1.476 (1.282 to 1.691)																						
	60 g/day	Both	Both	1.452 (1.312 to 1.599)																						
	48 g/day	Both	Both	1.443 (1.348 to 1.542)																						

Risk factor	Exposure	Mortality/Morbidity	Sex	All ages	0-6 days	7-27 days	28-384 days	5-9 years	10-14 years	15-19	20-24	25-29	30-34	35-39	40-44	45-49	50-54	55-59	60-64	65-69	70-74	75-79	80-84	85-89	90-94	95 +
	36 g/day	Both	Both	1.433 (1.311 to 1.551)																						
	24 g/day	Both	Both	1.329 (1.237 to 1.419)																						
	12 g/day	Both	Both	1.17 (1.081 to 1.265)																						
	0 g/day	Both	Both	1.0 (1.0 to 1.0)																						
**Smoking**	0 Pack Years	Both	Female										1.0 (1.0 to 1.0)	1.0 (1.0 to 1.0)	1.0 (1.0 to 1.0)	1.0 (1.0 to 1.0)	1.0 (1.0 to 1.0)	1.0 (1.0 to 1.0)	1.0 (1.0 to 1.0)	1.0 (1.0 to 1.0)	1.0 (1.0 to 1.0)	1.0 (1.0 to 1.0)	1.0 (1.0 to 1.0)	1.0 (1.0 to 1.0)	1.0 (1.0 to 1.0)	1.0 (1.0 to 1.0)
	0 Pack Years	Both	Male										1.0 (1.0 to 1.0)	1.0 (1.0 to 1.0)	1.0 (1.0 to 1.0)	1.0 (1.0 to 1.0)	1.0 (1.0 to 1.0)	1.0 (1.0 to 1.0)	1.0 (1.0 to 1.0)	1.0 (1.0 to 1.0)	1.0 (1.0 to 1.0)	1.0 (1.0 to 1.0)	1.0 (1.0 to 1.0)	1.0 (1.0 to 1.0)	1.0 (1.0 to 1.0)	1.0 (1.0 to 1.0)
	10 Pack Years	Both	Female										1.16 (1.09 to 1.24)	1.16 (1.09 to 1.24)	1.16 (1.09 to 1.24)	1.16 (1.09 to 1.24)	1.16 (1.09 to 1.24)	1.16 (1.09 to 1.24)	1.16 (1.09 to 1.24)	1.16 (1.09 to 1.24)	1.16 (1.09 to 1.24)	1.16 (1.09 to 1.24)	1.16 (1.09 to 1.24)	1.16 (1.09 to 1.24)	1.16 (1.09 to 1.24)	1.16 (1.09 to 1.24)
	10 Pack Years	Both	Male										1.0 (1.0 to 1.0)	1.0 (1.0 to 1.0)	1.0 (1.0 to 1.0)	1.0 (1.0 to 1.0)	1.0 (1.0 to 1.0)	1.0 (1.0 to 1.0)	1.0 (1.0 to 1.0)	1.0 (1.0 to 1.0)	1.0 (1.0 to 1.0)	1.0 (1.0 to 1.0)	1.0 (1.0 to 1.0)	1.0 (1.0 to 1.0)	1.0 (1.0 to 1.0)	1.0 (1.0 to 1.0)
	20 Pack Years	Both	Female										1.27 (1.18 to 1.36)	1.27 (1.18 to 1.36)	1.27 (1.18 to 1.36)	1.27 (1.18 to 1.36)	1.27 (1.18 to 1.36)	1.27 (1.18 to 1.36)	1.27 (1.18 to 1.36)	1.27 (1.18 to 1.36)	1.27 (1.18 to 1.36)	1.27 (1.18 to 1.36)	1.27 (1.18 to 1.36)	1.27 (1.18 to 1.36)	1.27 (1.18 to 1.36)	1.27 (1.18 to 1.36)
	20 Pack Years	Both	Male										1.0 (1.0 to 1.0)	1.0 (1.0 to 1.0)	1.0 (1.0 to 1.0)	1.0 (1.0 to 1.0)	1.0 (1.0 to 1.0)	1.0 (1.0 to 1.0)	1.0 (1.0 to 1.0)	1.0 (1.0 to 1.0)	1.0 (1.0 to 1.0)	1.0 (1.0 to 1.0)	1.0 (1.0 to 1.0)	1.0 (1.0 to 1.0)	1.0 (1.0 to 1.0)	1.0 (1.0 to 1.0)
	30 Pack Years	Both	Female										1.29 (1.19 to 1.39)	1.29 (1.19 to 1.39)	1.29 (1.19 to 1.39)	1.29 (1.19 to 1.39)	1.29 (1.19 to 1.39)	1.29 (1.19 to 1.39)	1.29 (1.19 to 1.39)	1.29 (1.19 to 1.39)	1.29 (1.19 to 1.39)	1.29 (1.19 to 1.39)	1.29 (1.19 to 1.39)	1.29 (1.19 to 1.39)	1.29 (1.19 to 1.39)	1.29 (1.19 to 1.39)
	30 Pack Years	Both	Male										1.0 (1.0 to 1.0)	1.0 (1.0 to 1.0)	1.0 (1.0 to 1.0)	1.0 (1.0 to 1.0)	1.0 (1.0 to 1.0)	1.0 (1.0 to 1.0)	1.0 (1.0 to 1.0)	1.0 (1.0 to 1.0)	1.0 (1.0 to 1.0)	1.0 (1.0 to 1.0)	1.0 (1.0 to 1.0)	1.0 (1.0 to 1.0)	1.0 (1.0 to 1.0)	1.0 (1.0 to 1.0)

Risk factor	Exposure	Mortality/Morbidity	Sex	All ages	0-6 days	7-27 days	28-384 days	5-9 years	10-14 years	15-19	20-24	25-29	30-34	35-39	40-44	45-49	50-54	55-59	60-64	65-69	70-74	75-79	80-84	85-89	90-94	95 +
	40 Pack Years	Both	Female										1.24 (1.12 to 1.37)	1.24 (1.12 to 1.37)	1.24 (1.12 to 1.37)	1.24 (1.12 to 1.37)	1.24 (1.12 to 1.37)	1.24 (1.12 to 1.37)	1.24 (1.12 to 1.37)	1.24 (1.12 to 1.37)	1.24 (1.12 to 1.37)	1.24 (1.12 to 1.37)	1.24 (1.12 to 1.37)	1.24 (1.12 to 1.37)	1.24 (1.12 to 1.37)	1.24 (1.12 to 1.37)
	40 Pack Years	Both	Male										1.0 (1.0 to 1.0)	1.0 (1.0 to 1.0)	1.0 (1.0 to 1.0)	1.0 (1.0 to 1.0)	1.0 (1.0 to 1.0)	1.0 (1.0 to 1.0)	1.0 (1.0 to 1.0)	1.0 (1.0 to 1.0)	1.0 (1.0 to 1.0)	1.0 (1.0 to 1.0)	1.0 (1.0 to 1.0)	1.0 (1.0 to 1.0)	1.0 (1.0 to 1.0)	1.0 (1.0 to 1.0)
	50 Pack Years	Both	Female										1.26 (1.11 to 1.40)	1.26 (1.11 to 1.40)	1.26 (1.11 to 1.40)	1.26 (1.11 to 1.40)	1.26 (1.11 to 1.40)	1.26 (1.11 to 1.40)	1.26 (1.11 to 1.40)	1.26 (1.11 to 1.40)	1.26 (1.11 to 1.40)	1.26 (1.11 to 1.40)	1.26 (1.11 to 1.40)	1.26 (1.11 to 1.40)	1.26 (1.11 to 1.40)	1.26 (1.11 to 1.40)
	50 Pack Years	Both	Male										1.0 (1.0 to 1.0)	1.0 (1.0 to 1.0)	1.0 (1.0 to 1.0)	1.0 (1.0 to 1.0)	1.0 (1.0 to 1.0)	1.0 (1.0 to 1.0)	1.0 (1.0 to 1.0)	1.0 (1.0 to 1.0)	1.0 (1.0 to 1.0)	1.0 (1.0 to 1.0)	1.0 (1.0 to 1.0)	1.0 (1.0 to 1.0)	1.0 (1.0 to 1.0)	1.0 (1.0 to 1.0)
	60 Pack Years	Both	Female										1.27 (1.11 to 1.44)	1.27 (1.11 to 1.44)	1.27 (1.11 to 1.44)	1.27 (1.11 to 1.44)	1.27 (1.11 to 1.44)	1.27 (1.11 to 1.44)	1.27 (1.11 to 1.44)	1.27 (1.11 to 1.44)	1.27 (1.11 to 1.44)	1.27 (1.11 to 1.44)	1.27 (1.11 to 1.44)	1.27 (1.11 to 1.44)	1.27 (1.11 to 1.44)	1.27 (1.11 to 1.44)
	60 Pack Years	Both	Male										1.0 (1.0 to 1.0)	1.0 (1.0 to 1.0)	1.0 (1.0 to 1.0)	1.0 (1.0 to 1.0)	1.0 (1.0 to 1.0)	1.0 (1.0 to 1.0)	1.0 (1.0 to 1.0)	1.0 (1.0 to 1.0)	1.0 (1.0 to 1.0)	1.0 (1.0 to 1.0)	1.0 (1.0 to 1.0)	1.0 (1.0 to 1.0)	1.0 (1.0 to 1.0)	1.0 (1.0 to 1.0)
	0 Years Since Quitting	Both	Female										100.00 (100.00 to 100.00)	100.00 (100.00 to 100.00)	100.00 (100.00 to 100.00)	100.00 (100.00 to 100.00)	100.00 (100.00 to 100.00)	100.00 (100.00 to 100.00)	100.00 (100.00 to 100.00)	100.00 (100.00 to 100.00)	100.00 (100.00 to 100.00)	100.00 (100.00 to 100.00)	100.00 (100.00 to 100.00)	100.00 (100.00 to 100.00)	100.00 (100.00 to 100.00)	100.00 (100.00 to 100.00)
	0 Years Since Quitting	Both	Male										0.00 (0.00 to 0.00)	0.00 (0.00 to 0.00)	0.00 (0.00 to 0.00)	0.00 (0.00 to 0.00)	0.00 (0.00 to 0.00)	0.00 (0.00 to 0.00)	0.00 (0.00 to 0.00)	0.00 (0.00 to 0.00)	0.00 (0.00 to 0.00)	0.00 (0.00 to 0.00)	0.00 (0.00 to 0.00)	0.00 (0.00 to 0.00)	0.00 (0.00 to 0.00)	0.00 (0.00 to 0.00)
	10 Years Since Quitting	Both	Female										55.99 (44.97 to 69.74)	55.99 (44.97 to 69.74)	55.99 (44.97 to 69.74)	55.99 (44.97 to 69.74)	55.99 (44.97 to 69.74)	55.99 (44.97 to 69.74)	55.99 (44.97 to 69.74)	55.99 (44.97 to 69.74)	55.99 (44.97 to 69.74)	55.99 (44.97 to 69.74)	55.99 (44.97 to 69.74)	55.99 (44.97 to 69.74)	55.99 (44.97 to 69.74)	55.99 (44.97 to 69.74)
	10 Years Since Quitting	Both	Male										0.00 (0.00 to 0.00)	0.00 (0.00 to 0.00)	0.00 (0.00 to 0.00)	0.00 (0.00 to 0.00)	0.00 (0.00 to 0.00)	0.00 (0.00 to 0.00)	0.00 (0.00 to 0.00)	0.00 (0.00 to 0.00)	0.00 (0.00 to 0.00)	0.00 (0.00 to 0.00)	0.00 (0.00 to 0.00)	0.00 (0.00 to 0.00)	0.00 (0.00 to 0.00)	0.00 (0.00 to 0.00)
	20 Years Since Quitting	Both	Female										41.34 (33.50 to 49.65)	41.34 (33.50 to 49.65)	41.34 (33.50 to 49.65)	41.34 (33.50 to 49.65)	41.34 (33.50 to 49.65)	41.34 (33.50 to 49.65)	41.34 (33.50 to 49.65)	41.34 (33.50 to 49.65)	41.34 (33.50 to 49.65)	41.34 (33.50 to 49.65)	41.34 (33.50 to 49.65)	41.34 (33.50 to 49.65)	41.34 (33.50 to 49.65)	41.34 (33.50 to 49.65)

Risk factor	Exposure	Mortality/Morbidity	Sex	All ages	0-6 days	7-27 days	28-384 days	5-9 years	10-14 years	15-19	20-24	25-29	30-34	35-39	40-44	45-49	50-54	55-59	60-64	65-69	70-74	75-79	80-84	85-89	90-94	95 +
	20 Years Since Quitting	Both	Male										0.00 (0.00 to 0.00)	0.00 (0.00 to 0.00)	0.00 (0.00 to 0.00)	0.00 (0.00 to 0.00)	0.00 (0.00 to 0.00)	0.00 (0.00 to 0.00)	0.00 (0.00 to 0.00)	0.00 (0.00 to 0.00)	0.00 (0.00 to 0.00)	0.00 (0.00 to 0.00)	0.00 (0.00 to 0.00)	0.00 (0.00 to 0.00)	0.00 (0.00 to 0.00)	0.00 (0.00 to 0.00)
	30 Years Since Quitting	Both	Female										30.49 (20.15 to 40.26)	30.49 (20.15 to 40.26)	30.49 (20.15 to 40.26)	30.49 (20.15 to 40.26)	30.49 (20.15 to 40.26)	30.49 (20.15 to 40.26)	30.49 (20.15 to 40.26)	30.49 (20.15 to 40.26)	30.49 (20.15 to 40.26)	30.49 (20.15 to 40.26)	30.49 (20.15 to 40.26)	30.49 (20.15 to 40.26)	30.49 (20.15 to 40.26)	30.49 (20.15 to 40.26)
	30 Years Since Quitting	Both	Male										0.00 (0.00 to 0.00)	0.00 (0.00 to 0.00)	0.00 (0.00 to 0.00)	0.00 (0.00 to 0.00)	0.00 (0.00 to 0.00)	0.00 (0.00 to 0.00)	0.00 (0.00 to 0.00)	0.00 (0.00 to 0.00)	0.00 (0.00 to 0.00)	0.00 (0.00 to 0.00)	0.00 (0.00 to 0.00)	0.00 (0.00 to 0.00)	0.00 (0.00 to 0.00)	0.00 (0.00 to 0.00)
	40 Years Since Quitting	Both	Female										18.29 (6.10 to 30.76)	18.29 (6.10 to 30.76)	18.29 (6.10 to 30.76)	18.29 (6.10 to 30.76)	18.29 (6.10 to 30.76)	18.29 (6.10 to 30.76)	18.29 (6.10 to 30.76)	18.29 (6.10 to 30.76)	18.29 (6.10 to 30.76)	18.29 (6.10 to 30.76)	18.29 (6.10 to 30.76)	18.29 (6.10 to 30.76)	18.29 (6.10 to 30.76)	18.29 (6.10 to 30.76)
	40 Years Since Quitting	Both	Male										0.00 (0.00 to 0.00)	0.00 (0.00 to 0.00)	0.00 (0.00 to 0.00)	0.00 (0.00 to 0.00)	0.00 (0.00 to 0.00)	0.00 (0.00 to 0.00)	0.00 (0.00 to 0.00)	0.00 (0.00 to 0.00)	0.00 (0.00 to 0.00)	0.00 (0.00 to 0.00)	0.00 (0.00 to 0.00)	0.00 (0.00 to 0.00)	0.00 (0.00 to 0.00)	0.00 (0.00 to 0.00)

Supplementary table 3. The estimated socio-demographic index (SDI) values for the NAME region and included countries in this study for the investigated time points in the analysis of results.

	SDI			
**Location**	**1990**	**2000**	**2010**	**2019**
Afghanistan	0.187	0.188	0.264	0.343
Algeria	0.436	0.526	0.599	0.652
Bahrain	0.553	0.626	0.711	0.751
Egypt	0.403	0.504	0.582	0.658
Iran (Islamic Republic of)	0.404	0.517	0.622	0.67
Iraq	0.392	0.462	0.57	0.671
Jordan	0.52	0.6	0.681	0.731
Kuwait	0.655	0.724	0.801	0.851
Lebanon	0.462	0.548	0.639	0.708
Libya	0.405	0.566	0.691	0.709
Morocco	0.347	0.409	0.475	0.548
North Africa and Middle East	0.414	0.506	0.595	0.66
Oman	0.441	0.588	0.715	0.783
Palestine	0.314	0.407	0.497	0.588
Qatar	0.585	0.694	0.772	0.83
Saudi Arabia	0.48	0.602	0.726	0.805
Sudan	0.227	0.308	0.416	0.515
Syrian Arab Republic	0.367	0.476	0.594	0.619
Tunisia	0.434	0.538	0.622	0.672
Turkey	0.473	0.577	0.68	0.748
United Arab Emirates	0.621	0.762	0.853	0.88
Yemen	0.176	0.263	0.366	0.412

Supplementary table 4. The estimated Healthcare Access and Quality (HAQ) Index for the North Africa and Middle East countries, based on the Global Burden of Disease Study 2019. *(Reference: Assessing performance on the Healthcare Access and Quality Index by age group for 204 countries and territories, 1990–2019: a systematic analysis from the Global Burden of Disease Study 2019 [Under Review])*

	**Healthcare Access and Quality Index**	
**Location**	**1990**	**2019**
Afghanistan	13 (12.2 to 13.9)	26.9 (25.4 to 28.4)
Algeria	43.9 (43 to 44.9)	66 (64.4 to 67.5)
Bahrain	47.7 (46.8 to 48.6)	74.5 (72.9 to 76)
Egypt	33.5 (32.5 to 34.4)	57 (55.4 to 58.7)
Iran (Islamic Republic of)	47.8 (46.8 to 48.8)	70.9 (69.3 to 72.5)
Iraq	40.9 (39.9 to 41.9)	61.9 (60.2 to 63.5)
Jordan	45.5 (44.5 to 46.5)	70.8 (69.3 to 72.4)
Kuwait	65 (64.1 to 66)	82.5 (81.2 to 83.9)
Lebanon	50.6 (49.8 to 51.6)	81.1 (79.8 to 82.3)
Libya	47.4 (46.4 to 48.3)	63.1 (61.4 to 64.7)
Morocco	32.2 (31.3 to 33.1)	54.9 (53.1 to 56.7)
Oman	49.9 (48.9 to 50.8)	76.1 (74.7 to 77.6)
Palestine	46.9 (45.9 to 47.8)	63.3 (61.7 to 65)
Qatar	50.8 (49.9 to 51.8)	81.2 (79.9 to 82.5)
Saudi Arabia	45.7 (44.8 to 46.7)	74.3 (72.8 to 75.8)
Sudan	26 (25.2 to 27)	47.2 (45.3 to 48.8)
Syrian Arab Republic	41.9 (41 to 42.7)	65.3 (63.7 to 67)
Tunisia	49.8 (48.9 to 50.7)	73.2 (71.6 to 74.6)
Turkey	39.9 (38.9 to 40.9)	73.5 (72 to 74.9)
United Arab Emirates	44.5 (43.6 to 45.4)	61.2 (59.5 to 62.7)
Yemen	27 (26.1 to 27.9)	42.9 (41.2 to 44.6)

Supplementary table 5. Incidence, prevalence, deaths, YLLs (Years of Life Lost), YLDs (Years Lived with Disability), DALYs (Disability-Adjusted Life Years) of breast cancer by sex in 21 countries of the North Africa and Middle East region, in 1990 and 2019.

		1990												2019											
		Incidence		Prevalence		Deaths		YLLs (Years of Life Lost)		YLDs (Years Lived with Disability)		DALYs (Disability-Adjusted Life Years)		Incidence		Prevalence		Deaths		YLLs (Years of Life Lost)		YLDs (Years Lived with Disability)		DALYs (Disability-Adjusted Life Years)	
**Location**	**Sex**	**Number**	**Rate**	**Number**	**Rate**	**Number**	**Rate**	**Number**	**Rate**	**Number**	**Rate**	**Number**	**Rate**	**Number**	**Rate**	**Number**	**Rate**	**Number**	**Rate**	**Number**	**Rate**	**Number**	**Rate**	**Number**	**Rate**
**Afghanistan**	Female	644 (497 to 829)	17.1 (13.4 to 21.7)	4693 (3835 to 5734)	124.9 (102.8 to 151.6)	501 (389 to 642)	13.9 (11 to 17.5)	17181 (13140 to 22140)	437 (336 to 561.2)	374 (248 to 547)	9.8 (6.6 to 14.2)	17555 (13344 to 22678)	446.8 (342.1 to 573.5)	1951 (1451 to 2561)	22.3 (16.8 to 29.1)	13823 (10691 to 17817)	153.1 (121.8 to 192.8)	1281 (964 to 1673)	16.5 (12.5 to 21.3)	47124 (35205 to 62592)	493.7 (370.6 to 648.3)	1134 (729 to 1655)	12.5 (8.2 to 17.8)	48258 (36046 to 63895)	506.2 (380.9 to 663.4)
	Male	28 (13 to 52)	0.8 (0.4 to 1.4)	142 (77 to 252)	3.8 (2.2 to 6.5)	25 (13 to 47)	0.7 (0.4 to 1.3)	674 (317 to 1272)	17.7 (8.5 to 32.9)	16 (7 to 30)	0.4 (0.2 to 0.8)	690 (324 to 1301)	18.1 (8.7 to 33.6)	37 (21 to 61)	0.6 (0.3 to 0.9)	229 (144 to 361)	3.3 (2.2 to 4.9)	30 (17 to 49)	0.5 (0.3 to 0.8)	930 (510 to 1578)	12.1 (7.1 to 19.4)	23 (12 to 39)	0.3 (0.2 to 0.6)	953 (526 to 1615)	12.4 (7.4 to 19.9)
**Algeria**	Female	1526 (1186 to 1937)	22.1 (17.5 to 27.9)	14307 (11660 to 17462)	206.6 (171 to 249.7)	829 (657 to 1045)	13.2 (10.8 to 16.4)	28701 (22421 to 36735)	396.8 (311.9 to 505)	1024 (669 to 1513)	14.6 (9.6 to 21.2)	29725 (23246 to 37929)	411.4 (324.4 to 522.1)	6621 (4954 to 8490)	34 (25.6 to 43.3)	60497 (47142 to 75557)	311.4 (248.2 to 384.3)	2407 (1823 to 3047)	13.9 (10.7 to 17.3)	78779 (58625 to 101604)	396.6 (296.9 to 507)	4445 (2884 to 6539)	22.6 (14.8 to 32.8)	83225 (62174 to 107206)	419.3 (315.9 to 537.8)
	Male	195 (138 to 262)	4 (2.9 to 5.4)	1145 (841 to 1520)	19.9 (14.8 to 26.3)	152 (108 to 203)	3.6 (2.6 to 4.9)	3518 (2552 to 4770)	63.4 (45.4 to 84)	114 (69 to 172)	2.1 (1.3 to 3.1)	3632 (2629 to 4926)	65.5 (46.8 to 86.8)	430 (272 to 630)	2.7 (1.7 to 3.8)	3088 (2045 to 4388)	18.1 (12.2 to 25.6)	254 (166 to 364)	1.7 (1.1 to 2.5)	5646 (3677 to 7999)	32.9 (21.4 to 46.4)	285 (164 to 441)	1.7 (1 to 2.6)	5931 (3858 to 8439)	34.6 (22.5 to 48.9)
**Bahrain**	Female	47 (39 to 56)	45.8 (38.9 to 54)	388 (329 to 453)	373.4 (322.9 to 433.4)	24 (21 to 29)	27.4 (23.5 to 31.8)	830 (698 to 979)	760.4 (646.3 to 891.3)	30 (20 to 42)	28.2 (19.5 to 38.9)	859 (721 to 1013)	788.6 (671 to 925.1)	346 (271 to 433)	67.5 (54 to 83.1)	3093 (2507 to 3783)	591.5 (492.6 to 708.6)	103 (82 to 127)	25.2 (20.4 to 30.5)	3362 (2676 to 4186)	624.6 (500.5 to 769)	229 (151 to 331)	43.7 (29.7 to 61.3)	3592 (2857 to 4487)	668.3 (533.3 to 827.7)
	Male	0 (0 to 0)	0.2 (0.1 to 0.3)	2 (2 to 3)	1.8 (1.4 to 2.3)	0 (0 to 0)	0.1 (0.1 to 0.2)	4 (3 to 5)	3.2 (2.3 to 4.4)	0 (0 to 0)	0.1 (0.1 to 0.2)	4 (3 to 6)	3.4 (2.4 to 4.6)	3 (2 to 4)	0.5 (0.3 to 0.7)	23 (16 to 31)	3.7 (2.5 to 5.2)	1 (1 to 2)	0.2 (0.1 to 0.3)	31 (20 to 44)	4.7 (3 to 6.8)	2 (1 to 3)	0.3 (0.2 to 0.6)	33 (21 to 47)	5.1 (3.2 to 7.3)
**Egypt**	Female	2509 (2285 to 2749)	14.2 (13 to 15.5)	23599 (21009 to 26486)	140.8 (125.7 to 159.8)	1581 (1450 to 1723)	9.6 (8.8 to 10.4)	55395 (50382 to 60797)	300.3 (275 to 328.1)	1677 (1160 to 2316)	9.6 (6.6 to 13.2)	57072 (51856 to 62539)	309.9 (283.4 to 338.1)	10600 (7356 to 14525)	29.3 (20.1 to 40)	93258 (69133 to 122756)	262.8 (199.5 to 339.5)	4650 (3159 to 6344)	14.2 (9.6 to 19.2)	156108 (107927 to 215267)	417 (286.2 to 570.2)	6981 (4254 to 10451)	19.3 (11.9 to 28.4)	163089 (112703 to 223017)	436.2 (299.4 to 592)
	Male	10 (7 to 12)	0.1 (0 to 0.1)	94 (71 to 120)	0.6 (0.5 to 0.8)	7 (6 to 9)	0.1 (0 to 0.1)	229 (179 to 281)	1.3 (1 to 1.6)	7 (5 to 11)	0 (0 to 0.1)	237 (186 to 290)	1.3 (1 to 1.7)	37 (22 to 54)	0.1 (0.1 to 0.1)	319 (221 to 445)	0.9 (0.6 to 1.2)	22 (13 to 32)	0.1 (0 to 0.1)	625 (382 to 918)	1.6 (1 to 2.3)	28 (16 to 46)	0.1 (0 to 0.1)	653 (399 to 961)	1.7 (1 to 2.4)
**Iran (Islamic Republic of)**	Female	2835 (2352 to 3575)	18.8 (15.3 to 24.1)	26160 (22174 to 31486)	178.6 (149.7 to 216.4)	1413 (1160 to 1796)	10.3 (8.2 to 13.6)	48016 (40680 to 59999)	307.6 (255.7 to 386.4)	1903 (1285 to 2739)	12.6 (8.5 to 18.4)	49919 (42208 to 62170)	320.2 (265.4 to 405.4)	14743 (13248 to 16469)	34 (30.7 to 37.9)	134187 (120955 to 149206)	312.1 (281.5 to 345.4)	4704 (4306 to 5192)	11.9 (10.8 to 13.1)	151570 (138503 to 166910)	345.9 (316.9 to 380.9)	9916 (6887 to 13657)	22.8 (15.9 to 31.4)	161486 (147227 to 177500)	368.7 (336.7 to 404.3)
	Male	36 (27 to 48)	0.2 (0.2 to 0.3)	298 (233 to 383)	2 (1.6 to 2.6)	23 (18 to 30)	0.2 (0.1 to 0.2)	702 (550 to 895)	4.3 (3.4 to 5.6)	26 (17 to 38)	0.2 (0.1 to 0.3)	728 (574 to 926)	4.5 (3.5 to 5.8)	120 (98 to 143)	0.3 (0.3 to 0.4)	1070 (886 to 1280)	2.8 (2.3 to 3.3)	56 (48 to 63)	0.2 (0.1 to 0.2)	1512 (1296 to 1689)	3.8 (3.2 to 4.2)	93 (62 to 133)	0.2 (0.2 to 0.3)	1605 (1379 to 1790)	4 (3.5 to 4.5)
**Iraq**	Female	1337 (970 to 1824)	29 (21 to 40)	10887 (8421 to 14073)	239.4 (186 to 307.6)	762 (548 to 1046)	17.3 (12.5 to 23.8)	26651 (19302 to 36443)	565.4 (406.8 to 773.4)	833 (520 to 1258)	18 (11 to 27.2)	27485 (19934 to 37671)	583.4 (421.5 to 798.3)	7819 (5733 to 10484)	52 (38.9 to 68.9)	67317 (51236 to 88245)	451.6 (351.6 to 583.5)	2970 (2217 to 3930)	21.6 (16.4 to 28.1)	103998 (75526 to 140725)	681.6 (505 to 909.9)	5035 (3203 to 7606)	33.3 (21.6 to 49)	109032 (79889 to 147823)	714.9 (529.9 to 957.2)
	Male	13 (9 to 19)	0.3 (0.2 to 0.5)	89 (63 to 122)	2.2 (1.6 to 3)	10 (7 to 15)	0.3 (0.2 to 0.4)	270 (179 to 388)	6.5 (4.2 to 9.2)	9 (5 to 14)	0.2 (0.1 to 0.3)	279 (185 to 400)	6.7 (4.4 to 9.5)	83 (54 to 119)	0.7 (0.4 to 0.9)	612 (402 to 864)	4.8 (3.3 to 6.6)	45 (31 to 62)	0.4 (0.3 to 0.5)	1321 (855 to 1900)	9.9 (6.7 to 13.6)	58 (34 to 92)	0.5 (0.3 to 0.7)	1379 (892 to 1980)	10.4 (7 to 14.2)

		1990												2019											
		Incidence		Prevalence		Deaths		YLLs (Years of Life Lost)		YLDs (Years Lived with Disability)		DALYs (Disability-Adjusted Life Years)		Incidence		Prevalence		Deaths		YLLs (Years of Life Lost)		YLDs (Years Lived with Disability)		DALYs (Disability-Adjusted Life Years)	
Location	Sex	Number	Rate	Number	Rate	Number	Rate	Number	Rate	Number	Rate	Number	Rate	Number	Rate	Number	Rate	Number	Rate	Number	Rate	Number	Rate	Number	Rate
**Jordan**	Female	301 (234 to 377)	35.9 (27.6 to 45.2)	2519 (2065 to 3059)	305.2 (250.3 to 369.8)	160 (123 to 200)	21 (15.9 to 26.7)	5582 (4339 to 6887)	637.2 (492.4 to 792.2)	190 (126 to 279)	22.6 (14.9 to 33.4)	5772 (4486 to 7096)	659.8 (507.8 to 815.4)	2053 (1576 to 2636)	52.9 (41 to 67.4)	18387 (14602 to 23089)	474.7 (383.1 to 580)	674 (526 to 858)	19.9 (15.6 to 24.9)	21809 (16849 to 28024)	548.6 (427.6 to 698.7)	1368 (864 to 1982)	35 (22.3 to 50)	23176 (17862 to 29882)	583.6 (452.8 to 744.7)
	Male	4 (3 to 6)	0.7 (0.5 to 1)	30 (22 to 40)	4.3 (3.2 to 5.8)	3 (2 to 5)	0.6 (0.4 to 0.8)	82 (59 to 116)	11.4 (8.1 to 16.1)	3 (2 to 4)	0.4 (0.3 to 0.7)	85 (61 to 120)	11.8 (8.4 to 16.7)	22 (15 to 31)	0.6 (0.4 to 0.9)	180 (128 to 247)	4.9 (3.6 to 6.8)	11 (7 to 15)	0.4 (0.2 to 0.5)	285 (195 to 411)	7.5 (5.1 to 10.5)	16 (10 to 26)	0.5 (0.3 to 0.7)	301 (206 to 433)	7.9 (5.5 to 11.1)
**Kuwait**	Female	145 (131 to 162)	41.3 (37.6 to 45.8)	1289 (1160 to 1439)	376.7 (340.9 to 415)	51 (47 to 55)	17.7 (16.1 to 19.4)	1785 (1626 to 1971)	499 (456.1 to 545.3)	98 (66 to 137)	27.5 (18.9 to 38.1)	1883 (1719 to 2086)	526.5 (481.8 to 578.8)	716 (563 to 923)	42.8 (34.4 to 54.7)	6751 (5464 to 8513)	423.8 (355.1 to 521.9)	168 (135 to 215)	13 (10.6 to 16.6)	5573 (4443 to 7306)	329.4 (264.7 to 426.1)	492 (324 to 730)	29.5 (19.9 to 42.9)	6066 (4831 to 7893)	358.8 (290.3 to 461.8)
	Male	2 (1 to 2)	0.5 (0.4 to 0.7)	15 (12 to 19)	3.8 (3 to 4.9)	1 (1 to 1)	0.3 (0.3 to 0.4)	27 (22 to 34)	6.4 (5.2 to 8.1)	1 (1 to 2)	0.4 (0.2 to 0.5)	28 (23 to 35)	6.8 (5.4 to 8.6)	8 (5 to 11)	0.5 (0.4 to 0.8)	66 (48 to 92)	4.2 (3 to 5.9)	3 (2 to 5)	0.2 (0.2 to 0.4)	77 (55 to 110)	4.6 (3.3 to 6.7)	6 (4 to 9)	0.4 (0.2 to 0.6)	82 (58 to 119)	5 (3.6 to 7.2)
**Lebanon**	Female	613 (480 to 767)	48.4 (38.3 to 60.4)	5225 (4267 to 6354)	414.1 (342.8 to 500.1)	313 (250 to 392)	26 (21 to 32.3)	10122 (7928 to 12750)	783.4 (617.9 to 983.4)	391 (259 to 554)	30.8 (20.5 to 43.4)	10513 (8225 to 13210)	814.3 (642.1 to 1023.2)	3519 (2655 to 4617)	122.5 (92.1 to 160.7)	31097 (24277 to 39754)	1083.5 (844.5 to 1383.5)	1012 (777 to 1320)	35.5 (27.2 to 46.4)	28245 (21306 to 36799)	985.9 (742.5 to 1290.1)	2330 (1513 to 3414)	81 (52.4 to 118.5)	30575 (23260 to 40346)	1067 (808.6 to 1407.3)
	Male	10 (7 to 14)	1 (0.7 to 1.4)	67 (47 to 92)	6 (4.4 to 8.2)	7 (5 to 11)	0.8 (0.5 to 1.2)	171 (115 to 246)	15.4 (10.4 to 21.6)	7 (4 to 10)	0.6 (0.4 to 0.9)	178 (120 to 254)	16.1 (10.9 to 22.3)	34 (23 to 51)	1.5 (1 to 2.2)	249 (174 to 353)	10.5 (7.4 to 14.9)	16 (11 to 24)	0.7 (0.5 to 1.1)	320 (217 to 459)	13.6 (9.3 to 19.5)	24 (14 to 38)	1 (0.6 to 1.6)	343 (233 to 495)	14.6 (10 to 21)
**Libya**	Female	209 (164 to 278)	21 (16.4 to 27.8)	1886 (1551 to 2367)	193.6 (159.5 to 240.8)	111 (87 to 148)	11.7 (9.1 to 15.6)	3692 (2893 to 4873)	363.5 (283.8 to 481.5)	137 (88 to 201)	13.8 (8.9 to 20.3)	3830 (3004 to 5070)	377.4 (295.1 to 500.4)	1347 (944 to 1886)	41.4 (29.3 to 56.8)	11506 (8401 to 15649)	357.4 (268.1 to 472.2)	507 (364 to 693)	17.2 (12.5 to 23.2)	17315 (12243 to 24031)	523.6 (375.2 to 716.4)	875 (538 to 1355)	26.9 (16.6 to 40.6)	18190 (12936 to 25290)	550.5 (393.6 to 753.1)
	Male	7 (4 to 10)	0.7 (0.4 to 1)	45 (31 to 63)	4.3 (3 to 6)	5 (3 to 7)	0.5 (0.3 to 0.8)	122 (79 to 178)	11.3 (7.2 to 16.4)	4 (2 to 7)	0.4 (0.2 to 0.7)	127 (82 to 184)	11.7 (7.5 to 17)	19 (11 to 30)	0.7 (0.4 to 1.1)	140 (88 to 213)	5 (3.2 to 7.5)	11 (6 to 16)	0.4 (0.3 to 0.6)	284 (165 to 446)	9.7 (5.6 to 15)	13 (7 to 22)	0.5 (0.3 to 0.8)	298 (173 to 466)	10.1 (5.9 to 15.8)
**Morocco**	Female	2348 (1891 to 2838)	28.2 (22.6 to 33.9)	18627 (15550 to 22171)	229.6 (192.8 to 271.1)	1472 (1187 to 1766)	18.4 (14.9 to 22)	53860 (43121 to 65579)	631 (506.9 to 762.1)	1433 (933 to 2047)	17.3 (11.3 to 24.5)	55293 (44320 to 67421)	648.3 (519.8 to 783.8)	9755 (7043 to 13518)	52.5 (38.2 to 72)	82405 (62167 to 109991)	447.6 (341.1 to 590.6)	4372 (3195 to 5942)	24.4 (18.1 to 32.8)	152269 (109531 to 212953)	808.9 (585.5 to 1117.5)	6234 (3944 to 9348)	33.6 (21.6 to 49.8)	158502 (114796 to 219730)	842.5 (612.4 to 1157.9)
	Male	44 (29 to 63)	0.7 (0.4 to 1)	272 (195 to 363)	4 (2.9 to 5.3)	36 (24 to 51)	0.6 (0.4 to 0.8)	902 (598 to 1270)	12.8 (8.4 to 17.9)	27 (17 to 41)	0.4 (0.2 to 0.6)	930 (615 to 1309)	13.2 (8.6 to 18.5)	137 (84 to 205)	0.9 (0.6 to 1.4)	919 (604 to 1324)	5.9 (4 to 8.4)	92 (58 to 139)	0.7 (0.4 to 1)	2182 (1370 to 3231)	13.8 (8.5 to 20.5)	90 (51 to 142)	0.6 (0.3 to 0.9)	2272 (1425 to 3354)	14.4 (9 to 21.4)
**Oman**	Female	68 (46 to 99)	19.3 (13.2 to 28.4)	655 (489 to 882)	193.9 (147.5 to 257)	35 (24 to 52)	10.9 (7.5 to 16.2)	1134 (765 to 1655)	312.9 (211.5 to 462.9)	46 (28 to 73)	13.2 (8.1 to 20.7)	1180 (795 to 1718)	326 (221.6 to 479.8)	427 (346 to 511)	44.7 (36.8 to 52.9)	3894 (3232 to 4600)	413.8 (351.7 to 479)	124 (102 to 147)	15.9 (13.3 to 18.9)	3946 (3223 to 4725)	404.9 (335.3 to 480.9)	289 (193 to 412)	29.9 (20.2 to 41.7)	4235 (3446 to 5087)	434.8 (359.1 to 519)
	Male	7 (5 to 11)	2.3 (1.5 to 3.4)	52 (35 to 73)	13.6 (9.4 to 18.8)	5 (3 to 7)	1.8 (1.2 to 2.7)	139 (88 to 207)	35.5 (22.8 to 50.8)	5 (3 to 8)	1.4 (0.7 to 2.1)	144 (92 to 212)	36.9 (23.7 to 52.8)	21 (14 to 31)	2.5 (1.7 to 3.7)	181 (126 to 254)	18 (12.9 to 25.1)	9 (6 to 13)	1.4 (1 to 2.1)	253 (166 to 369)	24.6 (16.7 to 34.4)	16 (9 to 25)	1.7 (1 to 2.6)	269 (178 to 393)	26.3 (17.7 to 37)
**Palestine**	Female	176 (124 to 252)	33.5 (23.4 to 47.8)	1483 (1117 to 2010)	282.7 (214.4 to 381.2)	96 (68 to 137)	19.2 (13.6 to 27.6)	3092 (2184 to 4404)	574.5 (400.6 to 816.8)	112 (68 to 177)	21.1 (13 to 33.2)	3203 (2251 to 4558)	595.6 (417.5 to 845.3)	840 (688 to 1012)	57.1 (46.9 to 68.8)	7020 (5848 to 8365)	472.5 (396.9 to 558.4)	336 (278 to 402)	25.5 (21.2 to 30.5)	10601 (8731 to 12616)	702.9 (581.1 to 839.2)	537 (363 to 757)	36 (24.7 to 50.3)	11138 (9150 to 13244)	738.9 (609.5 to 879)

		1990												2019											
		Incidence		Prevalence		Deaths		YLLs (Years of Life Lost)		YLDs (Years Lived with Disability)		DALYs (Disability-Adjusted Life Years)		Incidence		Prevalence		Deaths		YLLs (Years of Life Lost)		YLDs (Years Lived with Disability)		DALYs (Disability-Adjusted Life Years)	
Location	Sex	Number	Rate	Number	Rate	Number	Rate	Number	Rate	Number	Rate	Number	Rate	Number	Rate	Number	Rate	Number	Rate	Number	Rate	Number	Rate	Number	Rate
	Male	1 (1 to 2)	0.3 (0.2 to 0.4)	10 (7 to 13)	2.3 (1.7 to 3.2)	1 (1 to 1)	0.2 (0.2 to 0.4)	23 (15 to 33)	5.2 (3.4 to 7.8)	1 (1 to 1)	0.2 (0.1 to 0.3)	24 (16 to 34)	5.4 (3.6 to 8.1)	5 (4 to 7)	0.5 (0.4 to 0.6)	43 (33 to 57)	3.5 (2.7 to 4.5)	3 (2 to 4)	0.3 (0.2 to 0.4)	83 (61 to 109)	6.4 (4.8 to 8.4)	4 (2 to 6)	0.3 (0.2 to 0.5)	87 (64 to 114)	6.7 (5.1 to 8.9)
**Qatar**	Female	30 (23 to 38)	48.9 (37 to 64.6)	255 (202 to 317)	420.4 (341.4 to 522)	14 (11 to 17)	28.2 (20.9 to 38)	503 (388 to 645)	766.6 (582.2 to 1005.6)	19 (13 to 28)	30.8 (20.2 to 44.2)	522 (405 to 667)	797.4 (605.2 to 1045.6)	382 (283 to 501)	103.7 (80.2 to 131.2)	3410 (2589 to 4419)	880.3 (701.3 to 1098.3)	86 (64 to 111)	36.9 (28.9 to 45.8)	3070 (2292 to 4009)	790.6 (610.1 to 996.8)	255 (160 to 379)	65.8 (43.5 to 92.9)	3325 (2493 to 4364)	856.4 (662.5 to 1074.6)
	Male	1 (0 to 1)	0.8 (0.5 to 1.2)	6 (4 to 8)	5.5 (3.7 to 7.9)	0 (0 to 1)	0.5 (0.3 to 0.8)	14 (8 to 21)	12.5 (7.6 to 18.5)	0 (0 to 1)	0.5 (0.3 to 0.8)	14 (8 to 21)	13 (8 to 19.3)	6 (4 to 10)	0.9 (0.5 to 1.3)	59 (39 to 85)	7.2 (4.9 to 10.2)	2 (1 to 3)	0.4 (0.3 to 0.6)	69 (42 to 105)	7.7 (4.8 to 11.2)	5 (3 to 8)	0.6 (0.3 to 1.1)	74 (44 to 112)	8.4 (5.2 to 12)
**Saudi Arabia**	Female	464 (331 to 626)	14.9 (10.8 to 20.1)	4529 (3522 to 5739)	154.1 (123 to 191)	307 (219 to 422)	10.7 (7.7 to 14.8)	10830 (7622 to 14931)	326.1 (230.7 to 447.4)	319 (201 to 481)	10.3 (6.6 to 15.4)	11149 (7894 to 15281)	336.4 (241.5 to 461.4)	5330 (3833 to 7262)	43.1 (31.9 to 57.1)	47120 (34765 to 62900)	394.2 (306.8 to 503.5)	1410 (1032 to 1868)	14.3 (10.9 to 18.7)	52462 (37864 to 70600)	417.4 (308.5 to 549.5)	3571 (2240 to 5339)	28.8 (18.4 to 42.2)	56033 (40441 to 75439)	446.1 (332.2 to 589.5)
	Male	11 (7 to 16)	0.3 (0.2 to 0.5)	89 (66 to 119)	2.4 (1.8 to 3.2)	9 (6 to 12)	0.3 (0.2 to 0.4)	243 (161 to 364)	6.1 (4 to 8.8)	8 (5 to 12)	0.2 (0.1 to 0.3)	251 (166 to 375)	6.3 (4.2 to 9.1)	41 (27 to 59)	0.4 (0.3 to 0.5)	364 (259 to 516)	3.2 (2.3 to 4.3)	17 (12 to 25)	0.2 (0.1 to 0.3)	515 (346 to 748)	4.1 (2.8 to 5.8)	32 (19 to 51)	0.3 (0.2 to 0.5)	547 (370 to 795)	4.4 (3 to 6.2)
**Sudan**	Female	730 (522 to 1021)	13.7 (9.7 to 19.5)	6071 (4715 to 7936)	118.7 (93.8 to 153.7)	501 (358 to 706)	9.9 (7 to 14.4)	17734 (12723 to 24697)	318.5 (228.7 to 446.8)	457 (293 to 732)	8.6 (5.6 to 13.8)	18190 (13030 to 25216)	327.1 (234.7 to 460)	2846 (1827 to 4005)	24 (16.2 to 33)	23052 (15851 to 31616)	196.4 (143.4 to 259.2)	1375 (931 to 1914)	13.1 (9.4 to 17.7)	48936 (31076 to 70606)	401.4 (270.6 to 559.8)	1802 (980 to 2825)	15 (8.8 to 22.9)	50738 (32468 to 73398)	416.4 (282.4 to 577.1)
	Male	27 (14 to 44)	0.6 (0.3 to 0.9)	157 (96 to 244)	3.2 (2 to 4.8)	23 (12 to 38)	0.5 (0.3 to 0.9)	611 (321 to 1024)	11.9 (6.3 to 19.6)	16 (8 to 28)	0.3 (0.2 to 0.6)	628 (331 to 1050)	12.2 (6.5 to 20.2)	56 (32 to 91)	0.5 (0.3 to 0.9)	384 (239 to 587)	3.6 (2.3 to 5.4)	39 (23 to 61)	0.4 (0.2 to 0.6)	1058 (612 to 1685)	9.4 (5.5 to 14.8)	38 (20 to 63)	0.4 (0.2 to 0.6)	1096 (638 to 1741)	9.8 (5.7 to 15.4)
**Syrian Arab Republic**	Female	422 (304 to 567)	13.7 (9.8 to 18.1)	4217 (3310 to 5288)	144.5 (114.7 to 179.7)	236 (169 to 312)	8.3 (6 to 11)	8245 (5916 to 11022)	256.7 (184.2 to 340.4)	293 (182 to 427)	9.6 (6 to 14.1)	8539 (6149 to 11423)	266.3 (191.5 to 353.6)	1873 (1303 to 2628)	26.9 (18.9 to 37.3)	17051 (12521 to 22955)	244.8 (184.6 to 324.5)	711 (500 to 1004)	11.3 (8.1 to 15.5)	22758 (15848 to 32520)	316.2 (222.8 to 446.3)	1248 (749 to 1916)	17.8 (10.9 to 27.1)	24006 (16758 to 34377)	334 (237.3 to 472.1)
	Male	3 (2 to 3)	0.1 (0.1 to 0.1)	23 (18 to 29)	0.8 (0.6 to 1)	2 (1 to 2)	0.1 (0 to 0.1)	55 (41 to 70)	1.6 (1.2 to 2)	2 (1 to 3)	0.1 (0 to 0.1)	57 (43 to 72)	1.6 (1.2 to 2.1)	9 (6 to 12)	0.1 (0.1 to 0.2)	74 (55 to 97)	1.1 (0.9 to 1.5)	4 (3 to 6)	0.1 (0.1 to 0.1)	124 (86 to 170)	1.8 (1.3 to 2.4)	7 (4 to 10)	0.1 (0.1 to 0.1)	130 (91 to 178)	1.9 (1.3 to 2.6)
**Tunisia**	Female	680 (557 to 840)	25 (20.4 to 30.9)	6221 (5208 to 7373)	230.5 (194.1 to 271.9)	354 (291 to 436)	14 (11.5 to 17.3)	11368 (9354 to 13948)	405.7 (333.5 to 497.9)	451 (302 to 653)	16.5 (11.2 to 23.9)	11818 (9707 to 14518)	422.1 (347.5 to 518.3)	3129 (2241 to 4237)	46 (33 to 62.2)	28854 (21971 to 37331)	424 (323.8 to 546)	1039 (753 to 1386)	15.7 (11.5 to 20.9)	31032 (22110 to 42022)	452.5 (323.5 to 610.4)	2112 (1314 to 3227)	31 (19.3 to 47.4)	33143 (23657 to 44761)	483.5 (345.2 to 650.3)
	Male	15 (10 to 20)	0.6 (0.4 to 0.8)	106 (77 to 143)	4.1 (3 to 5.4)	10 (7 to 14)	0.5 (0.3 to 0.6)	253 (171 to 354)	9.6 (6.6 to 13.3)	10 (6 to 16)	0.4 (0.2 to 0.6)	263 (178 to 368)	10 (6.8 to 13.8)	43 (26 to 69)	0.7 (0.4 to 1.1)	343 (226 to 520)	5.6 (3.7 to 8.3)	22 (13 to 34)	0.4 (0.2 to 0.6)	512 (301 to 812)	8.2 (4.8 to 12.8)	32 (17 to 55)	0.5 (0.3 to 0.9)	544 (317 to 860)	8.7 (5.2 to 13.5)
**Turkey**	Female	4047 (3201 to 5151)	19.4 (15.4 to 24.6)	35606 (29744 to 43550)	173.4 (145.5 to 210.6)	2450 (1949 to 3126)	12.3 (9.8 to 15.6)	79721 (63118 to 102190)	372.9 (295.4 to 475.9)	2598 (1722 to 3757)	12.4 (8.3 to 18)	82319 (65124 to 105081)	385.3 (306 to 488.2)	17130 (13440 to 21566)	36.1 (28.3 to 45.5)	155738 (127069 to 190476)	327.7 (267.8 to 400.7)	5926 (4729 to 7337)	12.6 (10.1 to 15.7)	164911 (129220 to 207565)	345.9 (271.3 to 435.3)	11381 (7681 to 16496)	24 (16.1 to 34.7)	176292 (138548 to 222264)	369.8 (291 to 464.6)
	Male	86 (56 to 131)	0.5 (0.3 to 0.8)	568 (390 to 815)	3.2 (2.2 to 4.6)	66 (43 to 100)	0.4 (0.3 to 0.6)	1763 (1103 to 2692)	9.4 (6.1 to 14.5)	56 (32 to 88)	0.3 (0.2 to 0.5)	1819 (1149 to 2772)	9.8 (6.4 to 14.9)	250 (177 to 351)	0.6 (0.4 to 0.8)	1950 (1424 to 2688)	4.6 (3.4 to 6.3)	123 (87 to 165)	0.3 (0.2 to 0.4)	2933 (2100 to 3987)	6.8 (4.9 to 9.2)	185 (115 to 288)	0.4 (0.3 to 0.7)	3118 (2237 to 4234)	7.3 (5.2 to 9.8)
**United Arab Emirates**	Female	86 (63 to 119)	40.8 (28.7 to 57.8)	742 (577 to 967)	359 (280.7 to 461.4)	45 (32 to 62)	25.5 (17.9 to 36.3)	1644 (1197 to 2243)	718 (506.1 to 1008.3)	56 (36 to 86)	25.8 (16.3 to 39.5)	1699 (1239 to 2336)	743.8 (526.3 to	1220 (872 to 1648)	57.5 (43.3 to 73.7)	10461 (7740 to 13923)	519.3 (412.8 to 644.5)	430 (313 to 571)	26.2 (20 to 33.6)	16825 (12024 to 22574)	753.5 (565.4 to 977.7)	796 (495 to 1212)	37.4 (24.2 to 54.5)	17621 (12543 to 23549)	791 (594 to

		1990												2019											
		Incidence		Prevalence		Deaths		YLLs (Years of Life Lost)		YLDs (Years Lived with Disability)		DALYs (Disability-Adjusted Life Years)		Incidence		Prevalence		Deaths		YLLs (Years of Life Lost)		YLDs (Years Lived with Disability)		DALYs (Disability-Adjusted Life Years)	
**Location**	**Sex**	**Number**	**Rate**	**Number**	**Rate**	**Number**	**Rate**	**Number**	**Rate**	**Number**	**Rate**	**Number**	**Rate**	**Number**	**Rate**	**Number**	**Rate**	**Number**	**Rate**	**Number**	**Rate**	**Number**	**Rate**	**Number**	**Rate**
													1041.7)												1020. 9)
	Male	4 (2 to 7)	1.2 (0.5 to 2.1)	33 (18 to 52)	7.8 (4.3 to 12.1)	3 (1 to 5)	1 (0.4 to 1.7)	98 (45 to 171)	21.1 (9.5 to 36.4)	3 (1 to 5)	0.8 (0.3 to 1.3)	101 (47 to 176)	21.9 (9.8 to 37.7)	51 (22 to 103)	1.1 (0.5 to 2)	421 (208 to 797)	8.5 (4.9 to 14.1)	25 (11 to 50)	0.7 (0.3 to 1.2)	947 (397 to 1935)	16.3 (7.6 to 29.3)	38 (16 to 80)	0.8 (0.4 to 1.5)	985 (415 to 2013)	17.1 (8.1 to 30.7)
**Yemen**	Female	378 (229 to 620)	12.6 (7.6 to 21)	3168 (2271 to 4651)	110 (80.1 to 158.4)	260 (157 to 428)	9.1 (5.4 to 15.2)	9050 (5495 to 14723)	289.6 (176.5 to 476.3)	235 (133 to 401)	7.9 (4.5 to 13.6)	9285 (5633 to 15062)	297.5 (181.5 to 490.7)	2001 (1418 to 2805)	22.7 (16.6 to 31.3)	15809 (11583 to 21685)	180.2 (137.6 to 239.9)	1085 (790 to 1506)	13.4 (10 to 18.4)	38639 (27428 to 54742)	420.3 (306.3 to 587.2)	1230 (742 to 1918)	13.8 (8.6 to 21.1)	39870 (28352 to 56502)	434.1 (314.8 to 605.8)
	Male	13 (6 to 23)	0.6 (0.3 to 1)	79 (45 to 131)	3.2 (1.9 to 5)	11 (5 to 19)	0.5 (0.3 to 0.9)	316 (158 to 571)	11.8 (6 to 21)	8 (4 to 15)	0.3 (0.2 to 0.6)	324 (163 to 584)	12.2 (6.2 to 21.6)	31 (19 to 50)	0.5 (0.3 to 0.7)	212 (142 to 327)	3 (2.1 to 4.5)	22 (14 to 35)	0.4 (0.2 to 0.6)	632 (389 to 1023)	8.4 (5.2 to 13.5)	21 (12 to 35)	0.3 (0.2 to 0.5)	653 (400 to 1054)	8.7 (5.4 to 14)

Supplementary table 6. All-ages numbers of deaths, DALYs, YLLs, and YLDs attributed to risk factors of breast cancer by sex in 1990 and 2019 and the percent change in this period.

Risk factor	Measure	1990		2019		% Change (1990 to 2019)	
		Female	Male	Female	Male	Female	Male
All risk factors	Deaths	1667 (959 to 2494)	23 (14 to 33)	6507 (3576 to 10469)	48 (30 to 68)	290.3 (198.8 to 387.9)	108.8 (44.6 to 180.8)
	DALYs	46992 (24371 to 72765)	644 (408 to 934)	171616 (80804 to 297481)	1359 (885 to 1871)	265.2 (169.9 to 361.8)	110.9 (44.7 to 185.4)
	YLLs	45416 (23657 to 70175)	625 (396 to 907)	162811 (76461 to 281684)	1295 (837 to 1787)	258.5 (165 to 354.6)	107.2 (42.2 to 180.1)
	YLDs	1575 (778 to 2627)	19 (11 to 30)	8805 (3885 to 15672)	64 (36 to 98)	458.9 (318.2 to 597.9)	230.7 (131.6 to 351.2)
Alcohol use	Deaths	89 (68 to 117)	8 (5 to 11)	236 (173 to 308)	18 (13 to 24)	164.3 (98 to 243.7)	123.1 (47.1 to 211.5)
	DALYs	3374 (2554 to 4483)	255 (176 to 349)	8601 (6291 to 11282)	573 (417 to 769)	154.9 (90.4 to 232.5)	124.6 (45.3 to 218.8)
	YLLs	3270 (2469 to 4340)	248 (170 to 339)	8095 (5873 to 10614)	547 (398 to 736)	147.5 (84.5 to 223.5)	120.5 (42.3 to 212.3)
	YLDs	104 (65 to 155)	7 (4 to 11)	507 (320 to 745)	27 (16 to 40)	387.7 (267.9 to 533.5)	263.2 (140.6 to 407.8)
Diet high in red meat	Deaths	222 (49 to 313)	7 (1 to 11)	677 (152 to 964)	15 (3 to 22)	205 (146.7 to 260.2)	108.3 (43.7 to 177.4)
	DALYs	8076 (1796 to 11242)	193 (42 to 304)	24129 (5414 to 34461)	401 (88 to 610)	198.8 (144.3 to 251.9)	108.1 (42.8 to 182.3)
	YLLs	7826 (1745 to 10894)	187 (41 to 295)	22894 (5131 to 32683)	382 (84 to 583)	192.5 (138.9 to 245)	104.5 (40.2 to 177.7)
	YLDs	250 (57 to 409)	6 (1 to 10)	1235 (291 to 2027)	19 (4 to 32)	394.3 (307.6 to 482.6)	224.2 (123.5 to 341.8)
Low physical activity	Deaths	183 (75 to 326)	0 (0 to 0)	680 (272 to 1201)	0 (0 to 0)	272.6 (184.2 to 354.5)	0 (0 to 0)
	DALYs	5526 (2348 to 10191)	0 (0 to 0)	20324 (7940 to 36490)	0 (0 to 0)	267.8 (181.3 to 350.7)	0 (0 to 0)
	YLLs	5344 (2278 to 9859)	0 (0 to 0)	19263 (7497 to 34582)	0 (0 to 0)	260.4 (174.7 to 342.8)	0 (0 to 0)
	YLDs	182 (72 to 346)	0 (0 to 0)	1061 (415 to 2036)	0 (0 to 0)	484.3 (358.1 to 602.1)	0 (0 to 0)
Smoking	Deaths	179 (118 to 253)	0 (0 to 0)	499 (337 to 682)	0 (0 to 0)	179.2 (113.5 to 247.2)	0 (0 to 0)
	DALYs	5827 (3678 to 8384)	0 (0 to 0)	15634 (10009 to 21774)	0 (0 to 0)	168.3 (107.6 to 233.3)	0 (0 to 0)
	YLLs	5639 (3558 to 8153)	0 (0 to 0)	14736 (9472 to 20579)	0 (0 to 0)	161.3 (102.4 to 224.6)	0 (0 to 0)
	YLDs	188 (103 to 304)	0 (0 to 0)	898 (500 to 1382)	0 (0 to 0)	376.2 (269.9 to 490.4)	0 (0 to 0)
Secondhand smoke	Deaths	402 (95 to 704)	8 (2 to 16)	1133 (270 to 1992)	16 (4 to 29)	181.5 (126.6 to 233.6)	96.2 (40.5 to 163.9)
	DALYs	14676 (3457 to 25719)	210 (48 to 396)	41012 (9770 to 72205)	415 (95 to 742)	179.4 (127.4 to 229.5)	97.6 (40.5 to 163.4)
	YLLs	14227 (3334 to 24894)	203 (46 to 383)	38944 (9271 to 68900)	395 (90 to 708)	173.7 (122.9 to 222.4)	94.2 (38 to 159.2)
	YLDs	449 (99 to 853)	7 (2 to 13)	2068 (466 to 3867)	20 (4 to 38)	360.2 (278.9 to 439.2)	201.7 (113.5 to 310.2)
High body-mass index	Deaths	183 (-196 to 595)	0 (0 to 0)	987 (-603 to 2667)	0 (0 to 0)	438.8 (-614.7 to 1724.7)	0 (0 to 0)
	DALYs	-3030 (-16773 to 7892)	0 (0 to 0)	-7656 (-66973 to 36339)	0 (0 to 0)	152.7 (-1423.7 to 1875.4)	0 (0 to 0)
	YLLs	-3018 (-16294 to 7572)	0 (0 to 0)	-7107 (-62997 to 35046)	0 (0 to 0)	135.5 (-1440 to 1884.7)	0 (0 to 0)
	YLDs	-12 (-431 to 343)	0 (0 to 0)	-549 (-3608 to 1804)	0 (0 to 0)	4464.8 (-2658.8 to 4845.3)	0 (0 to 0)
High fasting plasma glucose	Deaths	546 (98 to 1274)	0 (0 to 0)	2976 (604 to 6690)	0 (0 to 0)	445.3 (333 to 569.6)	0 (0 to 0)
	DALYs	15797 (2804 to 37471)	0 (0 to 0)	84912 (17377 to 192838)	0 (0 to 0)	437.5 (330.3 to 554)	0 (0 to 0)
	YLLs	15266 (2705 to 36170)	0 (0 to 0)	80520 (16417 to 183065)	0 (0 to 0)	427.4 (320.5 to 542.9)	0 (0 to 0)
	YLDs	531 (88 to 1331)	0 (0 to 0)	4392 (819 to 10479)	0 (0 to 0)	727 (580.6 to 906)	0 (0 to 0)

Supplementary table 7. Age-standardized rates per 100000 of deaths, DALYs, YLLs, and YLDs attributed to risk factors of breast cancer in 1990 and 2019 and the percent change in this period.

Risk factor	Measure	1990		2019		% Change (1990 to 2019)	
		Female	Male	Female	Male	Female	Male
All risk factors	Deaths	2 (1.2 to 2.9)	0 (0 to 0)	3.2 (1.8 to 5)	0 (0 to 0)	61.1 (24.3 to 99)	-21 (-45.1 to 7.3)
	DALYs	51.7 (28.5 to 78.1)	0.7 (0.4 to 1)	78 (40.9 to 129.3)	0.6 (0.4 to 0.8)	50.9 (15.2 to 88.9)	-19.3 (-44.6 to 8.4)
	YLLs	49.9 (27.6 to 75.7)	0.7 (0.4 to 1)	73.8 (38.6 to 122.4)	0.5 (0.3 to 0.7)	48.1 (12.5 to 85.9)	-20.8 (-45.5 to 6.8)
	YLDs	1.8 (0.9 to 2.9)	0 (0 to 0)	4.1 (2 to 7.2)	0 (0 to 0)	130.1 (78.7 to 182.5)	25.8 (-12.3 to 70.5)
Alcohol use	Deaths	0.1 (0.1 to 0.1)	0 (0 to 0)	0.1 (0.1 to 0.1)	0 (0 to 0)	8.9 (-18 to 40.7)	-13.6 (-42.8 to 21.4)
	DALYs	3.2 (2.4 to 4.2)	0.2 (0.2 to 0.3)	3.3 (2.4 to 4.3)	0.2 (0.2 to 0.3)	2.4 (-23.6 to 32.7)	-13.1 (-43.3 to 22)
	YLLs	3.1 (2.3 to 4.1)	0.2 (0.2 to 0.3)	3.1 (2.2 to 4)	0.2 (0.1 to 0.3)	-0.5 (-25.6 to 29.4)	-14.7 (-44.3 to 19.4)
	YLDs	0.1 (0.1 to 0.1)	0 (0 to 0)	0.2 (0.1 to 0.3)	0 (0 to 0)	93.8 (46 to 150.4)	40.4 (-6.4 to 97.3)
Diet high in red meat	Deaths	0.2 (0.1 to 0.3)	0 (0 to 0)	0.3 (0.1 to 0.4)	0 (0 to 0)	22.8 (-1.5 to 45)	-20.6 (-45.2 to 6.5)
	DALYs	7.7 (1.7 to 10.8)	0.2 (0 to 0.3)	9.2 (2.1 to 13.1)	0.2 (0 to 0.3)	18.7 (-3.5 to 39.9)	-20.3 (-45 to 7.1)
	YLLs	7.5 (1.7 to 10.5)	0.2 (0 to 0.3)	8.7 (2 to 12.4)	0.2 (0 to 0.2)	16.2 (-5.5 to 37.3)	-21.8 (-46 to 5.1)
	YLDs	0.2 (0.1 to 0.4)	0 (0 to 0)	0.5 (0.1 to 0.8)	0 (0 to 0)	93.8 (59.8 to 127.9)	23.9 (-13.7 to 67.6)
Low physical activity	Deaths	0.2 (0.1 to 0.4)	0 (0 to 0)	0.3 (0.1 to 0.6)	0 (0 to 0)	48.3 (12.9 to 79.7)	0 (0 to 0)
	DALYs	5.8 (2.4 to 10.5)	0 (0 to 0)	8.5 (3.3 to 15.1)	0 (0 to 0)	45.7 (11.4 to 78.3)	0 (0 to 0)
	YLLs	5.6 (2.3 to 10.2)	0 (0 to 0)	8 (3.1 to 14.4)	0 (0 to 0)	42.8 (9.3 to 75.2)	0 (0 to 0)
	YLDs	0.2 (0.1 to 0.4)	0 (0 to 0)	0.5 (0.2 to 0.9)	0 (0 to 0)	125.4 (79.1 to 169.6)	0 (0 to 0)
Smoking	Deaths	0.2 (0.1 to 0.3)	0 (0 to 0)	0.2 (0.2 to 0.3)	0 (0 to 0)	15.2 (-12.7 to 43.3)	0 (0 to 0)
	DALYs	6 (3.8 to 8.6)	0 (0 to 0)	6.5 (4.2 to 8.9)	0 (0 to 0)	8.3 (-16.3 to 34.2)	0 (0 to 0)
	YLLs	5.8 (3.7 to 8.3)	0 (0 to 0)	6.1 (4 to 8.4)	0 (0 to 0)	5.5 (-18.4 to 31)	0 (0 to 0)
	YLDs	0.2 (0.1 to 0.3)	0 (0 to 0)	0.4 (0.2 to 0.6)	0 (0 to 0)	90.7 (49.7 to 136.3)	0 (0 to 0)
Secondhand smoke	Deaths	0.4 (0.1 to 0.7)	0 (0 to 0)	0.5 (0.1 to 0.8)	0 (0 to 0)	11.8 (-10.1 to 32)	-26.7 (-47.8 to -0.4)
	DALYs	14.1 (3.3 to 24.7)	0.2 (0.1 to 0.5)	15.5 (3.7 to 27.3)	0.2 (0 to 0.3)	10 (-11.1 to 30)	-24.7 (-46.1 to 0.7)
	YLLs	13.6 (3.2 to 23.9)	0.2 (0.1 to 0.4)	14.7 (3.5 to 25.9)	0.2 (0 to 0.3)	7.8 (-12.7 to 27.1)	-26.1 (-47.1 to -1.3)
	YLDs	0.4 (0.1 to 0.8)	0 (0 to 0)	0.8 (0.2 to 1.5)	0 (0 to 0)	78.5 (47.3 to 108.9)	14.3 (-18.4 to 56.2)
High body-mass index	Deaths	0.3 (-0.1 to 0.8)	0 (0 to 0)	0.7 (0 to 1.6)	0 (0 to 0)	109.5 (-86.3 to 464.1)	0 (0 to 0)
	DALYs	1.7 (-11.4 to 13.4)	0 (0 to 0)	6.3 (-15.2 to 27)	0 (0 to 0)	261.9 (-670.7 to 928.3)	0 (0 to 0)
	YLLs	1.6 (-11.1 to 12.9)	0 (0 to 0)	6 (-14.3 to 25.6)	0 (0 to 0)	276.9 (-680.9 to 926.1)	0 (0 to 0)
	YLDs	0.2 (-0.3 to 0.6)	0 (0 to 0)	0.3 (-0.8 to 1.4)	0 (0 to 0)	108.3 (-415 to 1028.6)	0 (0 to 0)
High fasting plasma glucose	Deaths	0.7 (0.1 to 1.5)	0 (0 to 0)	1.4 (0.3 to 3.1)	0 (0 to 0)	118.1 (72.5 to 169.9)	0 (0 to 0)
	DALYs	17.1 (3 to 40.2)	0 (0 to 0)	36.5 (7.4 to 82.1)	0 (0 to 0)	113.9 (70.3 to 161.8)	0 (0 to 0)
	YLLs	16.5 (2.9 to 38.9)	0 (0 to 0)	34.6 (7 to 78.4)	0 (0 to 0)	109.9 (67 to 157)	0 (0 to 0)
	YLDs	0.6 (0.1 to 1.5)	0 (0 to 0)	1.9 (0.4 to 4.6)	0 (0 to 0)	224.1 (166.6 to 294.5)	0 (0 to 0)

Supplementary table 8. Existence of national screening program for breast cancer, data provided by the Global Health Observatory (GHO), powered by the World Health Organization. (Source: https://www.who.int/data/gho) (Data of Palestine was not available in the GHO database)

Country	2015	2017	2019
Afghanistan	No	No	No
Algeria	Yes	Yes	Yes
Bahrain	Yes	Yes	Yes
Egypt	Yes	Yes	Yes
Iran (Islamic Republic of)	Yes	Yes	Yes
Iraq	Yes	Yes	Yes
Jordan	Yes	Yes	Yes
Kuwait	Yes	Yes	Yes
Lebanon	Yes	Yes	Yes
Libya	No	No	No
Morocco	Yes	Yes	Yes
Oman	Yes	Yes	Yes
Qatar	Yes	Yes	Yes
Saudi Arabia	Yes	Yes	Yes
Sudan	No	No	No
Syrian Arab Republic	Yes	Yes	Yes
Tunisia	No response	Yes	Yes
Turkey	Yes	Yes	Yes
United Arab Emirates	Yes	Yes	Yes
Yemen	Yes	No	No

Supplementary table 9. Existence of operational policy/strategy/action plan for cancer, data provided by the Global Health Observatory (GHO), powered by the World Health Organization. (Source: https://www.who.int/data/gho) (Data of Palestine was not available in the GHO database)

Country	2013	2015	2017	2019
Afghanistan	No	No	Yes	Yes
Algeria	Yes	Yes	Yes	Yes
Bahrain	Yes	Yes	Yes	Yes
Egypt	No	No	Yes	Yes
Iran (Islamic Republic of)	Yes	Yes	Yes	Yes
Iraq	Yes	Yes	Yes	Yes
Jordan	Yes	No	Yes	No
Kuwait	No	Yes	Yes	Yes
Lebanon	Don't know	Yes	Yes	Yes
Libya	No	No	No	No
Morocco	Yes	Yes	Yes	Yes
Oman	No	No	No	Yes
Qatar	Yes	Yes	Yes	Yes
Saudi Arabia	Yes	Yes	Yes	Yes
Sudan	Yes	No	No	Yes
Syrian Arab Republic	Yes	No	No	No
Tunisia	No	Yes	Yes	Yes
Turkey	Yes	Yes	Yes	Yes
United Arab Emirates	No	Yes	Yes	Yes
Yemen	No	Yes	No	No

Supplementary table 10. Existence of population-based cancer registry, data provided by the Global Health Observatory (GHO), powered by the World Health Organization. (Source: https://www.who.int/data/gho) (Data of Palestine was not available in the GHO database)

Country	2013	2015	2017	2019
Afghanistan	No	No	No	Yes
Algeria	Yes	Yes	No	Yes
Bahrain	No	Yes	Yes	Yes
Egypt	Yes	Yes	Yes	Yes
Iran (Islamic Republic of)	Yes	Yes	Yes	Yes
Iraq	No	No	Yes	Yes
Jordan	Yes	Yes	Yes	Yes
Kuwait	Yes	Yes	Yes	Yes
Lebanon	No	Yes	Yes	Yes
Libya	No	Yes	Yes	No
Morocco	Yes	Yes	Yes	Yes
Oman	Yes	Yes	Yes	Yes
Qatar	Yes	Yes	Yes	Yes
Saudi Arabia	Yes	Yes	Yes	Yes
Sudan	Yes	No	Yes	Yes
Syrian Arab Republic	No	No	No	No
Tunisia	Yes	Yes	Yes	Yes
Turkey	Yes	Yes	Yes	Yes
United Arab Emirates	Yes	Yes	Yes	Yes
Yemen	No	Yes	Yes	No

Supplementary table 11. The Universal Health Coverage (UHC) effective coverage index and the breast cancer treatment component estimations for the countries of the North Africa and Middle East region, provided by Global Burden of Disease Study 2019. *(Reference: GBD 2019 Universal Health Coverage Collaborators. Measuring universal health coverage based on an index of effective coverage of health services in 204 countries and territories, 1990-2019: a systematic analysis for the Global Burden of Disease Study 2019. Lancet. 2020 Oct 17;396(10258):1250-1284. doi: 10.1016/S0140-6736(20)30750-9. Epub 2020 Aug 27. PMID: 32861314; PMCID: PMC7562819.)*

	**UHC effective coverage index**		**Breast cancer treatment**	
**location**	**1990**	**2019**	**1990**	**2019**
Afghanistan	22 (17.1 to 27.2)	39.3 (33.6 to 45.1)	3.8 (0 to 8.3)	17.5 (13.6 to 21.5)
Algeria	48.6 (45.3 to 51.9)	64.9 (61.4 to 68.3)	41.7 (37.5 to 46.3)	69.6 (65.8 to 73)
Bahrain	54.8 (52.2 to 57.4)	70.6 (66.4 to 74.5)	42.3 (37.9 to 46.9)	78.9 (75.9 to 81.9)
Egypt	38.2 (35.1 to 40.9)	54.8 (49 to 60.6)	25.3 (22 to 28.5)	56.6 (52.5 to 60.7)
Iran (Islamic Republic of)	52.5 (50.1 to 54.8)	69.5 (67.4 to 71.4)	48.4 (38.8 to 58.2)	77.5 (72.1 to 82.2)
Iraq	45.4 (42.5 to 48.2)	57.7 (53.5 to 62.4)	35.5 (31.4 to 39.8)	64.3 (61 to 67.7)
Jordan	53 (49.8 to 56.4)	70 (66.9 to 72.8)	40.4 (25.8 to 53.5)	75 (71.8 to 78.3)
Kuwait	65.3 (61.7 to 67.9)	81.8 (78.6 to 84.9)	66.7 (63.2 to 70.2)	86.9 (83.8 to 89.9)
Lebanon	54.2 (51.4 to 56.7)	74.5 (72.5 to 76.6)	49.2 (45.3 to 53.4)	84.7 (81.4 to 87.7)
Libya	57.8 (54.2 to 61.6)	66.3 (62.3 to 70.6)	44.2 (40 to 48.3)	65.5 (61.8 to 68.9)
Morocco	41 (38 to 44.3)	58 (54.6 to 62.5)	24.2 (20 to 28.2)	54.3 (50.4 to 57.9)
Oman	53.6 (49.7 to 57.6)	71.2 (69 to 73.3)	44.1 (40 to 47.9)	79.3 (76.2 to 82.4)
Palestine	47.2 (43.9 to 50.6)	61.2 (58.3 to 64.1)	43.4 (39.3 to 47.7)	64.5 (61 to 68.1)
Qatar	59.9 (55.4 to 63.7)	80.4 (75.9 to 83.3)	46.1 (41.5 to 50.3)	84.8 (81.7 to 87.8)
Saudi Arabia	50.2 (45.3 to 55.6)	64.2 (59.6 to 68.9)	18.8 (0.2 to 46.9)	79.7 (76 to 83.2)
Sudan	27.5 (22.4 to 33.2)	51.8 (47.1 to 56.6)	15.6 (11.3 to 20)	45.6 (35.3 to 53)
Syrian Arab Republic	45.4 (42 to 49.3)	57.6 (52.6 to 62.5)	36.7 (32.9 to 40.3)	68.7 (65.2 to 72.1)
Tunisia	55.9 (53 to 58.9)	68.1 (61.7 to 73.8)	47.4 (43.6 to 51.2)	78.4 (75.4 to 81.6)
Turkey	47.8 (44.9 to 50.7)	69.2 (64.6 to 73.9)	32.7 (28.8 to 36.5)	78.8 (76 to 81.6)
United Arab Emirates	55.1 (50.5 to 59.7)	63.4 (56.8 to 69.9)	35 (30.7 to 38.9)	60.3 (56.3 to 64.4)
Yemen	30.2 (24.1 to 37.7)	49 (43.8 to 53.9)	15.9 (11.9 to 20.1)	37.9 (33.6 to 42.2)

Supplementary figure 3. Age-standardized rates of incidence, prevalence, deaths, and DALYs of breast cancer stratified by sex, in different age groups in the North Africa and Middle East region, in 1990 and 2019.

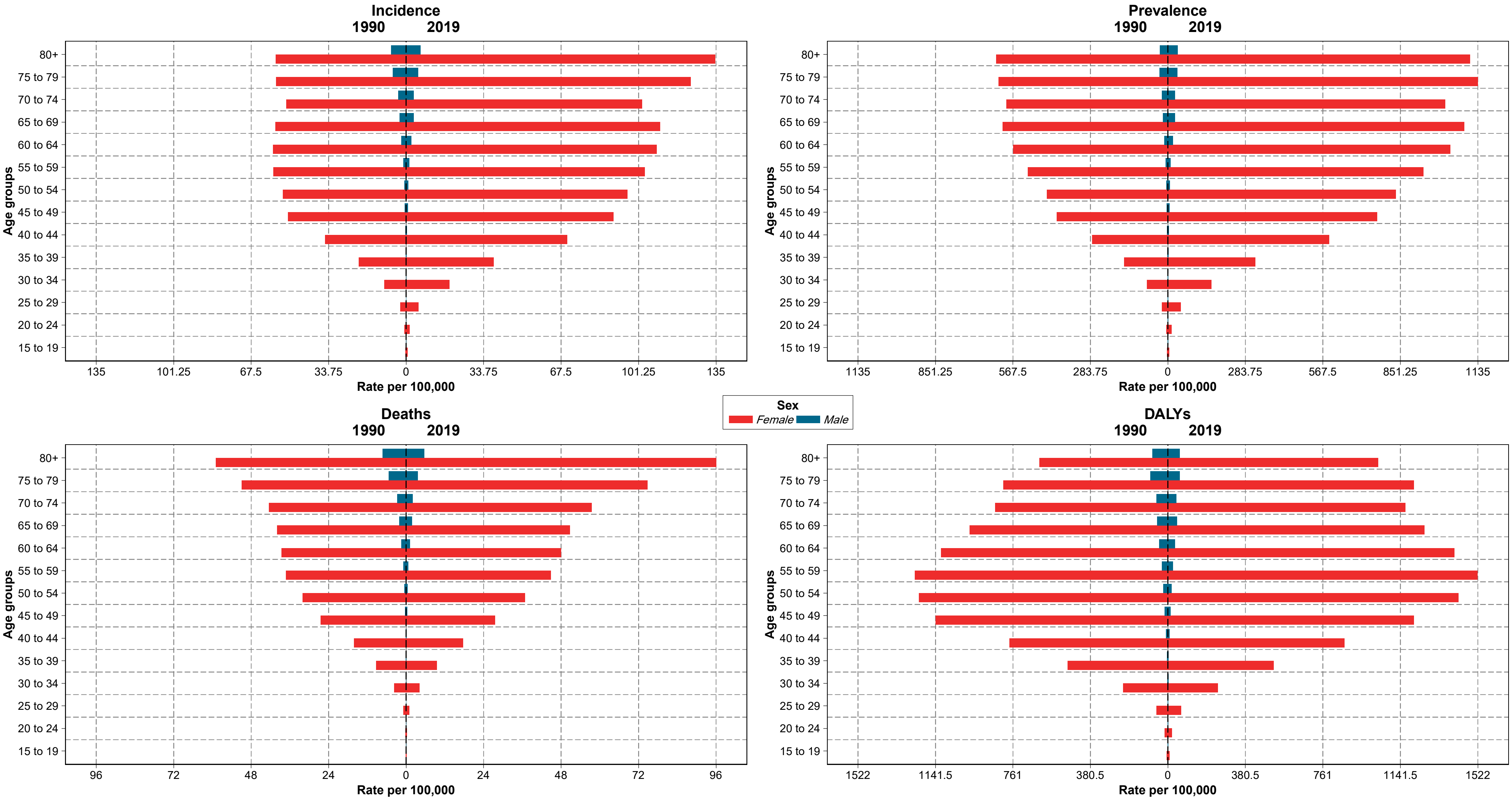

Supplementary figure 4. The all-ages number of incidences, prevalence, deaths, and DALYs of breast cancer by age groups during the 1990-2019 period in females of the North Africa and Middle East region.

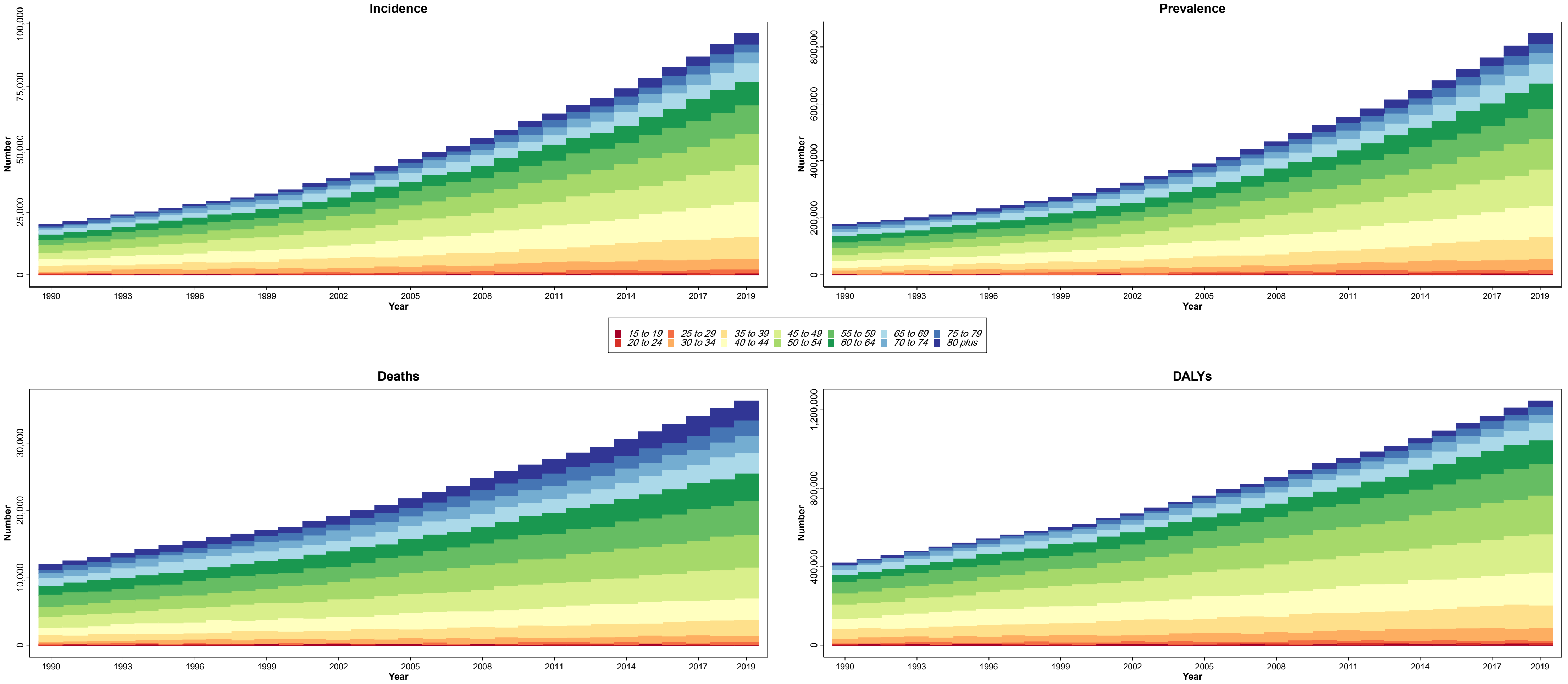

Supplementary figure 5. The all-ages number of incidences, prevalence, deaths, and DALYs of breast cancer by countries during the 1990-2019 period in females of the North Africa and Middle East region.

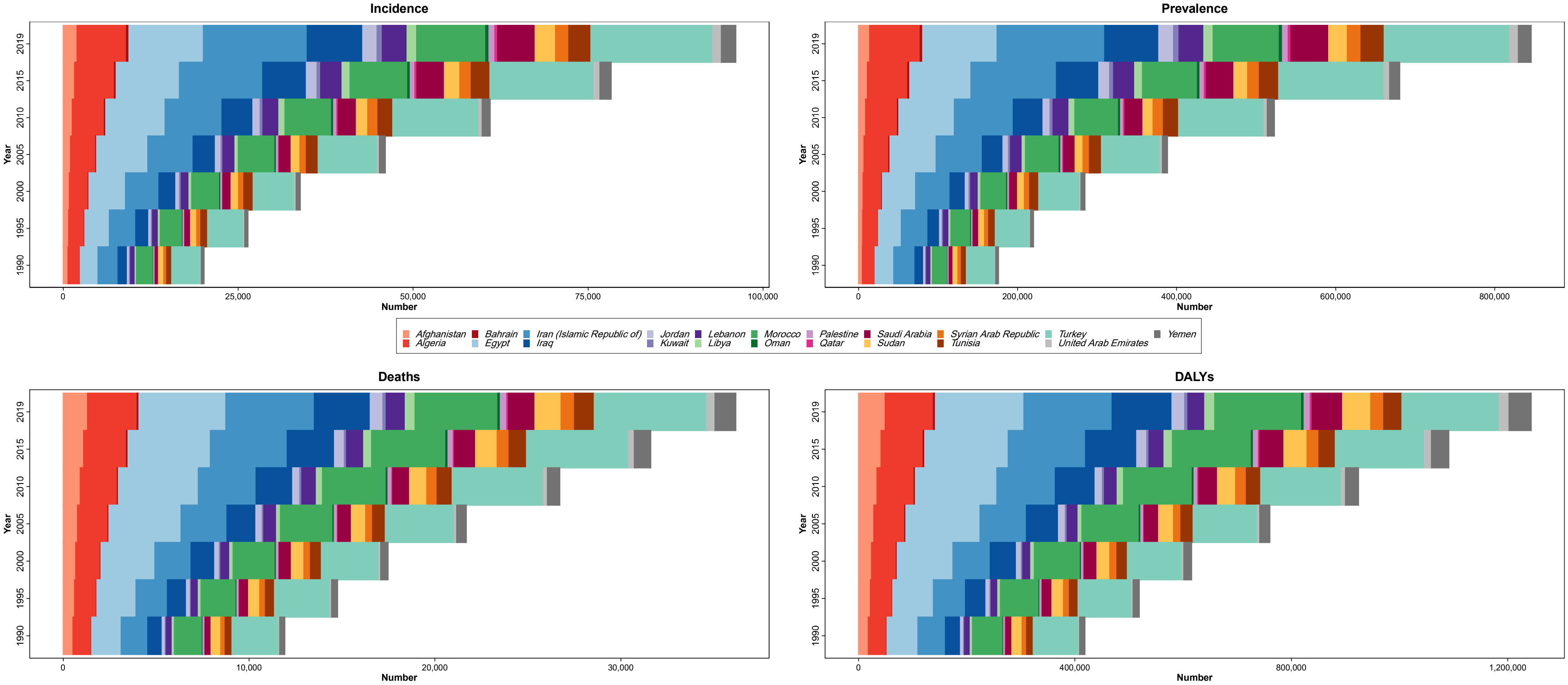

Supplementary figure 6. Time trend of age-standardized rates of incidence, prevalence, deaths, and DALYs of breast cancer for both sexes by countries, 1990-2019.

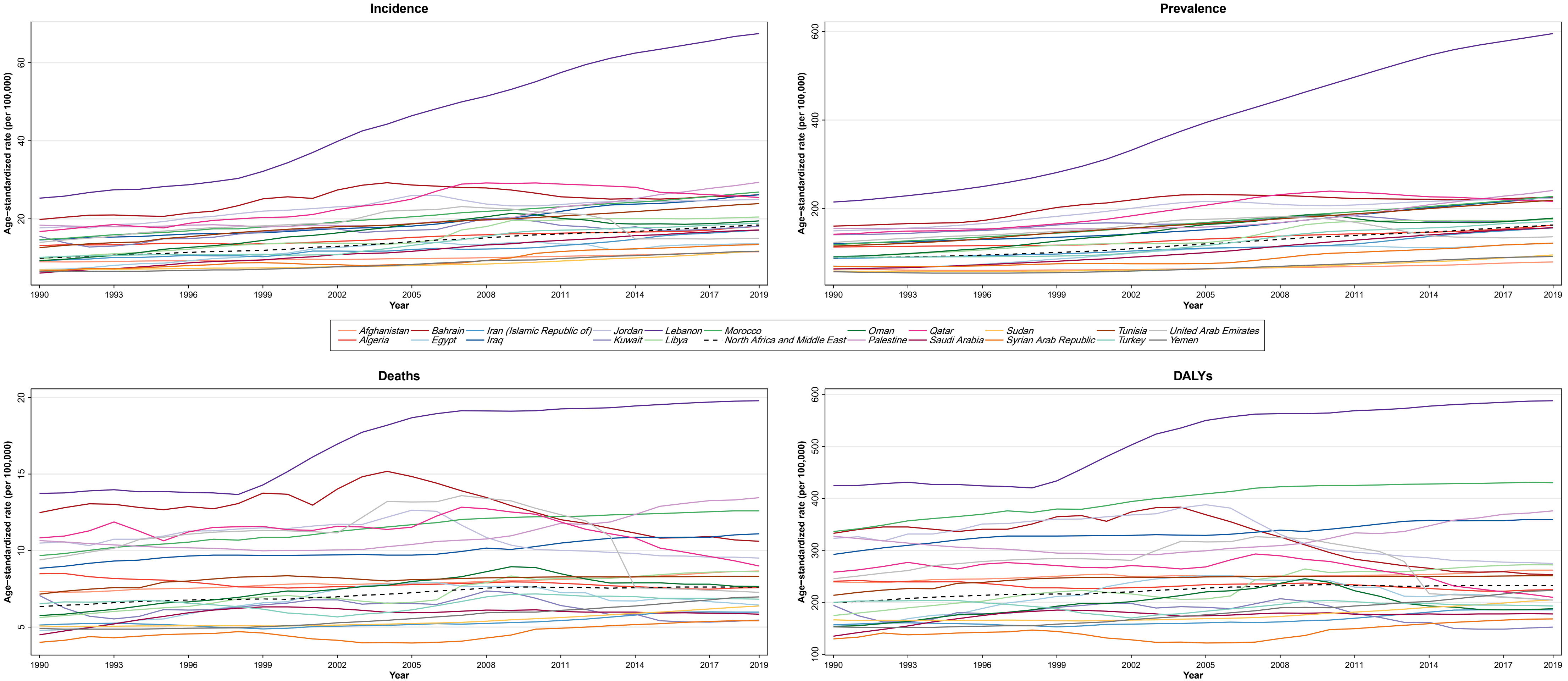

Supplementary figure 7. Scatter of age-standardized rates of incidence, prevalence, deaths, and DALYs of breast cancer by countries in the North Africa and Middle East region during the 1990-2010 against 2010-2019 period.

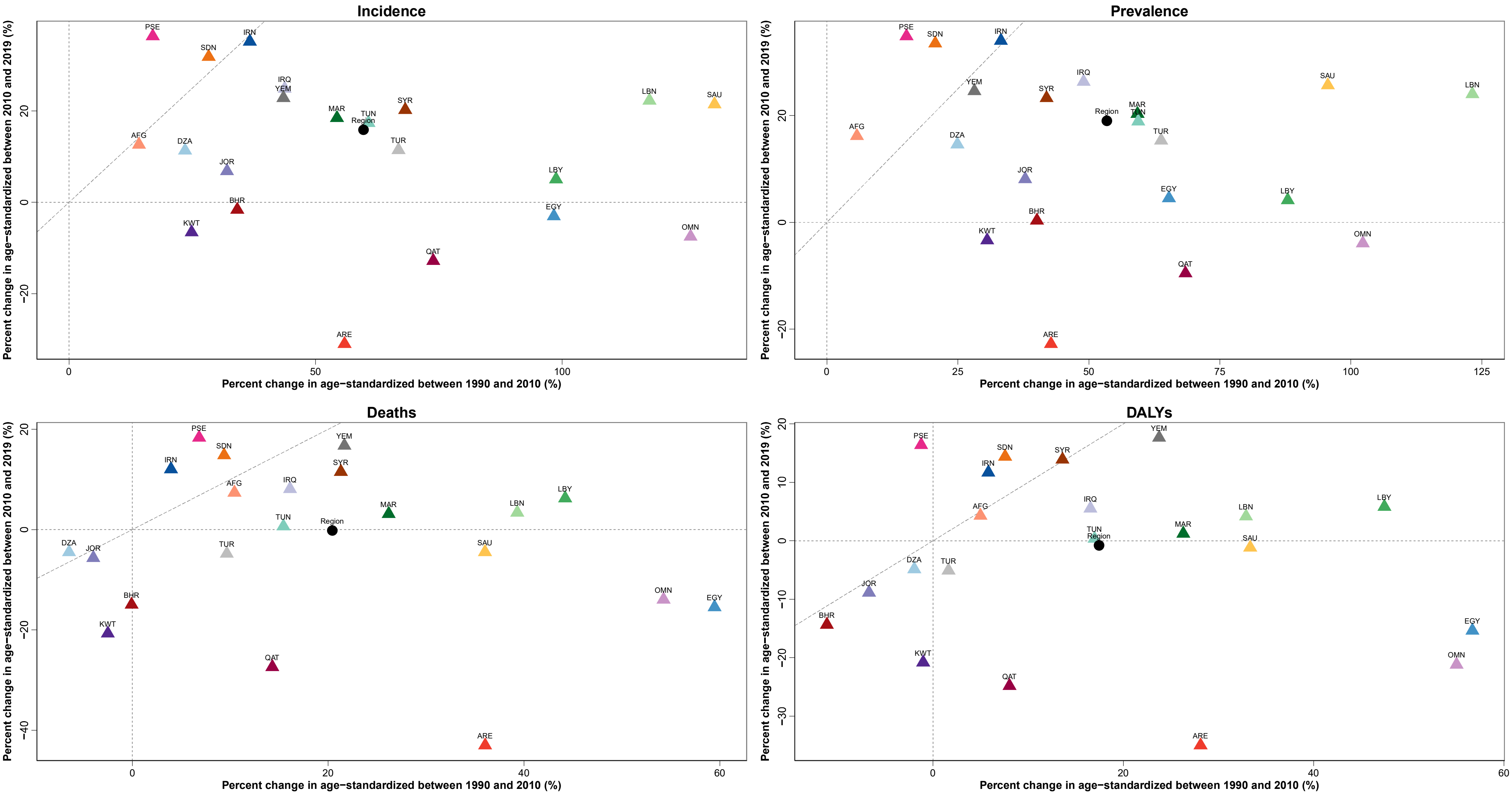

Supplementary figure 8. Ranking of age-standardized rates of female breast cancer prevalence and DALYs by countries in the North Africa and Middle East region, in 1990 and 2019.

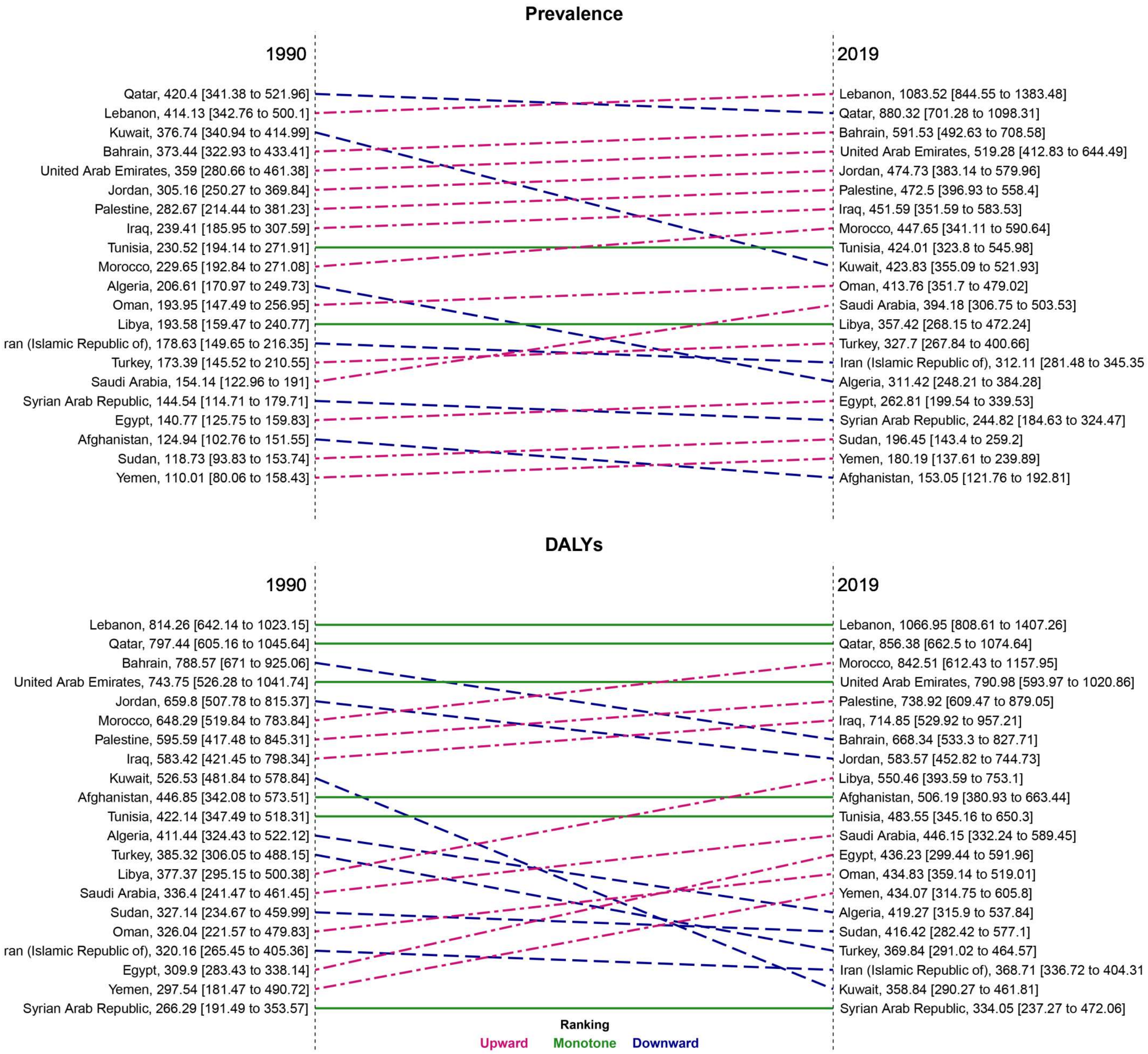

Supplementary figure 9. Age-standardized rates of deaths, DALYs, YLLs, and YLDs attributed to breast cancer risk factors in females in the North Africa and Middle East region in 1990.

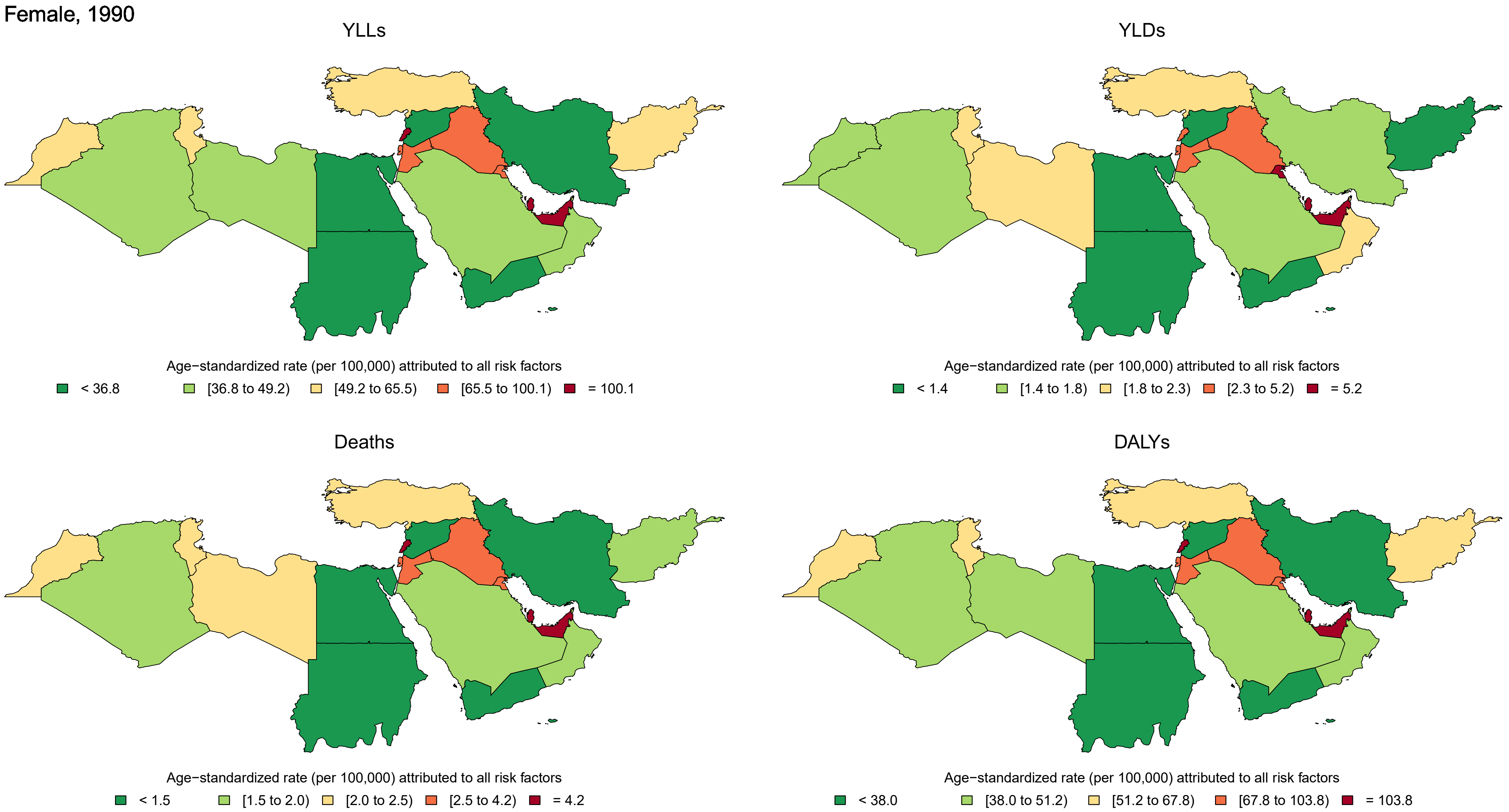

Supplementary figure 10. Age-standardized rates of deaths, DALYs, YLLs, and YLDs attributed to breast cancer risk factors in females in the North Africa and Middle East region in 2019.

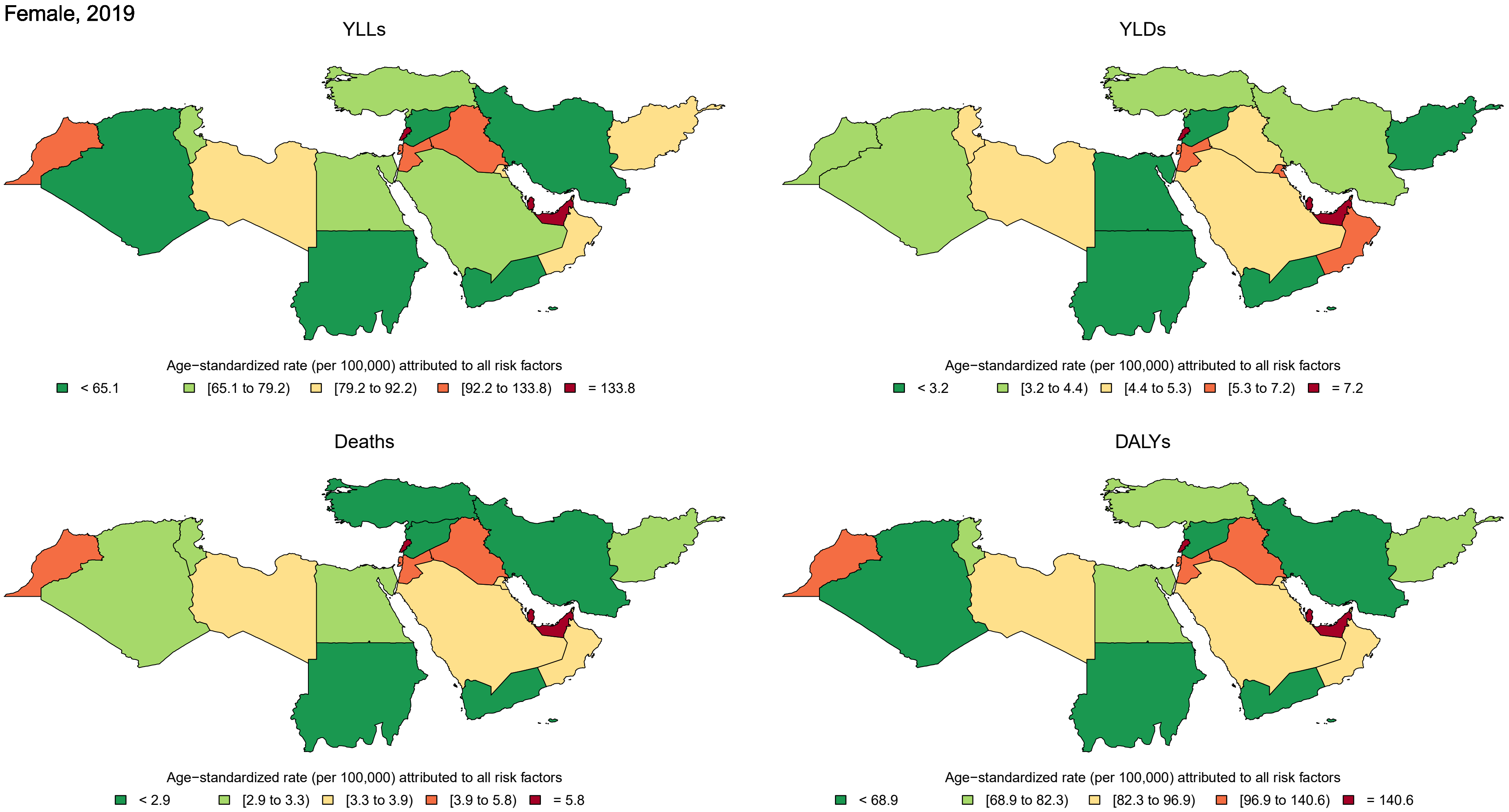

Authors' Contributions

Providing data or critical feedback on data sources

Hedayat Abbastabar, Hassan Abidi, Hassan Abolhassani, Hiwa Abubaker Ali, Juan Manuel Acuna, Sima Afrashteh, Sajjad Ahmad, Ali Ahmadi, Sepideh Ahmadi, Haroon Ahmed, Tarik Ahmed Rashid, Mostafa Akbarzadeh-Khiavi, Hanadi Al Hamad, Beriwan Abdulqadir Ali, Syed Mohamed Aljunid, Sami Almustanyir, Jalal Arabloo, Seyyed Shamsadin Athari, Sina Azadnajafabad, Sara Bagherieh, Saeed Bahadory, Mohammad Behnammoghadam, Akshaya Srikanth Bhagavathula, Somayeh Bohlouli, Wolfram Manuel Wolfram Brück, Aso Mohammad Darwesh, Mostafa Dianatinasab, Fariba Dorostkar, Ebrahim Eini, Iman El Sayed, Maysaa El Sayed Zaki, Sharareh Eskandarieh, Farshad Farzadfar, Mansour Ghafourifard, Ahmad Ghashghaee, Kimiya Gohari, Pouya Goleij, Mohamad Golitaleb, Nima Hafezi-Nejad, Rabih Halwani, Shafiul Haque, Soheil Hassanipour, Mehdi Hosseinzadeh, Amirreza Javadi Mamaghani, Tahereh Javaheri, Leila R Kalankesh, Yousef Saleh Khader, Sang-woong Lee, Mozhgan Letafat-nezhad, Somayeh Livani, Farzan Madadizadeh, Ahmad R Mafi, Ata Mahmoodpoor, Fariborz Mansour-Ghanaei, Mohammad Ali Mansournia, Entezar Mehrabi Nasab, Karzan Abdulmuhsin Mohammad, Mokhtar Mohammadi, Ali H Mokdad, Sara Momtazmanesh, Christopher J L Murray, Mohsen Naghavi, Zuhair S Natto, Maryam Noori, Zahra Zahid Piracha, Naeimeh Pourtaheri, Navid Rabiee, Fakher Rahim, Amir Masoud Rahmani, Amirfarzan Rashidi, Mohammad-Mahdi Rashidi, Reza Rawassizadeh, Nima Rezaei, Siamak Sabour, Basema Saddik, Umar Saeed, Sahar Saeedi Moghaddam, Abdallah M Samy, Brijesh Sathian, Fariba Shahraki-Sanavi, Masood Ali Shaikh, Reza Shirkoohi, Parnian Shobeiri, Mohammad Sadegh Soltani-Zangbar, Majid Taheri, Mohamad-Hani Temsah, Amir Tiyuri, Irfan Ullah, Sahel Valadan Tahbaz, Bay Vo, Yasir Waheed, Seyed Hossein Yahyazadeh Jabbari, Deniz Yuce, Iman Zare, Mohammad Zoladl.

Developing methods or computational machinery

Hiwa Abubaker Ali, Ali Ahmadi, Tarik Ahmed Rashid, Sina Azadnajafabad, Mohammad Behnammoghadam, Somayeh Bohlouli, Aso Mohammad Darwesh, Mostafa Dianatinasab, Kimiya Gohari, Mehdi Hosseinzadeh, Amirreza Javadi Mamaghani, Tahereh Javaheri, Sang-woong Lee, Seyedeh Zahra Masoumi, Mokhtar Mohammadi, Ali H Mokdad, Christopher J L Murray, Mohsen Naghavi, Navid Rabiee, Amir Masoud Rahmani, Reza Rawassizadeh, Umar Saeed, Sahar Saeedi Moghaddam, Abdallah M Samy, Bay Vo.

Providing critical feedback on methods or results

Hedayat Abbastabar, Hassan Abidi, Hassan Abolhassani, Hiwa Abubaker Ali, Juan Manuel Acuna, Sima Afrashteh, Muhammad Sohail Afzal, Sajjad Ahmad, Ali Ahmadi, Haroon Ahmed, Luai A. Ahmed, Tarik Ahmed Rashid, Mostafa Akbarzadeh-Khiavi, Hanadi Al Hamad, Mohammed Khaled Al-Hanawi, Yousef Alimohamadi, Sadaf Alipour, Syed Mohamed Aljunid, Sami Almustanyir, Rajaa M Al-Raddadi, Saba Alvand, Jalal Arabloo, Morteza Arab-Zozani, Armin Aryannejad, Tahira Ashraf, Seyyed Shamsadin Athari, Sina Azadnajafabad, Amirhossein Azari Jafari, Sara Bagherieh, Mohammad Behnammoghadam, Akshaya Srikanth Bhagavathula, Somayeh Bohlouli, Wolfram Manuel Wolfram Brück, Aso Mohammad Darwesh, Mostafa Dianatinasab, Fariba Dorostkar, Ebrahim Eini, Iman El Sayed, Maysaa El Sayed Zaki, Muhammed Elhadi, Sharareh Eskandarieh, Ibtihal Fadhil, Fatemeh Farahmand, Pawan Sirwan Faris, Farshad Farzadfar, Nasrin Galehdar, Mansour Ghafourifard, Azin Ghamari, Ahmad Ghashghaee, Maryam Gholamalizadeh, Kimiya Gohari, Mohamad Golitaleb, Mohammed Ibrahim Mohialdeen Gubari, Nima Hafezi-Nejad, Rabih Halwani, Randah R Hamadeh, Samer Hamidi, Asif Hanif, Shafiul Haque, Soheil Hassanipour, Khezar

Hayat, Mohammad Heidari, Zahra Heidarymeybodi, Mohammad-Salar Hosseini, Mehdi Hosseinzadeh, Mohamed Hsairi, Elham Jamshidi, Amirreza Javadi Mamaghani, Tahereh Javaheri, Ali Kabir, Leila R Kalankesh, Leila Keikavoosi-Arani, Yousef Saleh Khader, Ejaz Ahmad Khan, Maryam Khayamzadeh, Behzad Kiani, Hamid Reza Koohestani, Bagher Larijani, Sang-woong Lee, Mozhgan Letafat-nezhad, Somayeh Livani, Farzan Madadizadeh, Ahmad R Mafi, Ata Mahmoodpoor, Marzieh Mahmoudimanesh, Mohammad-Reza Malekpour, Ahmad Azam Malik, Fariborz Mansour-Ghanaei, Mohammad Ali Mansournia, Entezar Mehrabi Nasab, Seyyedmohammadsadeq Mirmoeeni, Yousef Mohammad, Mokhtar Mohammadi, Arif Mohammed, Ali H Mokdad, Sara Momtazmanesh, Christopher J L Murray, Mohsen Naghavi, Atta Abbas Naqvi, Zuhair S Natto, Seyed Aria Nejadghaderi, Maryam Noori, Hassan Okati-Aliabad, Reza Pakzad, Zahra Zahid Piracha, Naeimeh Pourtaheri, Navid Rabiee, Alireza Rafiei, Fakher Rahim, Shahram Rahimi-Dehgolan, Amir Masoud Rahmani, Vahid Rahmanian, Mahsa Rashidi, Mohammad-Mahdi Rashidi, Reza Rawassizadeh, Mohammad Sadegh Razeghinia, Nima Rezaei, Nazila Rezaei, Negar Rezaei, Siamak Sabour, Basema Saddik, Umar Saeed, Sahar Saeedi Moghaddam, Maryam Sahebazzamani, Abdallah M Samy, Muhammad Arif Nadeem Saqib, Brijesh Sathian, Sayed Mohammad Shafiee, Saeed Shahabi, Masood Ali Shaikh, Ali Sheidaei, Rahim Ali Sheikhi, Parnian Shobeiri, Mohammad Sadegh Soltani-Zangbar, Elnaz Tabibian, Majid Taheri, Moslem Taheri Soodejani, Mohamad-Hani Temsah, Amir Tiyuri, Irfan Ullah, Sana Ullah, Sahel Valadan Tahbaz, Bay Vo, Yasir Waheed, Seyed Hossein Yahyazadeh Jabbari, Fereshteh Yazdanpanah, Deniz Yuce, Nazar Zaki, Mohammad Zoladl.

Drafting the work or revising is critically for important intellectual content

Mohsen Abbasi-Kangevari, Zeinab Abbasi-Kangevari, Hassan Abidi, Hassan Abolhassani, Eman Abu-Gharbieh, Juan Manuel Acuna, Sima Afrashteh, Muhammad Sohail Afzal, Ali Ahmadi, Sepideh Ahmadi, Luai A. Ahmed, Marjan Ajami, Mohammed Khaled Al-Hanawi, Sadaf Alipour, Sami Almustanyir, Saba Alvand, Jalal Arabloo, Morteza Arab-Zozani, Seyyed Shamsadin Athari, Sina Azadnajafabad, Amirhossein Azari Jafari, Nader Bagheri, Sara Bagherieh, Mohammad Behnammoghadam, Akshaya Srikanth Bhagavathula, Somayeh Bohlouli, Mostafa Dianatinasab, Ebrahim Eini, Iman El Sayed, Maysaa El Sayed Zaki, Iffat Elbarazi, Muhammed Elhadi, Sharareh Eskandarieh, Farshad Farzadfar, Nasrin Galehdar, Mansour Ghafourifard, Seyyed-Hadi Ghamari, Kimiya Gohari, Mohammed Ibrahim Mohialdeen Gubari, Nima Hafezi-Nejad, Rabih Halwani, Randah R Hamadeh, Shafiul Haque, Zahra Heidarymeybodi, Mohammad-Salar Hosseini, Amirreza Javadi Mamaghani, Ali Kabir, Leila R Kalankesh, Yousef Saleh Khader, Ejaz Ahmad Khan, Gulfaraz Khan, Bagher Larijani, Farzan Madadizadeh, Soleiman Mahjoub, Mohammad-Reza Malekpour, Ahmad Azam Malik, Entezar Mehrabi Nasab, Seyyedmohammadsadeq Mirmoeeni, Yousef Mohammad, Karzan Abdulmuhsin Mohammad, Esmaeil Mohammadi, Arif Mohammed, Ali H Mokdad, Sara Momtazmanesh, Abdolvahab Moradi, Paula Moraga, Abbas Mosapour, Christopher J L Murray, Mohsen Naghavi, Zuhair S Natto, Seyed Aria Nejadghaderi, Maryam Noori, Reza Pakzad, Hamidreza Pazoki Toroudi, Zahra Zahid Piracha, Navid Rabiee, Alireza Rafiei, Fakher Rahim, Shahram Rahimi-Dehgolan, Mohammad-Mahdi Rashidi, Nima Rezaei, Nazila Rezaei, Negar Rezaei, Basema Saddik, Umar Saeed, Sahar Saeedi Moghaddam, Amirhossein Sahebkar, Abdallah M Samy, Sayed Mohammad Shafiee, Saeed Shahabi, Reza Shirkoohi, Parnian Shobeiri, Elnaz Tabibian, Mohamad-Hani Temsah, Irfan Ullah, Sana Ullah, Sahel Valadan Tahbaz, Rohollah Valizadeh, Yasir Waheed, Seyed Hossein Yahyazadeh Jabbari, Deniz Yuce, Iman Zare, Mohammad Zoladl.

Managing the estimation or publications process

Sina Azadnajafabad, Ali H Mokdad, Christopher J L Murray, Mohsen Naghavi, Sahar Saeedi Moghaddam.
